# Supplementary material for: In vivo base editing of a pathogenic Eif2b5 variant improves vanishing white matter phenotypes in mice
Source: Mol Ther. 2024 Mar 7;32(5):1328–43. doi: 10.1016/j.ymthe.2024.03.009 (PMC11081866; doi:10.1016/j.ymthe.2024.03.009)
Supplement: Document S1. Supplemental methods, Figures S1–S16, and Tables S1–S9 [file mmc1.pdf]

YMTHE, Volume *32*

Supplemental Information

In vivo base editing of a pathogenic *Eif2b5* variant improves vanishing white matter phenotypes in mice

Desirée Böck, Ilma M. Revers, Anastasia S.J. Bomhof, Anne E.J. Hillen, Claire Boeijink, Lucas Kissling, Sabina Egli, Miguel A. Moreno-Mateos, Marjo S. van der Knaap, Niek P. van Til, and Gerald Schwank

Supplemental figures and legends

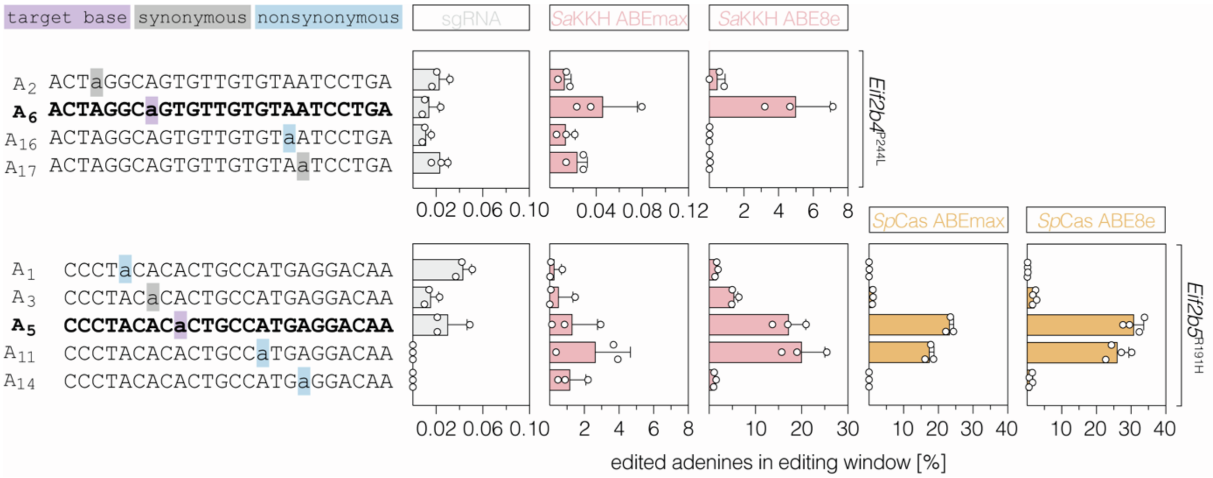

Figure S1 | On-target and bystander editing at pathogenic VWM variants in HEK cell lines. Editing outcomes at the indicated adenines at the *Eif2b4*^{P244L} (top) and *Eif2b5*^{R191H} locus (bottom) for *Sa*KKH- (sgRNA1.1 for *Eif2b4*^{P244L}; sgRNA2.1 for *Eif2b5*^{R191H}) and *Sp*Cas-ABE variants (sgRNA2.2 for *Eif2b5*^{R191H}) in HEK cell lines, where the corresponding VWM mouse variant was integrated into the genome using the PiggyBac transposon system. On-target editing (purple) as well as synonymous (gray) and nonsynonymous bystanders (blue) are highlighted in the indicated colors. The desired editing outcome with only precise on-target editing is shown in bold. The numbering of adenines (A_x) is based on their position within the combined protospacer sequence of all used ABE variants (Fig. 1A). Control samples were transfected with sgRNA only. The used ABE variants are color-coded and indicated above the respective plot. Data are displayed as means ± SD of 3 or 4 independent experiments. Related to figure 1.

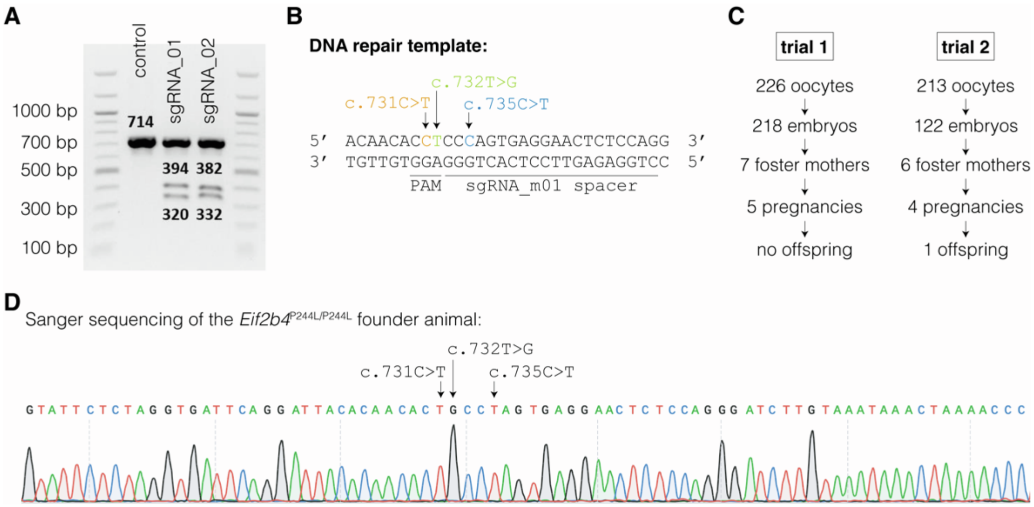

Figure S2 | Generation of a knock-in *Eif2b4*$^{\mathrm{P244L}}$ VWM mouse model. (**A**) *In vitro* validation of the activity of two sgRNAs (01 and 02) on *Eif2b4* template DNA. (**B**) Representation of the target site within the DNA repair template. Silent mutations (c.732T>G and c.735C>T) were included together with the target mutation (c.731C>T) in the repair template to avoid re-targeting of the site by the RNP complex after recombination. The PAM and spacer sequence are indicated. (**C**) Depiction of the experimental steps performed and animal numbers used for generating the *Eif2b4*$^{\mathrm{P244L}}$ mouse model. (**D**) Homozygous integration of the repair template at the intended *Eif2b4* locus in the founder animal. Related to figure 2.

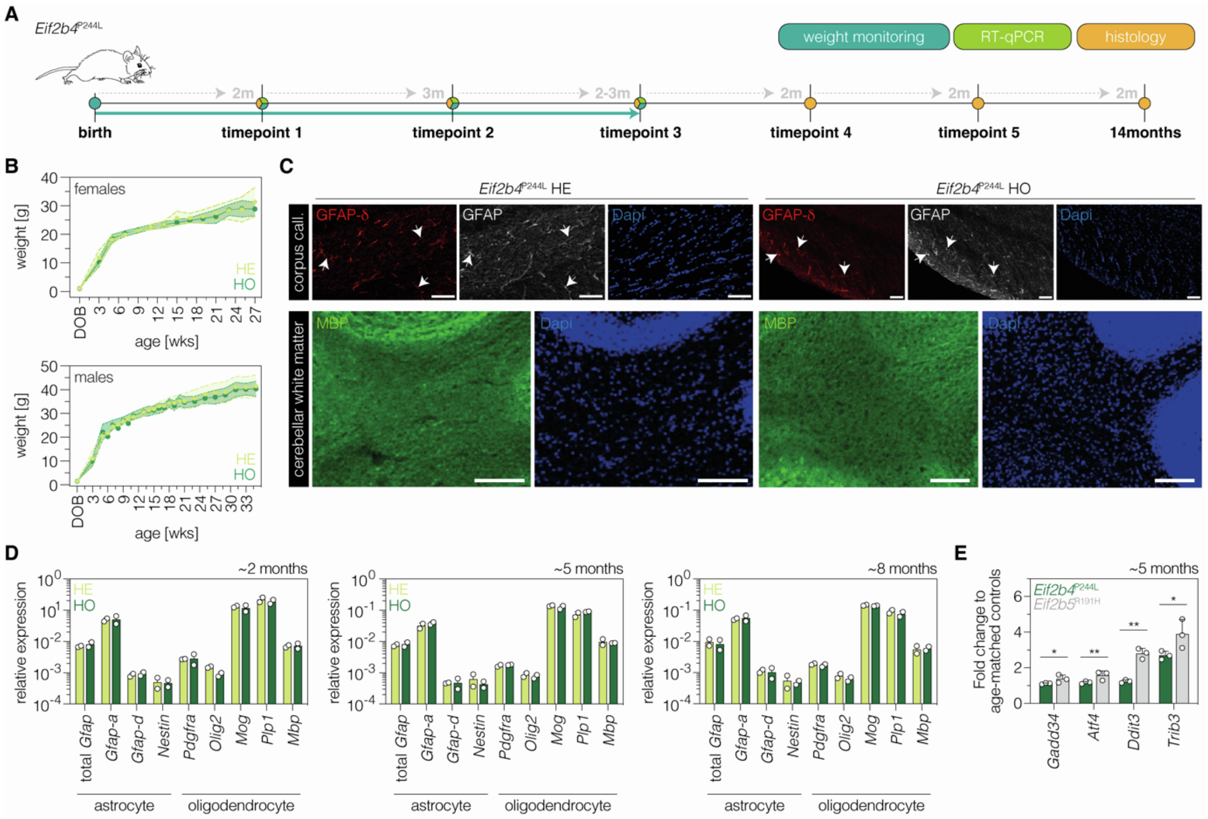

Figure S3 | Phenotypic characterization of the *Eif2b4*^{P244L} knock-in mouse model. (**A**) Schematic representation of the experimental timeline and setup. The timepoints at which weight monitoring (green), RT-qPCR analysis (light green), and histology (orange) were performed are indicated by color along the experiment timeline. (**B**) Weekly weight progression in heterozygous (HE) and homozygous (HO) *Eif2b4*^{P244L} mice (separated by gender). (**C**) Representative images showing GFAP and GFAP-δ expression in white matter astrocytes (white arrowheads) in the corpus callosum and lack of myelin vacuolization in cerebellar white matter. The *Eif2b4* genotype is indicated at the top. (**D**) Forebrain transcript levels of astrocyte (*Gfap*, *Gfap-a*, *Gfap-d*, and *Nestin*) and oligodendrocyte markers (*Pdgfra*, *Olig2*, *Mog*, *Plp1*, and *Mbp*). (**E**) Comparison of ISR transcript levels in homozygous (HO) *Eif2b4*^{P244L} and *Eif2b5*^{R191H} mice at 8 months of age. Age-matched healthy controls were used for quantification of fold changes in the corresponding model. Transcript levels were normalized to *Akt*. Data are displayed as means ± range of 2 animals per group (A and D), means ± SD of 3 animals per group (B and E), and were analyzed using an unpaired two-tailed Student's t-test (E; $*P<0.05$; $**P<0.005$). Unless otherwise indicated, statistical differences were not significant. DOB, date of birth; wks, weeks; m, month(s), g, gram; wt, wild type; cc, corpus callosum; HE, heterozygous; HO, homozygous; *Gfap*/GFAP, glial fibrillary acidic protein; *Gfap-d*/GFAP-δ, delta isoform of glial fibrillary acidic protein; *Gfap-a*, alpha isoform of glial fibrillary acidic protein; *Pdgfra*, platelet-derived growth factor receptor alpha; *Olig2*, oligodendrocyte transcription factor 2; *Mog*, myelin oligodendrocyte glycoprotein; *Plp1*, proteolipid protein 1; *Mbp*, myelin basic protein; *Nestin*, neuroepithelial stem cell protein. *Trib3*, Tribbles Pseudokinase 3; *Atf4*, activating transcription factor 4; *Ddit3*, DNA damage inducible transcript 3; Gadd34, growth arrest and DNA damage-inducible protein 34. Scale bars, 100µm. Related to figure 2.

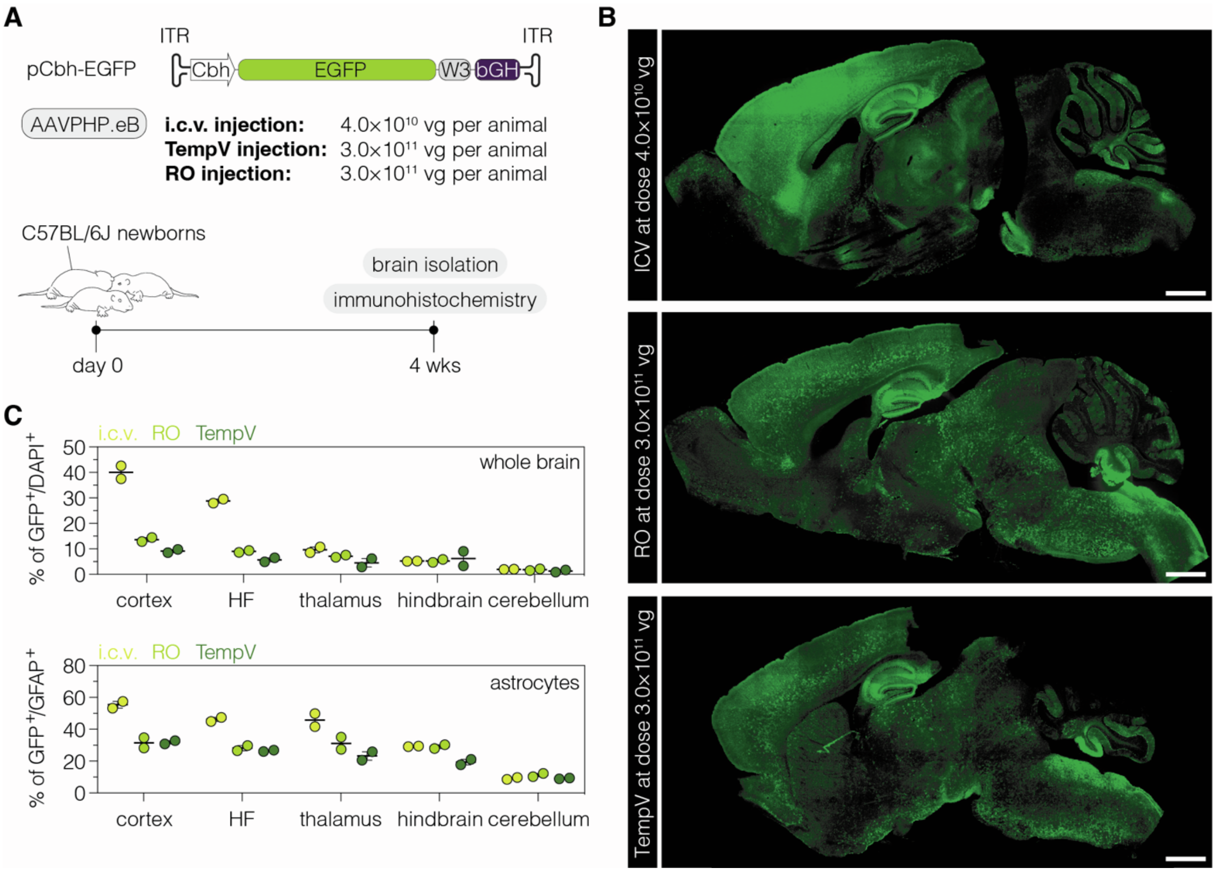

Figure S4 | AAV-PHP.eB transduction efficiencies in the newborn brain upon systemic or intracranial administration. (**A**) Schematic representation of the experimental setup and timeline for analysis of transduction efficiencies in the brain. Maximum AAV doses were injected for each application route. (**B**) Representative whole-brain overview images of EGFP fluorescence after 30 days of expression. AAV particles, expressing EGFP under the Cbh promoter, were delivered to newborn mice via the indicated route and at the described dose. (**C**) Quantifications of total transduction efficiency (top, displayed as percentage of $GFP^{+}/DAPI^{+}$), and astrocyte transduction efficiency (bottom, displayed as percentage of $GFP^{+}/GFAP^{+}$) in different brain regions. Data are displayed as means ± range of 2 mice per group. Wks, weeks; i.c.v., intracerebroventricular; RO, retro-bulbar; TempV, temporal vein; vg, vector genomes; EGFP, enhanced green fluorescent protein; HF, hippocampal formation. Scale bars, 1000µm. Related to figure 2.

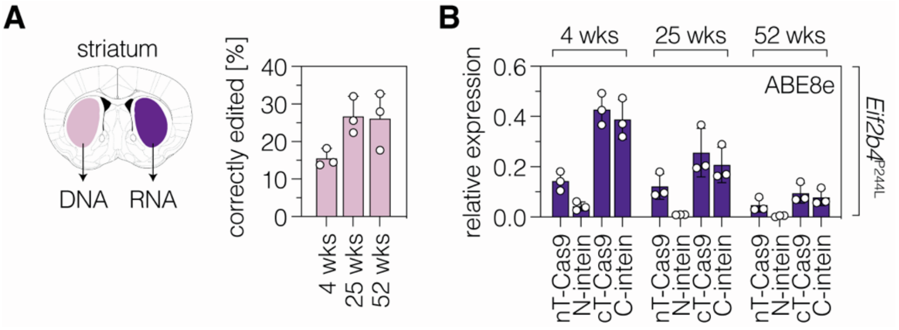

Figure S5 | Time course of editing rates and ABE8e expression in mouse striata over time. (**A,B**) Editing rates (A) and ABE8e transcript levels (B) of n- and c-terminal AAV preparations in striata of *Eif2b4*^{P244L} mice (n=3). Transcripts were normalized to *Gapdh*. Corresponding editing rates of the same mice are shown for reference. Tissue regions used for DNA and RNA isolations are shown on the brain atlas (left).[65] Data are displayed as means ± SD of 3 mice per group. nT, N-terminal AAV construct; cT, C-terminal AAV construct; wks, weeks. Related to figure 3.

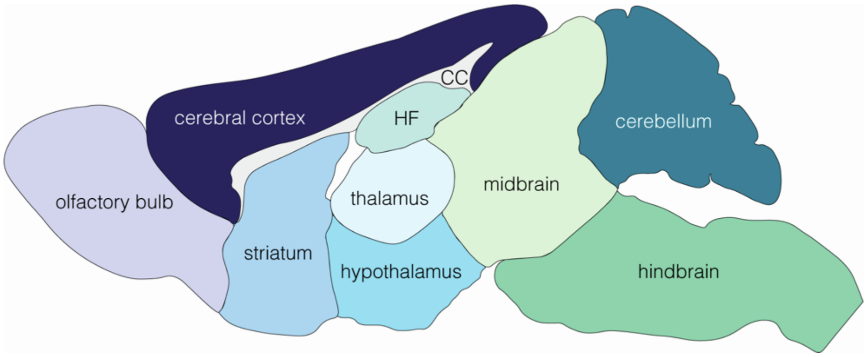

Figure S6 | Dissection of the mouse brain into 10 distinct regions. Schematic representation marking the dissection borders for isolation of the olfactory bulb, cerebral cortex, corpus callosum (CC), hippocampal formation (HF), thalamus, striatum, hypothalamus, midbrain, hindbrain, and cerebellum. Regions are depicted approximately to scale. Related to figure 5.

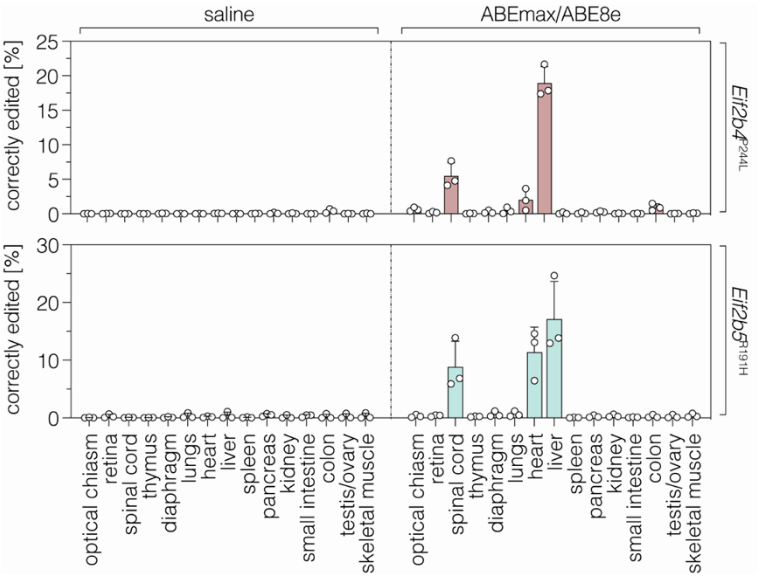

Figure S7 | *In vivo* base editing at the *Eif2b4* and *Eif2b5* locus outside the brain. On-target editing rates at the *Eif2b4* (top) and *Eif2b5* (bottom) locus in saline- (left) and ABEmax/ABE8e-treated mice (right). Data are displayed as means ± SD of 3 animals per group and genotype. Related to figure 5.

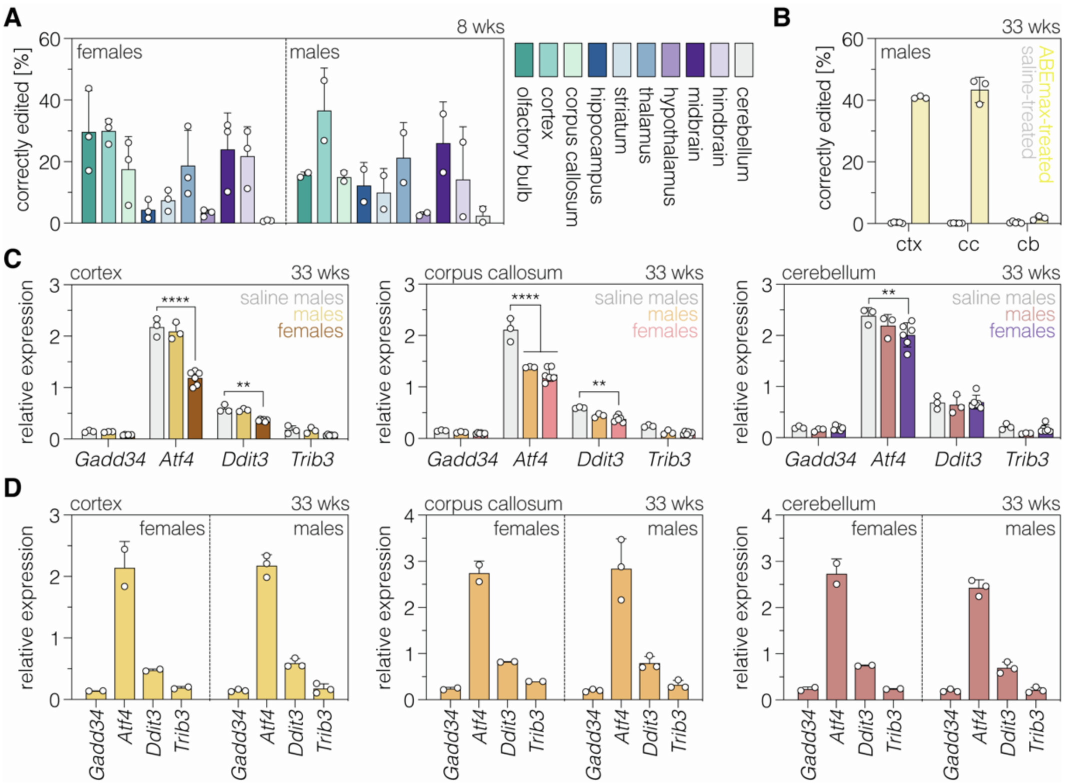

Figure S8 | Base editing and transcript levels of ISR regulators across different brain regions in female and male *Eif2b5*^{R191H} mice. (**A**) Editing rates at the *Eif2b5* locus in different brain regions at 8 weeks post-treatment in female (n=3) and male mice (n=2). (**B,C**) Endpoint editing rates (B) and corresponding transcript levels of ISR regulators (C) in the cortex (ctx), corpus callosum (cc), and cerebellum (cb) of saline- and ABEmax-treated male *Eif2b5*^{R191H} mice (n=3 mice per group). Transcript levels of ABEmax-treated female *Eif2b5*^{R191H} mice are shown for comparison (n=6 mice per group) in (C). (**D**) Transcript levels of ISR regulators in saline-treated female (n=2) and male *Eif2b5*^{R191H} mice (n=3). All transcripts were normalized to *Akt* (C and D). Data are displayed as means ± SD of 2-6 animals per group and were analyzed using a two-way ANOVA with Sidak's multiple comparisons test (C; $^{**}P<0.005$; $^{****}P<0.0001$). Unless otherwise indicated, statistical differences were not significant. ctx, cortex; cc, corpus callosum; cb, cerebellum. Related to figure 5.

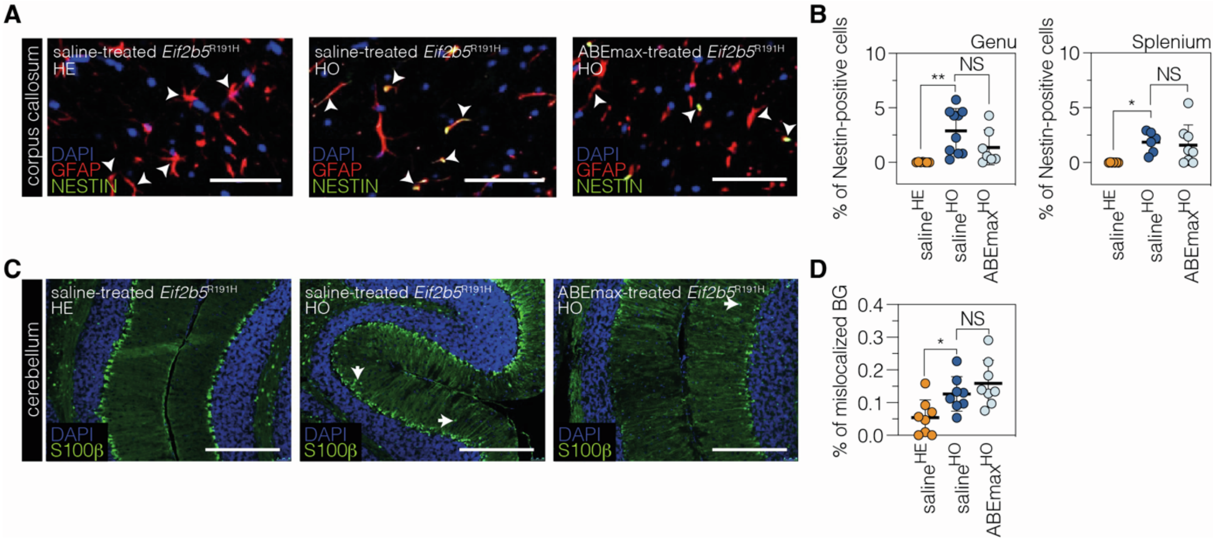

Figure S9 | Histological analysis of VWM neuropathologies in saline- and ABEmax-treated female *Eif2b5*^{R191H} mice. (**A,B**) Representative fluorescence micrographs (A) and the corresponding quantifications (B) of immature astrocytes (NESTIN/GFAP double positive) in the anterior (Genu) and posterior (Splenium) part of the corpus callosum in saline-treated heterozygous (n=7; HE), saline-treated homozygous (n=10; HO), and ABEmax-treated homozygous *Eif2b5*^{R191H} female mice (n=7; HO). Astrocytes are indicated by a white arrowhead. (**C,D**) Representative fluorescence micrographs (C) and the corresponding quantifications (D) of mislocalized Bergmann glia (S100β-positive) in the cerebellum of saline-treated heterozygous (n=8; HE), saline-treated homozygous (n=8; HE), and ABEmax-treated homozygous *Eif2b5*^{R191H} mice (n=8; HO). Mislocalized Bergmann glia are indicated by a white arrow. Scale bars, 50µm (A,C). Data are displayed as means ± SD of 8 mice per group and were analyzed using a one-way ANOVA with Dunnett's multiple comparisons test (NS, $P>0.05$; $^*P<0.05$; $^{**}P<0.005$). Each datapoint represents one animal. NS, not significant; HE, heterozygous; HO, homozygous. Related to figure 5.

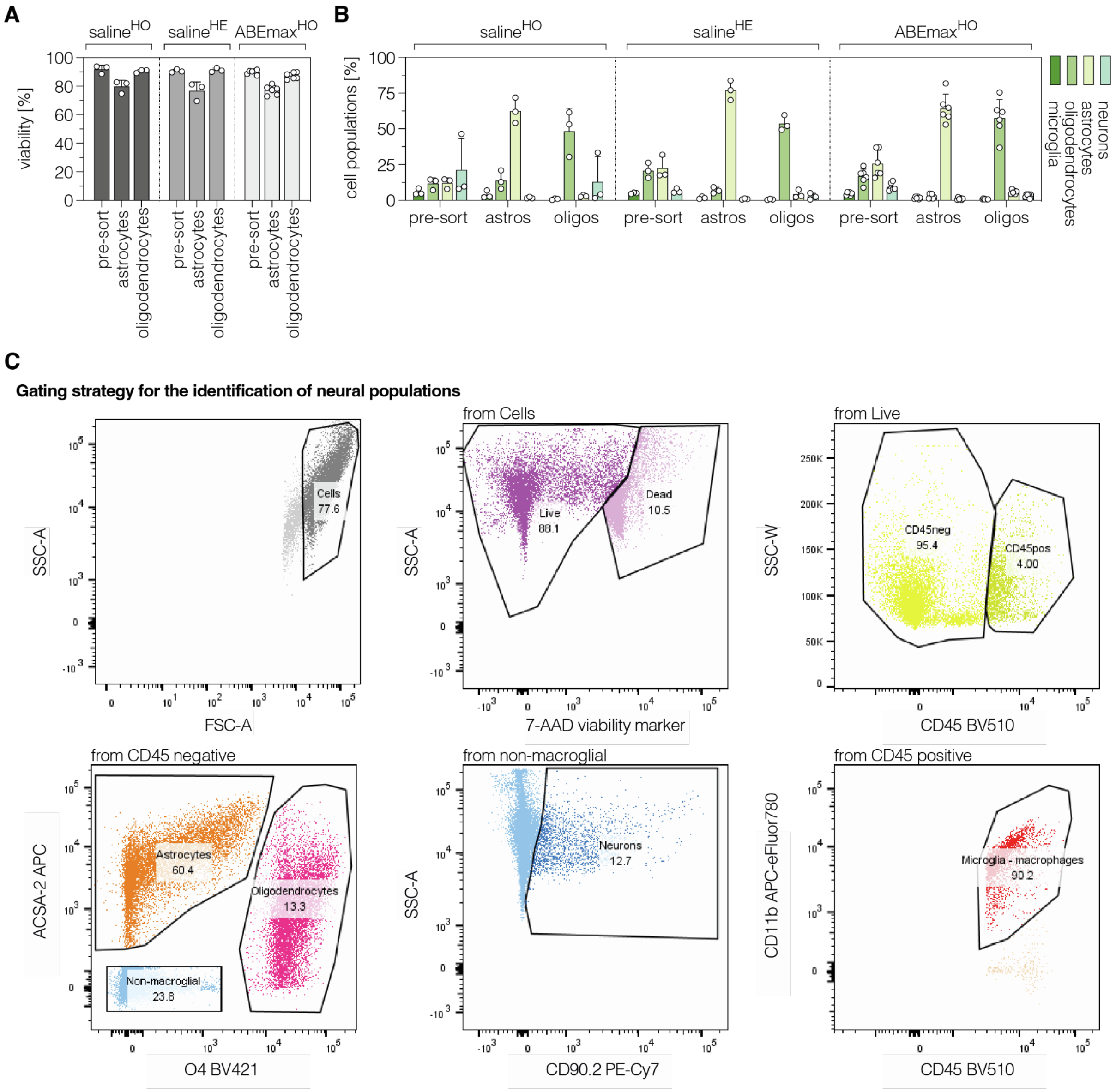

Figure S10 | Flow cytometry validation of *Eif2b5*^{R191H} brain cell populations. (**A**) Viability of cells before magnetic sorting (pre-sort), purified astrocytes, and purified oligodendrocytes isolated from *Eif2b5*^{R191H} mice. (**B**) Quantification of neuronal, astrocytic, oligodendrocytic, and microglia/macrophage populations before (pre-sort) and after magnetic sorting (“astros” and “oligos”) by flow cytometry. (**C**) Gating strategy used for the quantification and identification of neural populations (neurons, astrocytes, oligodendrocytes, and microglia/macrophages). All mice were females except for one male in the saline-treated heterozygous control group (A,B). HO, homozygous; HE, heterozygous; astros, astrocytes; oligos, oligodendrocytes. Data are displayed as means ± SD of 3 or 6 mice per group. Related to figure 5.

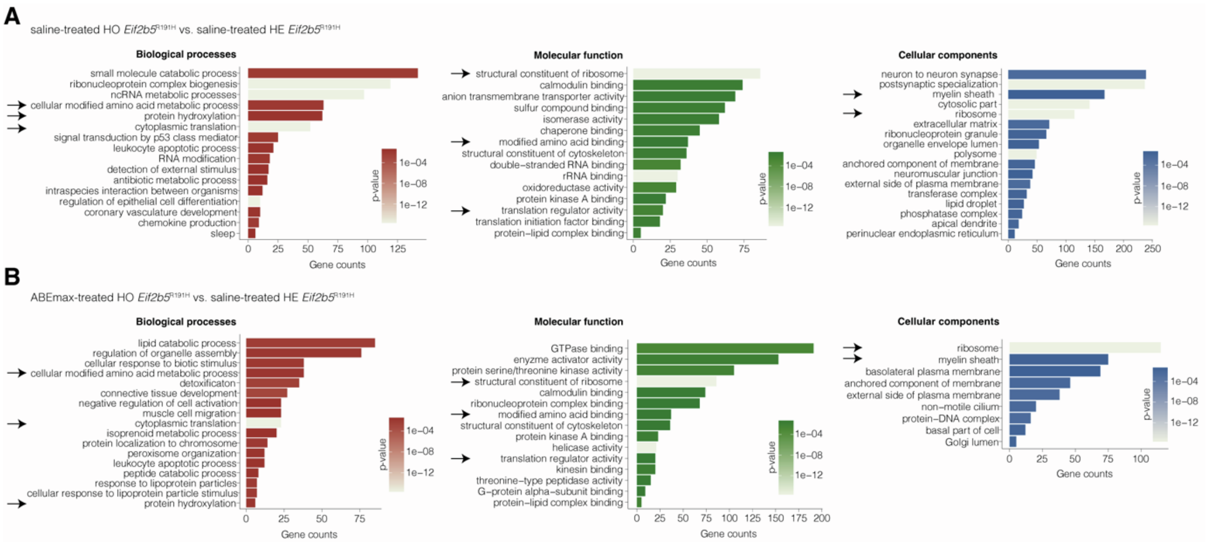

Figure S11 | Gene ontology analyses of saline- and ABEmax-treated female *Eif2b5*R191H mice. (**A,B**) Gene ontology analyses of differentially expressed proteins in saline- (A; n=3) or ABEmax-treated female homozygous (HO) *Eif2b5*R191H (B) mice (n=6). Both groups were compared to healthy heterozygous (HE) *Eif2b5*R191H controls (n=3 female mice). Enriched terms are ranked by gene count and are color-coded with respect to their *p*-value. Terms associated with ribosome, translation, and myelination, which were found in both conditions, are indicated with a black arrow. HO, homozygous; HE, heterozygous. Related to figure 5.

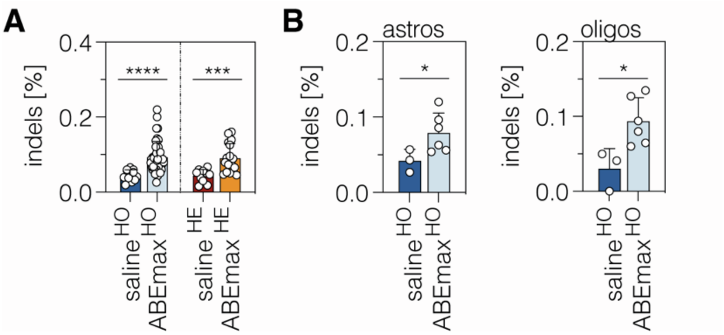

Figure S12 | Indel formation in saline- and ABEmax-treated *Eif2b5*^{R191H} mice. (**A,B**) Indel formation at the *Eif2b5* target site in saline- and ABEmax-treated animals (A; olfactory bulbs from female and male mice at experimental endpoints) or MACS-purified astrocytes and oligodendrocytes (B; only female mice at experimental endpoints). Indel quantification window spans from the nicking site to the deamination site. Data are displayed as means ± SD of at least 3 animals per group and were analyzed using an unpaired two-tailed Student's t-test (A,B; **P*<0.05; ****P*<0.0005; *****P*<0.0001). HE, heterozygous; HO, homozygous; astros, astrocytes; oligos, oligodendrocytes. Related to figure 6.

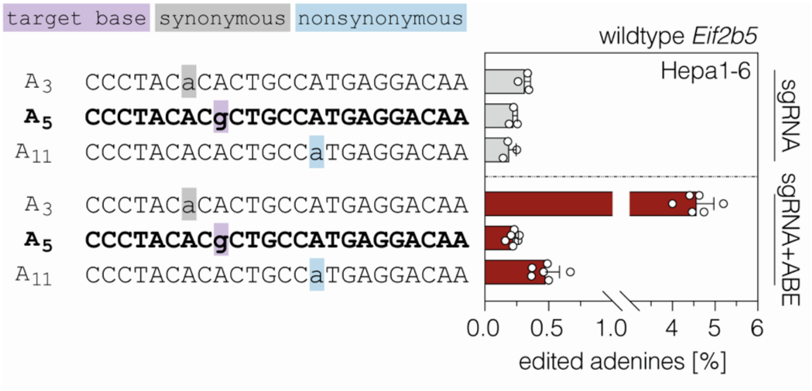

Figure S13 | *In vitro* bystander editing at the murine *Eif2b5* wildtype locus. Bystander editing at the wildtype locus (1 mismatch at position A_5) in murine Hepa1-6 cells. Data are displayed as means ± SD of at least 3 independent experiments. Related to figure 6.

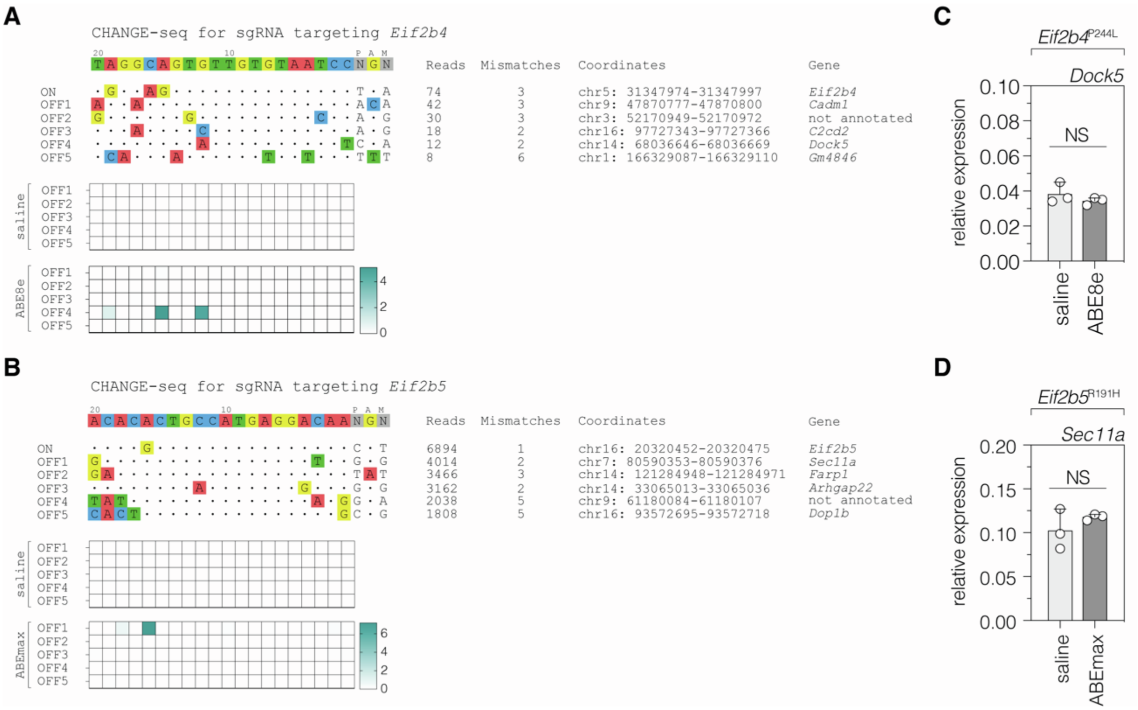

Figure S14 | *In vivo* base editing does not induce off-target editing in coding regions. (**A,B**) The top 5 off-target sites for the sgRNA protospacers targeting either the *Eif2b4* (A) or *Eif2b5* (B) locus were experimentally identified by CHANGE-seq.[43] Only the top 5 off-target sites are shown. Deep sequencing results of the top 5 off-target sites are shown for the *Eif2b4* (A) or *Eif2b5* sgRNA (B) in saline- and ABEmax/ABE8e-treated mice (>15'000 reads per site, n=3 mice per group). Data are displayed as means. (**C,D**) Transcript levels of off-target 4 (*Dock5*) in saline- and ABE8e-treated *Eif2b4*^{P244L} mice (C) and off-target 1 (*Sec11a*) in saline- and ABEmax-treated *Eif2b5*^{R191H} mice (D). Transcript levels were normalized to *Akt*. Data are displayed as means ± SD of 3 animals per group (A-D) and were analyzed using an unpaired two-tailed Student's t-test (C,D). Related to figure 6.

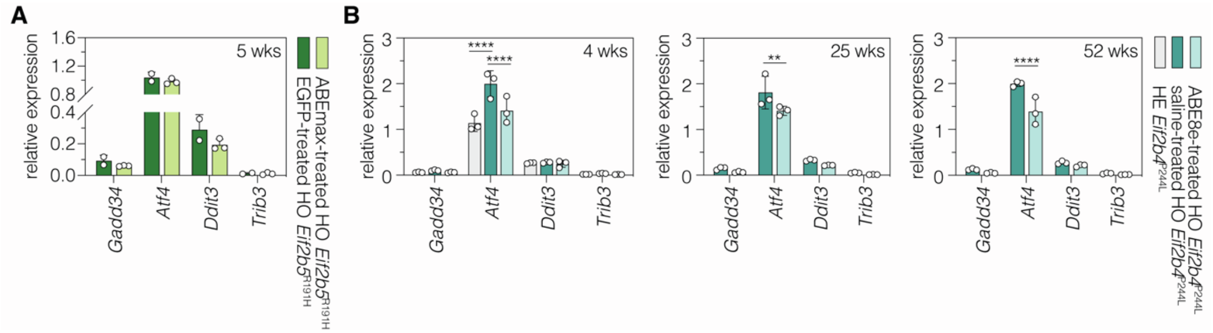

Figure S15 | AAV delivery and transgene expression do not upregulate the ISR in *Eif2b4*$^{\mathrm{P244L}}$ and *Eif2b5*$^{\mathrm{R191H}}$ mice. (**A**) Transcript levels of ISR regulators in homozygous *Eif2b5*$^{\mathrm{R191H}}$ animals at 5 weeks after delivery of AAV-PHP.eB particles, expressing either EGFP or ABEmax under the Cbh promoter, at a dose of 8×10^{10} vg per animal via ICV injection. (**B**) Transcript levels of ISR regulators in homozygous *Eif2b4*$^{\mathrm{P244L}}$ animals at 4, 25, and 52 weeks after delivery of AAV-PHP.eB particles, expressing ABE8e under the Cbh promoter, at a dose of 4.8×10^{10} vg per animal via ICV injection. Transcript levels of healthy heterozygous *Eif2b4*$^{\mathrm{P244L}}$ (4 weeks) and saline-treated homozygous *Eif2b4*$^{\mathrm{P244L}}$ (4, 25, and 52 weeks) mice are shown for comparison. All transcripts were normalized to *Akt*. Data are displayed as means ± range (A) or means ± SD (B) of 2 or 3 animals per group. HO, homozygous. Related to figure 6.

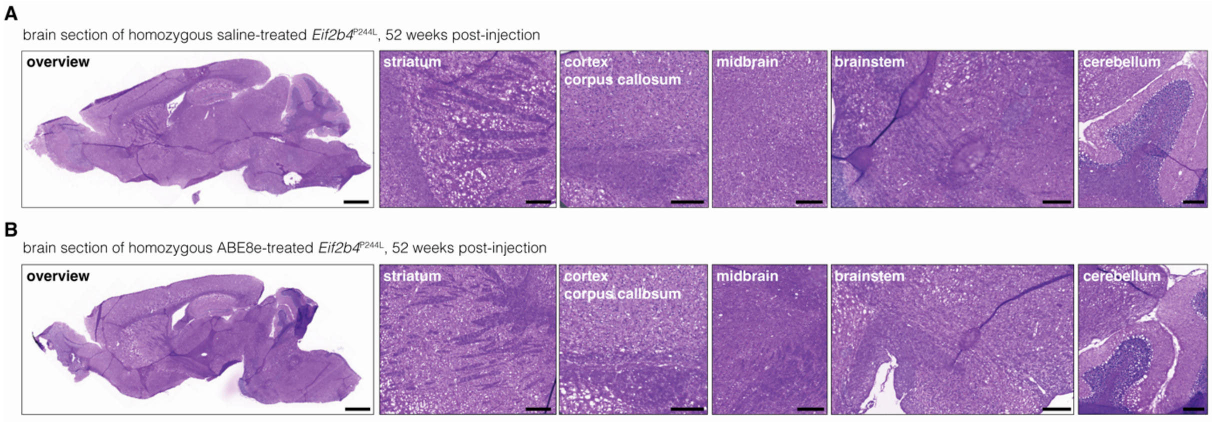

Figure S16 | *In vivo* base editing does not induce tissue damage in brains of homozygous *Eif2b4*$^{\mathbf{P244L}}$ mice. (**A,B**) Representative whole brain overview images and close-ups of distinct regions of hematoxylin- and eosin-stained brain sections of saline- (A) and ABE8e-treated homozygous mice (B). Scale bars, 1000µm (overview); 100µm (magnified view). Related to figure 6.

Supplemental tables

Table S1 | On- and off-target efficiency predictions for sgRNAs designed using the indicated algorithms. A score above 50 is considered good. IDT, integrated DNA technologies; Bench, Benchling; ON-T, on-target efficiency; OFF-T, off-target efficiency.

sgRNA_ID	sequence (5′ to 3′)	ON-T^{IDT}	ON-T^{Bench}	OFF-t^{CRISPOR}	OFF-t^{IDT}	OFF-t^{Bench}
sgRNA_01	CTGGAGAGTTCCTCACTGGG	53/100	67/100	75/100	18/100	36/100
sgRNA_02	CTCCCAGTGAGGAACTTCTCC	69/100	54/100	77/100	29/100	39/100

Table S2 | List of antibodies used in this study.

antibody	clone/ID	host	dilution	application
ACSA-2/APC	IH3-18A3	rat	0.75µg/mL	flow cytometry
CD11b/APC-eFluor™ 780	M1/70	rat	2µg/mL	flow cytometry
CD45/BV 510™	30-F11	rat	2µg/mL	flow cytometry
CD90.2/PE(Cy7)	30-H12	rat	2µg/mL	flow cytometry
O4/Biotin	O4	mouse	2.2µg/mL	flow cytometry
NEUN	EPR12763	rabbit	1:1'000	histology
GFAP	ab95231	chicken	1:1'500	histology
NESTIN	25/NESTIN	mouse	1:500	histology
GFAP	Z0334	rabbit	1:1'000	histology
S100β	15146-1-AP	rabbit	1:1'000	histology
anti-rabbit A488	JIR-711-545-152	donkey	1:1'000	histology
anti-chicken Cy5	JIR-703-175-155	donkey	1:500	histology
anti-rabbit A594	A31632	goat	1:1'000	histology
anti-mouse A488	A31620	goat	1:1'000	histology

Table S3 | Reference nucleotide sequences of HTS amplicons.

amplicon_ID	amplicon sequence (5′ to 3′)
EIF2B4-PB	GGATCCACTAGTAACGGCCGCCAGTGTGCTGGAATTCGAAGCAATGACTCTCACAATCT<br>GAACACTTACTTCCCTAGTGAAGCTTACCCCTGTCCCATAGTAGAGATCTTCCTTAAGG<br>GGTGTTTTGTATTCTCTAGGTGATTCAGGATTACACAACACtgCCtAGTGAGGAACTCT<br>CCAGGGATCTTGTAAATAAACTAAAACCCTACATCAGGTGAGCACAGCC
EIF2B5-PB	GATCACAGAGTTGGGCAGACTAACTGTGCCTCTGGTTCTTAATAGGTTAAGAAGGAAGC<br>TAGAAAAAAATGTCTCTGTGATGACAATGGTCTTCAAAGAGTCGTCACCCAGCCACCCT<br>ACACACTGCCATGAGGACAACGTGGTGATGGCTGTGGACAGCGCCACCAACAGGGTTCT<br>TCACTTCCAGAAGACCCAAGG
EIF2B4-mouse	GTGAAGCTTACCCCTGTCCCATAGTAGAGATCTTCCTTAAGGGGTGTTTTGTATTCTCT<br>AGGTGATTCAGGATTACACAACACtgCCtAGTGAGGAACTCTCCAGGGATCTTGTAAAT<br>AAACTAAAACCCTACATCAGGTGAGCACAGCC
EIF2B5-mouse	GGACAAGACTTAGGGACCAGCAGAGTCCTAAGACTAACTGTGCCTCTGGTTCTTAATAG<br>GTTAAGAAGGAAGCTAGAAAAAAATGTCTCTGTGATGACAATGGTCTTCAAAGAGTCGT<br>CACCCAGCCACCCTACACACTGCCATGAGGACAACGTGGTGATGGCTGTGGACAGCGCC<br>ACCAACAGGGTTCTTCACTTCCAGAAGACCCAAGGCCTCCGGCGCTTTTCCTTTCCATT

Supplemental Methods

Table S4 | Oligos used for cloning of sgRNA plasmids.

oligo name	sgRNA_ID	sequence (5′ to 3′)
sgRNA01_P244L-NGN_fwd	sgRNA-01	CACCgTaGGcaGTGTTGTGTAATCC
sgRNA01_P244L-NGN_rev		AAACGGATTACACAACACtgCCtAc
sgRNA02_P244L-NNGRRT_fwd	sgRNA-02	CACCgACTaGGcaGTGTTGTGTAATC
sgRNA02_P244L-NNGRRT_rev		AAACGATTACACAACACtgCCtAGTc
sgRNA03_R191H_NNNRRT_fwd	sgRNA-03	CACCGTACACACTGCCATGAGGACAA
sgRNA03_R191H_NNNRRT_rev		AAACTTGTCCTCATGGCAGTGTGTAC
sgRNA04_R191H_NGG_fwd	sgRNA-04	CACCGACACTGCCATGAGGACAACG
sgRNA04_R191H_NGG_rev		AAACCGTTGTCCTCATGGCAGTGTC
sgRNA05_R191H_NGN_fwd	sgRNA-05	CACCGACACACTGCCATGAGGACAA
sgRNA05_R191H_NGN_rev		AAACTTGTCCTCATGGCAGTGTGTC

Table S5 | Oligos used for cloning of AAV plasmids.

oligo name	sequence (5’ to 3’)
pCMV-N_fwd	CGGCCTCTAGATCAGGGTACcgacattgattattgactagttattaatag
pCMV-N_rev	ggtggcggccagctctgcttatatagacc
Tad8e_fwd	aagcagagctggccgccaccatgaaacg
Tad8e_rev	cctccggagttgatggagctctgggccttcttctgagc
pCMV-C_fwd	ACTAGGGGTTCCTGCGGCCTCTAGATCAGGGTACCgacattgattattg
pCMV-C_rev	CTGTCCGTTTCATGGTGGCgctagcagctctgcttatatagacctc
pCbhTad8e_fwd	tcactttttttcaggttggaccggtgccaccatgaaacggacag
pCbhTad8e_rev	ggtgtccttgctcagctgcagtttggcatcctcggccag
pCbh-cSpG_fwd	agaatggctttatcgccagcaattgcttcgactccgtggaaatctccgg
pCbh-cSpG_rev	tcctcttcttcttgggctcgaattcgctgccgtcggcggttct

Table S6 | Oligos used for validation of sgRNAs and *Eif2b4*^{P244L} knock-in.

oligo name	sequence (5′ to 3′)
19017.P1_fwd	CATCCCATCCTCTGTGATCC
19017.P4_rev	TGCTCCCCTAAGCCTGACTA
19017.P5_fwd	GATGGTCCTCTCTGGGACAA
19017.P6_rev	GTCAGGAAGCTGCAACAACA

Table S7 | Oligos used for RT-qPCR.

oligo name	sequence (5′ to 3′)
Gfap-RTqPCR_fwd	ACATCGAGATCGCCACCTACA
Gfap-RTqPCR_rev	CCACGATGTTCCTCTTGAGGTG
Gfapα-RTqPCR_rev	CCTTCACATCACCACGTCCTTG
Gfapδ-RTqPCR_rev	CCATTTTCAATCTGGTGAGCCTG
Nestin-RTqPCR_fwd	CTACAGAGTCAGATCGCTCAG
Nestin-RTqPCR_rev	AGCAGAGTCCTGTATGTAGC
Mbp-RTqPCR_fwd	ATTCACCGAGGAGAGGCTGGAA
Mbp-RTqPCR_rev	TGTGTGCTTGGAGTCTGTCACC
Plp1-RTqPCR_fwd	CCTAGCAAGACCTCTGCCAGTA
Plp1-RTqPCR_rev	GGACAGAAGGTTGGAGCCACAA
Mog-RTqPCR_fwd	GATGAAGGAGGCTACACCTGCT
Mog-RTqPCR_rev	CGTAGGCACAAGTGCGATGAGA
Olig2-RTqPCR_fwd	ATGCACGACCTCAACATCGCCA
Olig2-RTqPCR_rev	ACCAGTCGCTTCATCTCCTCCA
Pdgfrα-RTqPCR_fwd	CTGGAGAAGTGAGAAACAAAGG
Pdgfrα-RTqPCR_rev	TGGACAGAAATGGTGACTC
Gapdh-RTqPCR_fwd	CATCACTGCCACCCAGAAGACTG
Gapdh-RTqPCR_rev	ATGCCAGTGAGCTTCCCGTTCAG
Cas9-N-RTqPCR_fwd	aaacagcagattcgcctgga
Cas9-N-RTqPCR_rev	tcatccgctcgatgaagctc
Nintein-RTqPCR_fwd	ttcatgacagtggatggcca
Nintein-RTqPCR_rev	ttcttgggctcaaactcact
Cas9-C-RTqPCR_fwd	atgggcgggatatgtacgtg
Cas9-C-RTqPCR_rev	ggtcagcaccttgttgtcga
Cintein-RTqPCR_fwd	agtcaccaaagaagaagcgga
Cintein-RTqPCR_rev	gaagttgtgatcccgctcca
Hprt-RTqPCR_fwd	GTTGGGCTTACCTCACTGCT
Hprt-RTqPCR_rev	TAATCACGACGCTGGGACTG
Akt-RTqPCR_fwd	AAGAAGGAGGTCATCGTCGC
Akt-RTqPCR_rev	GGTCGTGGGTCTGGAATGAG
Gadd34-RTqPCR_fwd	GCTCAGATTGTTCAAAGCCC
Gadd34-RTqPCR_rev	CTTTCTCAGCGAAGTGTACC
Atf4-RTqPCR_fwd	CTAGGTCTCTTAGATGACTATCTG
Atf4-RTqPCR_rev	CTCCAACATCCAATCTGTCC
Ddit3/Chop-RTqPCR_fwd	CTGGTATGAGGATCTGCAGG
Ddit3/Chop-RTqPCR_rev	TTGATTCTTCCTCTTCGTTTCC
Trib3-RTqPCR_fwd	TGTCTTCAGCAACTGTGAGAGGACGAAG
Trib3-RTqPCR_rev	GTAGGATGGCCGGGAGCTGAGTATC

Table S8 | Oligos used for deep sequencing.

oligo name	oligo sequence (5′ to 3′)
HTS-EIF2B4-PB_fwd	CTTTCCCTACACGACGCTCTTCCGATCTNNNNNNNGGATCCACTAGTAACGGCCG
HTS-EIF2B4-PB_rev	GGAGTTCAGACGTGTGCTCTTCCGATCTNNNNNNNTCCCTGGAGAGTTCCTCACT
HTS-EIF2B5-PB_fwd	CTTTCCCTACACGACGCTCTTCCGATCTNNNNNNNGATCACAGAGTTGGGCAGAC
HTS-EIF2B5-PB_rev	GGAGTTCAGACGTGTGCTCTTCCGATCTNNNNNNNCCTTGGGTCTTCTGGAAGTG
HTS-EIF2B4m_fwd	CTTTCCCTACACGACGCTCTTCCGATCTNNNNNNNGTGAAGCTTACCCCTGTCCC
HTS-EIF2B4m_rev	GGAGTTCAGACGTGTGCTCTTCCGATCTNNNNNNNGGCTGTGCTCACCTGATGTA
HTS-EIF2B5m_fwd	CTTTCCCTACACGACGCTCTTCCGATCTNNNNNNNGGACAAGACTTAGGGACCAG
HTS-EIF2B5m_rev	GGAGTTCAGACGTGTGCTCTTCCGATCTNNNNNNNAATGGAAAGGAAAAGCGCC

Table S9 | Oligos used for CHANGE-seq.

oligo name	oligo sequence (5′ to 3′)
amplCHANGE-2b4_fwd	CCATTCCCTAAGACTGCCCC
amplCHANGE-2b4_rev	GGGAGGTCAGAAGATGCTGC
amplCHANGE-2b5_fwd	GAGGAACACAGGTCAGGCTG
amplCHANGE-2b5_rev	ATTACAGACCCCCGAGAGCT
HTS-2b4_OFF1_fwd	CTTTCCCTACACGACGCTCTTCCGATCTTGTCACTGCGGTTTCTCG
HTS-2b4_OFF1_rev	GGAGTTCAGACGTGTGCTCTTCCGATCTCCATACACTGCCTACCGCC
HTS-2b4_OFF2_fwd	CTTTCCCTACACGACGCTCTTCCGATCTTCGACCAGTTAGAGACCAGCCT
HTS-2b4_OFF2_rev	GGAGTTCAGACGTGTGCTCTTCCGATCTGGCCCATGTGCCAGAAAATAAGA
HTS-2b4_OFF3_fwd	CTTTCCCTACACGACGCTCTTCCGATCTACAGTTGTTCTCATTCATGTGGGA
HTS-2b4_OFF3_rev	GGAGTTCAGACGTGTGCTCTTCCGATCTAAAGTTGGGCATGTTGGAATACA
HTS-2b4_OFF4_fwd	CTTTCCCTACACGACGCTCTTCCGATCTTTTTCCAAGTTTCAATTAACTGCCT
HTS-2b4_OFF4_rev	GGAGTTCAGACGTGTGCTCTTCCGATCTACCTTACCTGTTTGTTGGGGAG
HTS-2b4_OFF5_fwd	CTTTCCCTACACGACGCTCTTCCGATCTCCCATGTAGTATCCAAATGTCACC
HTS-2b4_OFF5_rev	GGAGTTCAGACGTGTGCTCTTCCGATCTAGCATACTGATTACTTCTTGATGGT
HTS-2b5_OFF1_fwd	CTTTCCCTACACGACGCTCTTCCGATCTAGAGAGAGAGACACCATGACCA
HTS-2b5_OFF1_rev	GGAGTTCAGACGTGTGCTCTTCCGATCTATCCCAAGCTATACCCAGTGG
HTS-2b5_OFF2_fwd	CTTTCCCTACACGACGCTCTTCCGATCTGCCACACTTCTGACCTGACA
HTS-2b5_OFF2_rev	GGAGTTCAGACGTGTGCTCTTCCGATCTCTCAGGCTGCAGGTGACTAC
HTS-2b5_OFF3_fwd	CTTTCCCTACACGACGCTCTTCCGATCTTCTCCGAGTTGCCCTAGAGT
HTS-2b5_OFF3_rev	GGAGTTCAGACGTGTGCTCTTCCGATCTAGACAAGGCTTACTTCGGGG
HTS-2b5_OFF4_fwd	CTTTCCCTACACGACGCTCTTCCGATCTCCATAGATGGGGAGAACCCAA
HTS-2b5_OFF4_rev	GGAGTTCAGACGTGTGCTCTTCCGATCTAACACACTCTCCACTCTTGACTAA
HTS-2b5_OFF5_fwd	CTTTCCCTACACGACGCTCTTCCGATCTGTCTACCGTGGAGCATGAGG
HTS-2b5_OFF5_rev	GGAGTTCAGACGTGTGCTCTTCCGATCTCACTCTGCTCTGCTTCAGCC

Supplemental References

65. Paxinos, G, Franklin, KBJ (2001). The Mouse Brain in Stereotaxic Coordinates, 2nd edition. *Acad Press*.
