# Supplementary material for: In vivo base editing of a pathogenic Eif2b5 variant improves vanishing white matter phenotypes in mice
Source: Mol Ther. 2024 Mar 7;32(5):1328–43. doi: 10.1016/j.ymthe.2024.03.009 (PMC11081866; doi:10.1016/j.ymthe.2024.03.009)
Supplement: Document S2. Article plus supplemental information [file mmc2.pdf]

Molecular Therapy
Original Article

In vivo base editing of a pathogenic *Eif2b5* variant improves vanishing white matter phenotypes in mice

Desirée Böck,[1,7] Ilma M. Revers,[2,7] Anastasia S.J. Bomhof,[2] Anne E.J. Hillen,[2] Claire Boeijink,[2] Lucas Kissling,[1] Sabina Egli,[1] Miguel A. Moreno-Mateos,[4,5] Marjo S. van der Knaap,[2,3] Niek P. van Til,[2,3,6] and Gerald Schwank[1,6]

[1]Institute of Pharmacology and Toxicology, University of Zurich, 8057 Zurich, Switzerland; [2]Department of Child Neurology, Amsterdam Leukodystrophy Center, Emma Children's Hospital, Amsterdam University Medical Centers, Vrije Universiteit Amsterdam and Amsterdam Neuroscience, Cellular & Molecular Mechanisms, 1105AZ Amsterdam, the Netherlands; [3]Department of Integrative Neurophysiology, Center for Neurogenomics and Cognitive Research, Vrije Universiteit Amsterdam, 1081HV Amsterdam, the Netherlands; [4]Andalusian Center for Developmental Biology (CABD), Pablo de Olavide University/CSIC/Junta de Andalucía, 41013 Seville, Spain; [5]Department of Molecular Biology and Biochemical Engineering, Pablo de Olavide University, 41013 Seville, Spain

Vanishing white matter (VWM) is a fatal leukodystrophy caused by recessive mutations in subunits of the eukaryotic translation initiation factor 2B. Currently, there are no effective therapies for VWM. Here, we assessed the potential of adenine base editing to correct human pathogenic VWM variants in mouse models. Using adeno-associated viral vectors, we delivered intein-split adenine base editors into the cerebral ventricles of newborn VWM mice, resulting in 45.9% ± 5.9% correction of the *Eif2b5*^{R191H} variant in the cortex. Treatment slightly increased mature astrocyte populations and partially recovered the integrated stress response (ISR) in female VWM animals. This led to notable improvements in bodyweight and grip strength in females; however, locomotor disabilities were not rescued. Further molecular analyses suggest that more precise editing (i.e., lower rates of bystander editing) as well as more efficient delivery of the base editors to deep brain regions and oligodendrocytes would have been required for a broader phenotypic rescue. Our study emphasizes the potential, but also identifies limitations, of current *in vivo* base-editing approaches for the treatment of VWM or other leukodystrophies.

INTRODUCTION

Vanishing white matter (VWM; Online Mendelian Inheritance of Men [OMIM] 603896) is a fatal neurodegenerative disease with unique molecular, pathological, and clinical features. VWM mostly presents in children, although it may also present at an earlier or later age,[1–4] with an inverse correlation between the onset age and clinical severity.[4–6] The disease is dominated by chronic motor decline (mostly progressive ataxia), less severe cognitive decline, and stress-induced episodes of rapid neurological deterioration.[5,7] Neuropathological analyses revealed several distinct white matter (WM) abnormalities in the brain, including (1) progressive degeneration of the WM with lack of myelin and astrogliosis; (2) an increase in pre-myelinating oligodendrocyte progenitor cells (OPCs) and immature, dysmorphic astrocytes; and (3) a lack of mature oligodendrocytes and astrocytes.[2,4,8–11]

Recessive pathogenic variants in the genes *EIF2B1-EIF2B5* encoding the five subunits (α–ε) of the eukaryotic translation initiation factor 2B (eIF2B) cause VWM.[12,13] eIF2B acts as a guanine nucleotide exchange factor (GEF) during translation initiation under physiological and stress conditions. During stress, eIF2B is the central factor in the integrated stress response (ISR) to downregulate the rate of protein synthesis. Pathogenic eIF2B variants decrease GEF activity and thereby constitutively activate the downstream ISR.[14,15] Even though eIF2B is ubiquitously expressed in all tissues, pathogenic variants almost exclusively affect the brain WM with dysfunctional astrocytes and impaired oligodendrocyte maturation being critical players in the pathophysiology.[5,8–11,16] In fact, previous work has shown that immature, dysfunctional astrocytes most likely drive VWM pathology via dysregulation of the ISR.[14,15]

Current treatments are symptomatic and focus on slowing down disease progression instead of targeting the underlying gene defect.[7] Recently developed CRISPR-Cas-based genome-editing technologies, such as base editors (BEs), open up promising strategies for treating genetic disorders, such as VWM, by directly correcting the pathogenic variant at the DNA level.[17,18] BEs consist of a cytidine- or an

Received 17 October 2023; accepted 5 March 2024;
https://doi.org/10.1016/j.ymthe.2024.03.009.

[6]Senior author
[7]These authors contributed equally
Correspondence: Niek P. van Til, Department of Child Neurology, Amsterdam Leukodystrophy Center, Emma Children's Hospital, Amsterdam University Medical Centers, Vrije Universiteit Amsterdam and Amsterdam Neuroscience, Cellular & Molecular Mechanisms, 1105AZ Amsterdam, the Netherlands.
E-mail: n.p.vantil@amsterdamumc.nl
Correspondence: Gerald Schwank, Institute of Pharmacology and Toxicology, University of Zurich, 8057 Zurich, Switzerland.
E-mail: schwank@pharma.uzh.ch

 © 2024 The Authors.
This is an open access article under the CC BY license (http://creativecommons.org/licenses/by/4.0/).

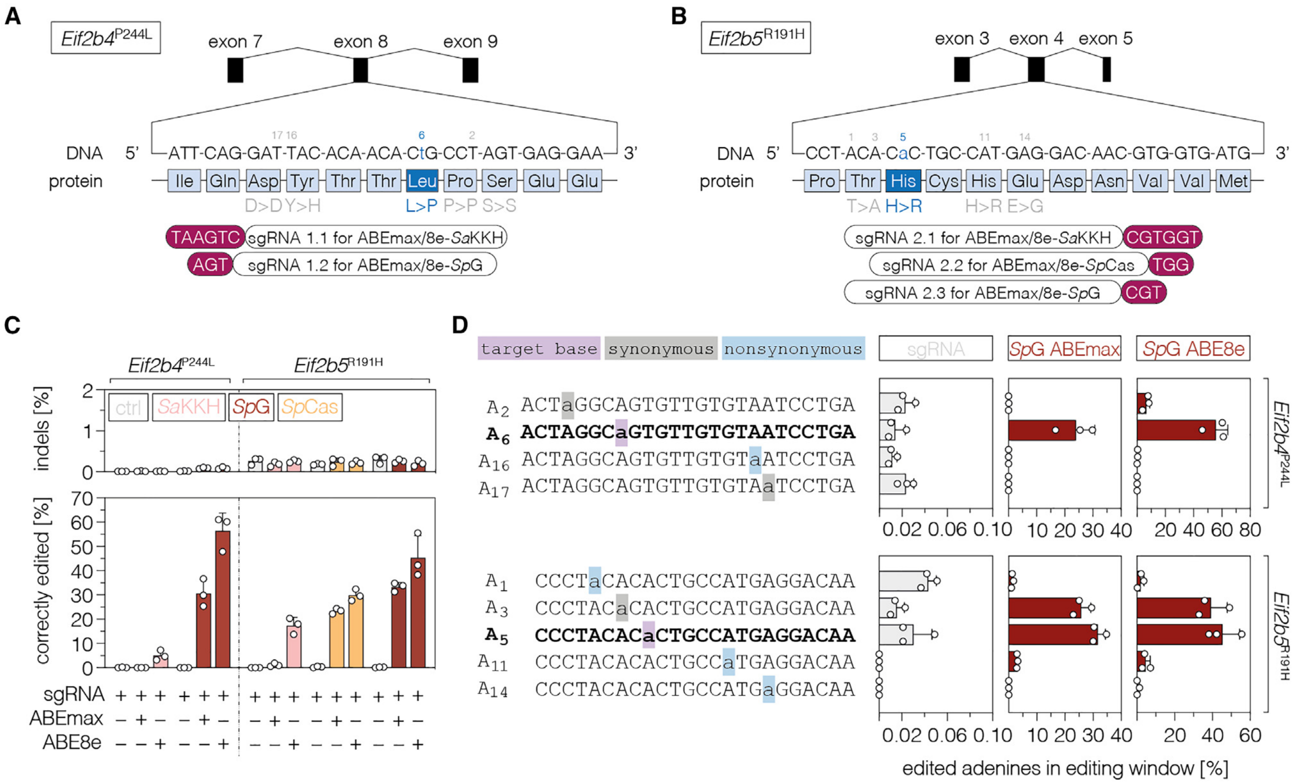

Figure 1. *In vitro* editing outcomes at the *Eif2b4* and *Eif2b5* locus

(A and B) Schematic representation of the *Eif2b4* (A) and *Eif2b5* (B) target sites and respective sgRNA designs for *Sp*Cas-, *Sp*G-, or *Sa*KKH-ABEs. (C and D) On-target editing (C), indel formation (C), and bystanders (D) at the *Eif2b4* (with sgRNA 1.2) and *Eif2b5* locus (with sgRNA 2.3) for ABEmax and ABE8e variants in HEK cell lines. Indels were quantified within a window covering both the deamination and nicking site. On-target editing (purple), synonymous bystanders (gray), and nonsynonymous bystanders (blue) are highlighted in the indicated colors. The desired editing outcome with precise on-target editing only is shown in bold. The numbering of adenines (A_x) is based on their position within the combined protospacer sequence of all used ABE variants. Control samples were transfected with sgRNA only. The used ABE variants are color coded (C) or indicated above the respective plot (D). Data are displayed as means ± SD of three independent experiments. See also Figure S1.

adenine deaminase covalently linked to a Cas9 nickase, allowing the conversion of C-G to T-A or A-T to G-C base pairs, respectively. Unlike classical Cas9 nucleases, they enable precise and efficient conversion of transition point mutations independent of homology-directed repair (HDR) and DNA double-strand breaks.[17–20] Hence, they are ideally suited for the application in post-mitotic tissues, such as the brain, which consist of cells with low HDR activity.

In this study, we assessed the therapeutic efficacy and potential side effects of adenine base editing in the brain of VWM mouse models. Adeno-associated virus (AAV) vector-mediated delivery of adenine BEs (ABEs) to neonatal mice enabled correction of two pathogenic VWM variants, with on average 29.8% ± 4.8% and 45.9% ± 5.9% editing in the cortex of *Eif2b4*^{P244L} and *Eif2b5*^{R191H} mice at experimental endpoints, respectively. Correction of the *Eif2b5*^{R191H} variant led to a mild increase in mature astrocyte populations and a notable decrease in the ISR in certain VWM-affected brain regions, leading to a significant increase in bodyweight and forelimb grip strength in female mice. Nonetheless, motor skills were not improved, likely due to low editing rates in deep brain regions, particularly the cerebellum, and oligodendrocytes. Our findings highlight the potential of base editing for VWM but also emphasize the need to further improve delivery methods as well as editing efficiency and precision.

RESULTS

In vitro correction of pathogenic VWM variants by adenine base editing

In recent years, numerous pathogenic variants have been identified that result in either early- or late-onset VWM in humans.[7,21–23] The two variants c.728C>T/p.243P>L in exon 8 of *EIF2B4* and c.584G>A/p.195R>H in exon 4 of *EIF2B5* lead to early onset and severe disease progression in humans[23,24] and are both transition point mutations that could in theory be corrected by A-to-G base editing. To identify ABE variants that correct these mutations with high efficiency and accuracy (low bystander editing activity), we first generated HEK cell lines where a 250-bp DNA sequence surrounding the corresponding pathogenic mouse variant was integrated into the genome using the PiggyBac transposon system.[25] Based on availability of the protospacer adjacent motif (PAM), we designed two single guide RNAs (sgRNAs) for *Eif2b4*^{P244L} (Figure 1A) and three sgRNAs for *Eif2b5*^{R191H} (Figure 1B). These

were co-delivered with *Sp*Cas-, *Sp*G-, or *Sa*KKH-ABE-expressing plasmids into HEK cell lines containing the corresponding VWM mouse variant. At the *Eif2b4* locus, deep sequencing revealed the highest correction efficiencies (55.8% ± 8.4%; Figure 1C) and low rates of nonsynonymous bystanders (0.3% ± 0.2%; Figures 1D and S1) when sgRNA1.2 was combined with *Sp*G-ABE8e. At the *Eif2b5* locus, the combination of *Sp*G-ABE8e and sgRNA2.3 was most efficient in correcting the pathogenic variant (45.3% ± 9.1%; Figure 1C); however, it also showed moderate levels of nonsynonymous bystanders (4.7% ± 2.3%; Figures 1D and S1). Notably, sgRNA2.3 also led to high correction rates (31.7% ± 2.7%; Figure 1C) with *Sp*G-ABEmax but led to slightly lower nonsynonymous bystanders (3.0% ± 0.2%; Figure 1C). Since this ABE variant also shows lower off-target deamination activity compared to ABE8e,[26] we decided to use sgRNA2.3 together with *Sp*G-ABEmax for the subsequent experiments at the *Eif2b5* locus.

In vivo correction of pathogenic variants via adenine base editing in VWM mouse models

While a VWM mouse model with the *Eif2b5* p.Arg191His variant (hereafter referred to as *Eif2b5*^{R191H}) has already been established and characterized,[11] a mouse model with *Eif2b4* p.Pro244Leu (hereafter referred to as *Eif2b4*^{P244L}) did not yet exist. Thus, we generated this model using CRISPR-Cas9-mediated HDR from a single-stranded DNA repair template in zygotes (Table S1; Figure S2). Neurological features in homozygous *Eif2b4*^{P244L} offspring were subsequently analyzed throughout development with respect to disease progression and pathology (Figures S3A–S3D). Heterozygous *Eif2b4*^{P244L} offspring were used as healthy controls. Unlike the *Eif2b5*^{R191H} mouse model,[11] these mice did not display any evident disease manifestations. The bodyweight was comparable to heterozygous littermates (Figure S3B) and none of the homozygous animals developed VWM neuropathologies such as overexpression of the GFAP delta isoform in WM astrocytes of the corpus callosum and myelin vacuolization in the cerebellar WM (Figure S3C).[10,11] In line with these results, astrocyte and oligodendrocyte maturation were not altered, as indicated by RT-qPCR at various time points (Figure S3D). Furthermore, the ISR key regulators *Gadd34*, *Atf4*, *Ddit3*, and *Trib3*, which are constitutively activated in VWM patients[14,15,27] and 2.5-fold upregulated in homozygous *Eif2b5*^{R191H} mice (Figure S3E; average fold change across all four transcripts), were only mildly upregulated in homozygous *Eif2b4*^{P244L} mice (on average, 1.6-fold increase compared to age-matched healthy controls; Figure S3E). While these results demonstrate that the *Eif2b4*^{P244L} model cannot be used to assess phenotypic VWM rescue upon treatment, we chose to use it alongside the well-characterized *Eif2b5*^{R191H} model to assess *in vivo* gene correction rates and potential side effects of *in vivo* adenine base editing.

Due to the low immunogenicity, rare genomic integration, and the ability to cross the blood-brain barrier and efficiently transduce astrocytes,[28,29] we decided to use AAV-PHP.eB[29] for *in vivo* delivery of our ABEs. To identify a delivery route that allows efficient AAV transduction in the brain of newborn C57BL/6J mice, we first packaged an EGFP expression vector under the control of the ubiquitously expressed Cbh promoter[30] into AAV-PHP.eB capsids and delivered particles to neonatal mice via intracerebroventricular (i.c.v.), retro-bulbar (RO), or temporal vein (TempV) injection (Figure S4A). Highest transduction efficiencies across various brain regions were achieved in i.c.v.-injected mice (Figures S4B and S4C), and, in line with previous reports,[29,31] we also observed efficient transduction of astrocytes (Figure S4C). Based on these findings, we opted to administer our base-editing treatment using the AAV-PHP.eB serotype through i.c.v. injection.

Since ABEmax and ABE8e exceed the packaging capacity of AAVs (∼5 kb including inverted terminal repeats [ITRs]),[32] we used the previously established *Npu* intein-mediated protein *trans*-splicing system to split the BE into two separate AAVs for *in vivo* expression.[33–35] We packaged intein-split *Sp*G-ABEmax (targeting variant *Eif2b5*^{R191H}; hereafter referred to as ABEmax) and *Sp*G-ABE8e (targeting variant *Eif2b4*^{P244L}; hereafter referred to as ABE8e) expression vectors, either under the control of the cytomegalovirus (CMV) (584 bp) or Cbh promoter (793 bp) in AAV-PHP.eB capsids (Figure 2A), and delivered particles to the ventricles of newborn mice at the maximum possible dosage (Figure 2B). Brains were isolated at 4 weeks post injection and editing outcomes were quantified in the cortex by deep sequencing (Figure 2C). Interestingly, we observed substantially higher correction rates at both loci with the Cbh promoter (*Eif2b4*^{P244L}, 31.6% ± 5.9%; *Eif2b5*^{R191H}, 28.6% ± 7.1%) compared to the CMV promoter (Figure 2C; *Eif2b4*^{P244L}, 7.1% ± 2.7%; *Eif2b5*^{R191H}, 3.3% ± 2.6%). While in *Eif2b5*^{R191H} animals this increase could be potentially attributed to delivering a 1.7-fold higher dose of the pCbh-ABEmax vector, *Eif2b4*^{P244L} animals received a 1.3-fold lower dose of the pCbh-ABE8e vector and still displayed higher editing efficiencies. In fact, despite administering a lower AAV dose, RT-qPCR revealed on average 2.6-fold higher ABE8e transcript levels (average of all four targeted sites) under the Cbh promoter in *Eif2b4*^{P244L} animals (Figure 2D), suggesting that stronger ABE expression under the Cbh promoter is the cause for improved editing rates in our experimental setup. Analysis of animals over a longer experimental time frame further revealed that editing rates were maintained in both mouse models (33 weeks for *Eif2b5*^{R191H} mice; 52 weeks for *Eif2b4*^{P244L} mice; Figures 3A, 3B, and S5) with ABE8e being still expressed in *Eif2b4*^{P244L} mice at week 52, albeit at substantially lower levels compared to earlier time points (Figure S5).

Base editing improved bodyweight and grip strength but not balance and motor skills in female *Eif2b5*^{R191H} mice

To determine whether adenine base editing reduces VWM severity, we examined characteristics of the murine VWM phenotype, including bodyweight and motor skills, in ABEmax- and saline-treated *Eif2b5*^{R191H} mice. ABEmax-treated female mice displayed an average 5.7% increase in bodyweight compared to saline-treated females at 6 weeks post treatment (16.2 ± 1.2 g vs. 15.3 ± 0.5 g), which continuously increased throughout development (on average, 16% bodyweight increase at experimental endpoints; Figure 4A). Notably, while ABEmax-treated males exhibited similar editing rates, they only showed an average 4.2% increase in bodyweight at endpoints (25.0 ± 1.7 g vs. 24.0 ± 1.9 g; Figures 4A and 4B). This prompted us to focus only on females for subsequent detailed phenotypic analyses. Alongside the increase in bodyweight, we observed a significant improvement of

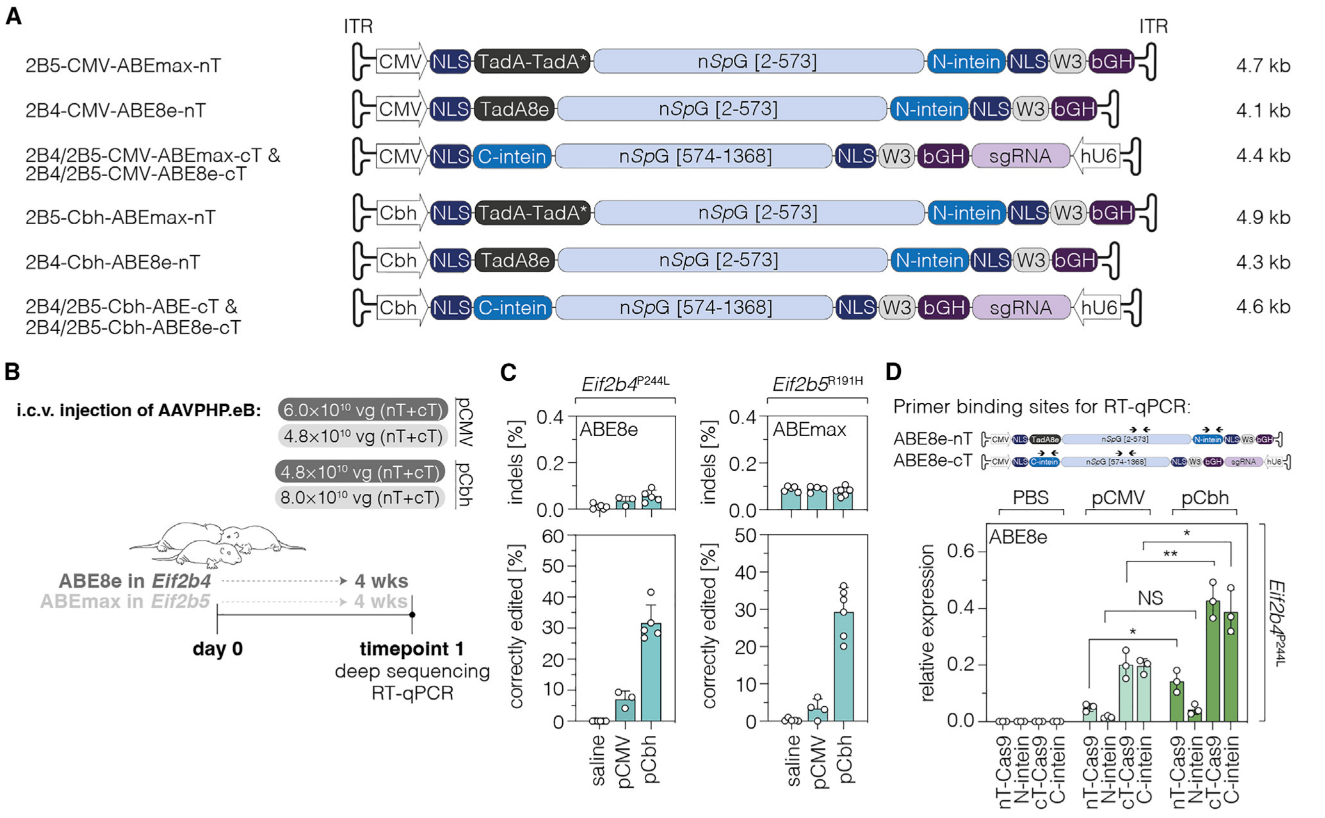

Figure 2. Comparative analysis of pCMV- and pCbh-ABEmax/ABE8e in the brain

(A) Schematic representation of AAV designs with the CMV or Cbh promoter used *in vivo* and their corresponding lengths in kilobasepairs (kb, including ITRs). Constructs are not depicted to scale. (B) Schematic representation of the experimental setup and timeline. Dark gray marks experiments with *Eif2b4*^{P244L} mice and light gray corresponds to experiments with *Eif2b5*^{R191H} mice. Maximum AAV doses and volumes were injected into newborn mice for each mouse model and vector. (C) On-target editing and indel rates at the *Eif2b4* (n = 3–6 mice per group) and *Eif2b5* locus (n = 4–7 mice per group) in mouse cortices at 4 weeks post injection. Indels were quantified within a window covering both the deamination and nicking site. (D) ABE8e transcript levels of N- and C-terminal AAV preparations in the striatum of *Eif2b4*^{P244L} mice (n = 3). Primer binding sites along the AAV constructs are indicated above the plot. Data are displayed as means ± SD of at least three mice per group and were analyzed using an unpaired Student's t test with Welch's correction (D). 2B4, *Eif2b4* locus; 2B5, *Eif2b5* locus; pCMV, human cytomegalovirus promoter; pCbh, truncated chimeric CMV/chicken β-actin hybrid promoter; nT, N-terminal AAV construct; cT, C-terminal AAV construct; NLS, nuclear localization signal; n*SpG*, *SpG* nickase; W3, woodchuck hepatitis virus post-transcriptional regulatory element; bGH, bovine growth hormone polyadenylation signal; hU6, human U6 promoter; vg, vector genomes; ITR, inverted terminal repeat. See also Figures S2–S4.

forelimb grip strength in ABEmax-treated homozygous *Eif2b5*^{R191H} females (1.05 ± 0.1 N vs. 0.83 ± 0.1 N; Figure 4C), which were almost comparable to healthy levels of heterozygous females (1.15 ± 0.2 N; Figure 4C). In contrast, motor control (Figure 4D), balance (Figure 4D), and ataxia-related behaviors (Figure 4E), including hindlimb clasping, gait, kyphosis, and ledge walking,[36,37] deteriorated in ABEmax-treated heterozygous and homozygous *Eif2b5*^{R191H} mice.

In summary, correction of the *Eif2b5*^{R191H} variant in female mice significantly improved bodyweight and restored forelimb grip strength. Meanwhile, balance and motor skills remained impaired or even declined.

Eif2b5^{R191H} correction rates were lower in deep brain regions and oligodendrocytes

In VWM, pathology arises from various WM regions of the brain and involves impaired maturation of astrocytes and oligodendrocytes.[8,11,14,38,39] It is therefore feasible that lower editing in particular brain regions or cell types hindered a wider phenotypic correction.

We therefore first assessed whether there were differences in base-editing rates across different brain regions. We manually dissected the brain into 10 distinct regions (olfactory bulb, cerebral cortex, corpus callosum, hippocampus, thalamus, striatum, midbrain, hypothalamus, hindbrain, and cerebellum; Figure S6) and analyzed correction rates at the *Eif2b5* locus at 8 weeks post treatment (Figure 5A; tissues isolated from female and male mice). In accordance with the biodistribution of EGFP expression after i.c.v. injection of AAV-PHP.eB particles (Figure S4), editing rates were higher in brain regions dorsal or rostral of the lateral ventricles (on average, 20.2% ± 10.7% for olfactory bulb, cerebral cortex, corpus callosum, hippocampus, and striatum; Figure 5A) compared to regions ventral or caudal of the lateral ventricles (on average, 12.7% ± 9.7% for thalamus, hypothalamus, midbrain, hindbrain, and cerebellum; Figure 5A). Editing also occurred in the spinal cord, heart, and liver

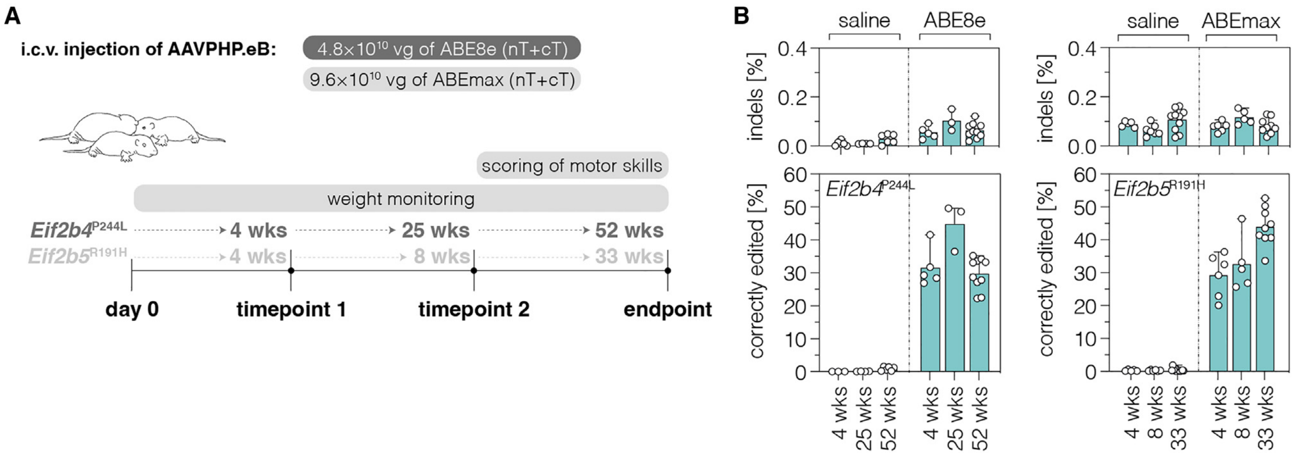

Figure 3. *In vivo* base editing at the *Eif2b4* and *Eif2b5* locus in the brain throughout development

(A) Schematic representation of the experimental setup and timeline. Dark gray marks experiments with the *Eif2b4*^{P244L} mouse model and light gray corresponds to experiments with the *Eif2b5*^{R191H} mouse model. Maximum AAV doses and volumes were injected for each mouse model and vector. (B) Editing and indel rates in the cortex at different time points at the *Eif2b4* locus (left; n = 3–10 cortices per group) and at the *Eif2b5* locus (right; n = 5–9 cortices per group). Experimental endpoints were selected based on life expectancy (*Eif2b5*^{R191H}) or potential occurrence of VWM phenotypes (*Eif2b4*^{P244L}) for the indicated model. Indels were quantified within a window covering both the deamination and nicking site. Data are displayed as means ± SD of at least three mice per group. 2B4, *Eif2b4* locus; 2B5, *Eif2b5* locus; vg, vector genomes. See also Figure S5.

(Figure S7), as anticipated based on the PHP.eB tropism and ubiquitous activity of the Cbh promoter.[30] Notably, no differences in brain editing efficiencies were observed between females and males (Figure S8A).

Next, we investigated whether local differences in editing rates between brain regions were also reflected by differences in VWM-associated ISR regulators. We extracted RNA from the cortex, corpus callosum, and cerebellum at endpoints (33 weeks) for analysis by RT-qPCR on key transcriptional ISR regulators.[27] Higher editing rates in the cortex (females, 45.9% ± 5.9%) and corpus callosum (females, 40.6% ± 5.2%) compared to the cerebellum (females, 3.5% ± 5.2%) were indeed reflected by a more pronounced reduction in mRNA levels of ISR regulators in these regions (*Atf4*, *Ddit3*, *Trib3*; Figure 5B), although, also in the cerebellum, a marked decrease in *Atf4* mRNA levels was observed (Figure 5B). Interestingly, males displayed similar editing rates in these three regions (cortex, 40.9% ± 0.5%; corpus callosum, 43.4% ± 4.1%; cerebellum, 1.8% ± 0.7%; Figure S8B) but did not display a consistent downregulation of the different ISR regulators (Figure S8C). Notably, mRNA levels of ISR regulators were comparable between females and males (Figure S8D).

We then performed histological analysis of saline- and ABEmax-treated *Eif2b5*^{R191H} mice. In line with the ABEmax biodistribution and the resulting differences in editing rates (Figures 5A and S8A), we observed a trend for decreased numbers of immature astrocytes (NESTIN/GFAP double-positive cells) in the anterior part of the corpus callosum (ABEmax-treated females, 1.4% ± 1.6%; saline-treated females, 2.7% ± 2.2%; Figures S9A and S9B), while mislocalization of Bergmann glia,[40] a specialized type of astrocytes located in the cerebellum, remained unchanged (ABEmax-treated females, 0.16% ± 0.1%; saline-treated females, 0.13% ± 0.05%; Figures S9C and S9D).

Next, we assessed whether we could detect differences in base-editing rates between astrocytes and oligodendrocytes. We purified both cell populations from whole brains using magnetic activated cell sorting (MACS; Figure S10) and analyzed cell-type-specific correction rates by deep sequencing. Importantly, we detected markedly higher editing in astrocytes (22.5% ± 6.1%) compared to oligodendrocytes (5.3% ± 3.1%; Figure 5C). In line with these results, fluorescence-activated cell sorting (FACS) analysis of cells isolated from the brain revealed a more pronounced, although not significant, increase in mature astrocyte populations in ABEmax-treated mice vs. saline-treated controls (56.8% ± 7.6% vs. 46.8% ± 3.5%; healthy controls, 61.9% ± 7.4%; Figure 5D) while oligodendrocyte populations were less affected (24.5% ± 5.4% vs. 27.7% ± 0.8%; healthy controls, 18.4% ± 3.9%; Figure 5D). Neuron and microglia/macrophage populations were altered neither by VWM nor by the ABEmax treatment (Figure 5D).

Finally, we employed high-resolution label-free mass spectrometry on whole brains to analyze the proteome of heterozygous saline-treated *Eif2b5*^{R191H} mice as well as ABEmax- and saline-treated homozygous *Eif2b5*^{R191H} mice. Global proteome analysis revealed a similar group of differentially expressed proteins between (1) ABEmax-treated homozygous mice and heterozygous controls as well as (2) saline-treated homozygous mice and heterozygous controls (Figure 5E), with an enrichment for the Gene Ontology terms catabolic/metabolic processes, amino acid metabolism, and translation (Figure S11). As these results suggest a limited effect of our ABEmax treatment on proteomic changes on a whole-tissue scale, we next analyzed changes in the expression of astrocyte- or oligodendrocyte-specific proteins (Figure 5F). In alignment with our flow cytometry analysis (Figure 5D), no pronounced changes in normalized protein abundances were detected for markers of oligodendrocyte maturation or myelination (Figure 5F, top), but in

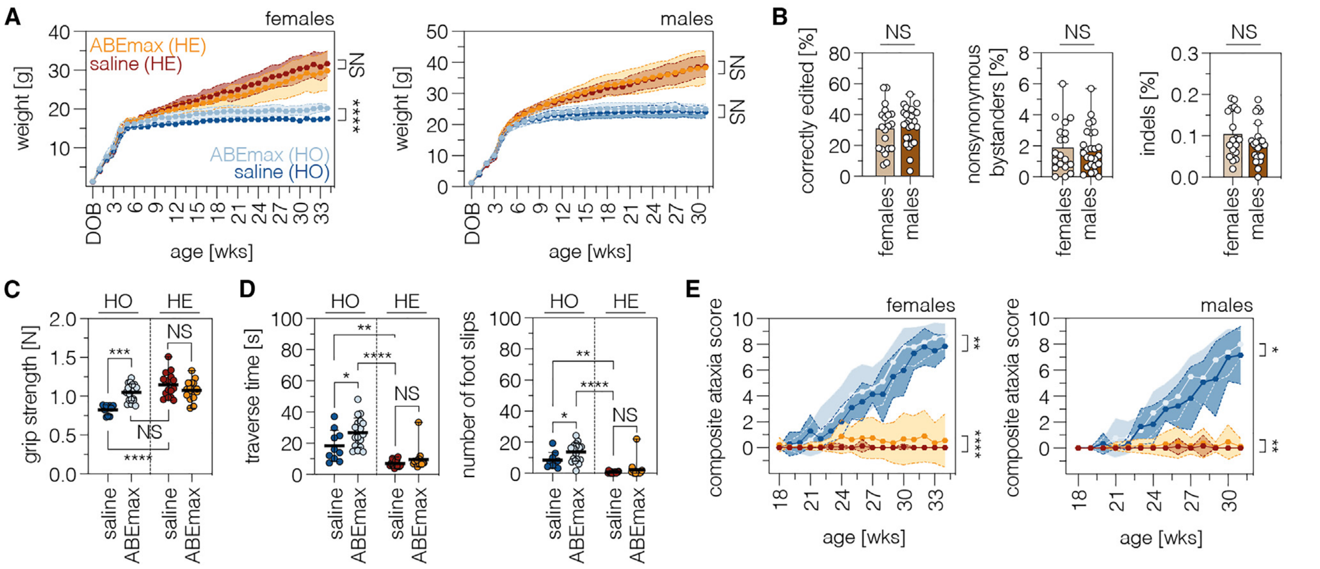

Figure 4. Base editing results in a partial phenotypic rescue in female *Eif2b5*^{R191H} mice

(A) Weekly bodyweight progression of female (homozygous/heterozygous, n = 12/19 mice for saline; n = 23/23 mice for ABEmax treatment) and male *Eif2b5*^{R191H} mice (homozygous/heterozygous, n = 12/26 mice for saline; n = 28/26 mice for ABEmax treatment). (B) Sex-specific on-target editing, nonsynonymous bystander editing, and indel rates at the *Eif2b5* locus (n = 20 homozygous females or 25 homozygous males). Indels were quantified within a window covering both the deamination and nicking site. (C–E) Forelimb grip strength (C; only female mice), balance and motor coordination in the balance beam test (D; only female mice), and composite ataxia scores in ABEmax- or saline-treated *Eif2b5*^{R191H} females (E; homozygous/heterozygous, n = 10/16 mice for saline; n = 19/17 mice for ABEmax treatment) and males (E; homozygous/heterozygous, n = 6/23 mice for saline; n = 27/25 mice for ABEmax treatment). Genotypes are indicated above each plot. Color coding of the groups in (C)–(E) is identical to (A). Data are displayed as means ± SD and were analyzed using a two-way ANOVA with Bonferroni's multiple comparisons test (A; ****p < 0.0001; NS, p > 0.05), an unpaired, two-tailed Student's t test with Welch's correction (B; NS > 0.05), a one-way ANOVA with Tukey's multiple comparisons test (C and D; *p < 0.05; **p < 0.005; ***p < 0.0005; ****p < 0.0001), and a repeated measures one-way ANOVA with Geisser greenhouse correction (E; *p < 0.05; **p < 0.005; ****p < 0.0001). NS, not significant; DOB, date of birth; N, Newton.

half of the ABEmax-treated mice (n = 3) we observed a slight trend for increased abundance of various markers for astrocyte maturation (subset 1 in Figure 5F). Notably, these three mice (subset 1) also displayed higher average editing (23.4% ± 15.5%) than the other 3 ABEmax-treated mice (13.7% ± 4.6%; subset 2 in Figure 5F).

In summary, our data suggest that lower editing in ventral/caudal brain regions vs. dorsal/rostral brain regions, as well as in oligodendrocytes vs. astrocytes, contributed to the incomplete rescue of VWM phenotypes.

Base editing induced potentially harmful bystander mutations at the *Eif2b5* locus

Base editing can cause the introduction of nonsynonymous bystander edits or indels at the targeted locus or off-target editing at other sites in the genome. These unintended mutations could critically influence the functionality of affected cells or even trigger oncogenic transformations. In fact, aggravation of motor skills in heterozygous and homozygous *Eif2b5*^{R191H} mice has recently been reported in a study where the delivery of an *Sa*Cas9 nuclease in combination with an HDR template led to ∼5% of undesired indel mutations at the locus.[37]

First, we analyzed sequencing reads in ABEmax-treated *Eif2b5*^{R191H} animals for undesired mutations at the targeted locus. Measuring indel rates, we found that they were only marginally increased in ABEmax-treated animals vs. saline-treated controls (0.09% ± 0.04% vs. 0.05% ± 0.01%; in whole brain tissues; 0.08% ± 0.03% vs. 0.04% ± 0.02% in astrocytes; 0.09% ± 0.03% vs. 0.03% ± 0.03% in oligodendrocytes; Figure S12). However, when we next analyzed synonymous bystander mutations, we observed, on average, 4.8% editing in homozygous and heterozygous mice, respectively (Figure 6A and 6B). We then correlated bystander editing rates with phenotypic measurements and found that, although grip strength and balance performance were not correlated with bystander editing (Figure 6C; grip strength, r = 0.06, p = 0.79; traverse time, r = 0.34, p = 0.14), weight loss became more apparent with increasing bystander editing in heterozygous *Eif2b5*^{R191H} animals (Figure 6C; Pearson correlation coefficient r = 0.54, p = 0.01). Since bystander mutations can also be introduced on the wildtype allele (A_3, 4.6% ± 0.6%; A_{11}, 0.5% ± 0.2%; Figure S13), we speculate that the sporadic generation of cells where one allele carries the *Eif2b5*^{R191H} mutation and the other the bystander mutation could have led to the deterioration of VWM phenotypes in heterozygous animals, similar to VWM patients carrying compound heterozygous mutations.[41,42] Furthermore, it is feasible that the bystander mutation leads to a more severe reduction in *Eif2b5* function, potentially explaining the negative effect of the treatment on motor skills in homozygous animals.

Next, we assessed whether ABE treatment led to guide-dependent off-target editing at other regions in the genome. We used CHANGE-seq[43] (circularization for high-throughput analysis of nuclease genome-wide

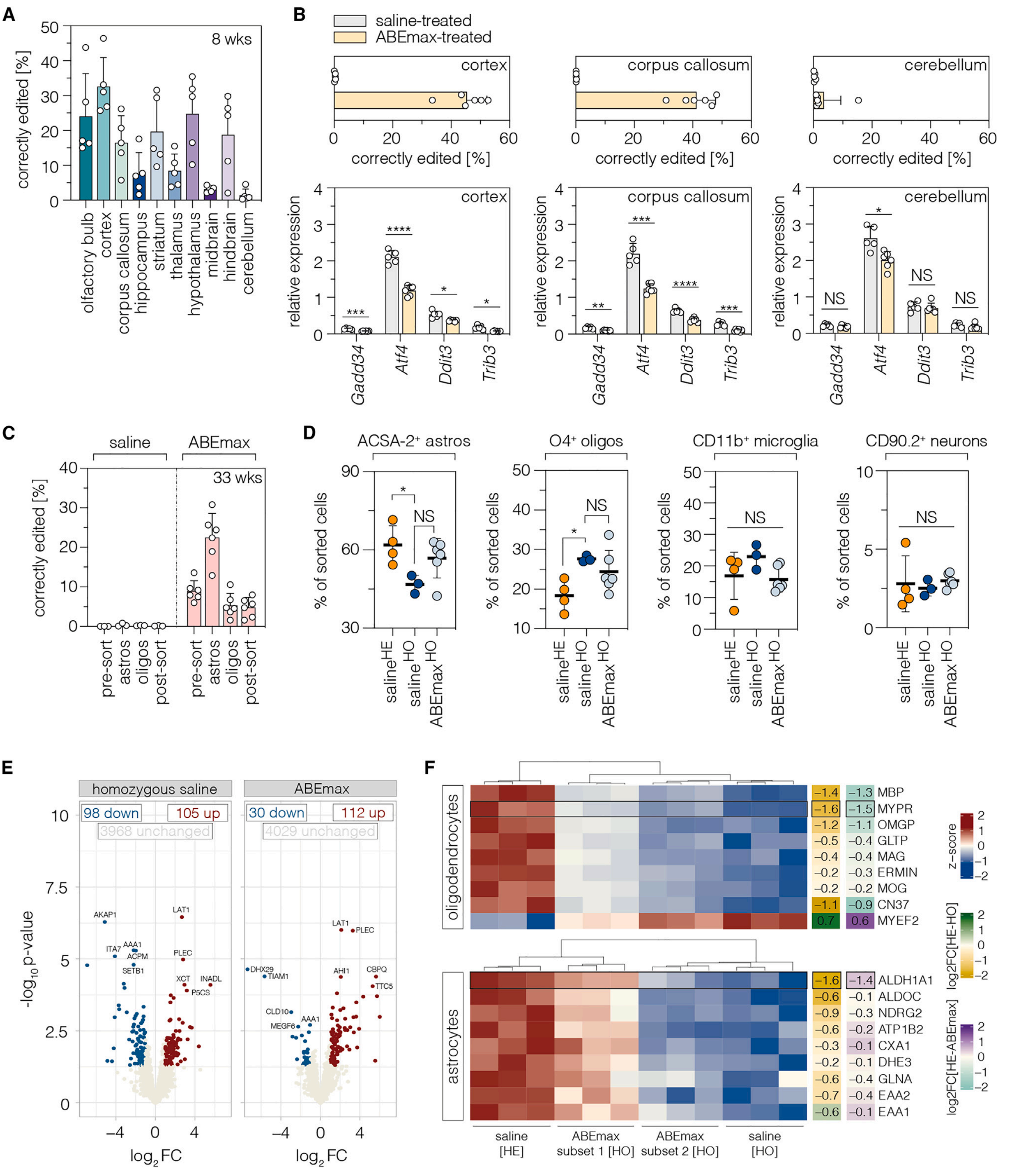

Figure 5. Base editing across different brain regions and cell populations

(A) Editing rates at the *Eif2b5* locus in different brain regions at 8 weeks post treatment (n = 3 males; n = 2 females). (B) Endpoint editing rates and corresponding transcript levels of ISR regulators in the cortex, corpus callosum, and cerebellum of saline- and ABEmax-treated *Eif2b5*^{R191H} mice (n = 5–6 mice per group). Transcripts were normalized to *Akt*. All mice were females except for three male mice in the saline-treated group. (C) Editing rates in cells before magnetic sorting (pre-sort), after magnetic

(legend continued on next page)

effects by sequencing) to experimentally identify potential off-target binding sites of the *Eif2b4* and *Eif2b5* sgRNAs (Figures S14A and S14B), and we then analyzed the top five identified off-target sites by deep sequencing in ABEmax/ABE8e-treated animals. Importantly, we identified editing rates above levels of saline-treated controls at off-target site 4 (dedicator of cytokinesis protein 5 [*Dock5*]) in *Eif2b4*^{P244L} mice and off-target site 1 (Sec11 homolog A [*Sec11a*]) in *Eif2b5*^{R191H} mice (Figures S14A and S14B). Dock5 is a GEF for Rho family GTPases, which is involved in the regulation of cytoskeleton dynamics.[44] Sec11a belongs to the signal peptidase complex, which is crucial for the secretion of a variety of secretory proteins in the ER-Golgi secretion pathway.[45] While both genes are expressed in neural populations,[46] the sgRNA off-target sites are located in intronic regions. Hence, it is unlikely that editing at these sites led to a phenotypic effect. In line with this assumption, RT-qPCR indicated no differences in *Dock5* or *Sec11a* transcript levels between saline- and ABEmax/ABE8e-treated animals (Figures S14C and S14D).

Finally, we assessed whether expression of the ABE itself could have also affected the VWM phenotype. We therefore delivered AAV-PHP.eB particles, expressing either EGFP or ABEmax under the Cbh promoter, at a dose of 8.0×10^{10} vg per animal via i.c.v. injection and analyzed ISR regulators after 5 weeks. RT-qPCR indicated no upregulation of *Gadd34*, *Atf4*, *Ddit3*, and *Trib3* within this experimental time frame (Figure S15A). Additionally, we did not detect an upregulation of the ISR (Figure S15B) or obvious signs of tissue necrosis (Figure S16) in the non-phenotypic *Eif2b4*^{P244L} model after treatment.

In summary, our data suggest that induction of bystander mutations at the *Eif2b5* locus likely had a negative effect on VWM symptoms, leading to the aggravation of motor skills in treated mice. The contribution of other factors, nevertheless, cannot be excluded.

DISCUSSION

In our study, we applied adenine base editing to correct two pathogenic VWM variants in the murine brain. Delivery of AAV vectors encoding intein-split ABEmax into the neonatal *Eif2b5*^{R191H} mice resulted in editing efficiencies of 45.9% ± 5.9% in the cortex. Using a comprehensive approach of deep sequencing, RT-qPCR, mass spectrometry, flow cytometry, histology, and behavioral assays, we demonstrated that *Eif2b5*^{R191H} correction efficiencies were (1) sufficient in certain VWM-susceptible brain regions to downregulate the ISR locally, (2) sufficient in astrocytes to partly restore eIF2B activity and slightly increase mature astrocyte counts in dorsal regions such as the corpus callosum, and (3) sufficient to significantly improve bodyweight and restore forelimb grip strength, but they were (1) insufficient in immature oligodendrocytes to induce maturation and re-myelination, and (2) insufficient to mitigate ataxia.

Our study overcomes several pitfalls of previously published work where an *Sa*Cas9 nuclease and HDR template were employed to correct the *Eif2b5*^{R191H} mutation in VWM neonates.[37] In the respective study, the vast majority of CRISPR-Cas9-induced edits at the *Eif2b5* locus were undesired indel mutations (4.64% ± 1.7%, n = 51) and imprecise corrections (0.27% ± 0.17%, n = 51). This resulted in frequent premature death of treated heterozygous and homozygous mice with no phenotypic improvements in the surviving animals. In sharp contrast, our study yielded precise and efficient correction of the pathogenic variant (cortex, 45.9% ± 5.9%; Figure 5B), significant improvement of bodyweight and grip strength in treated *Eif2b5*^{R191H} females (Figure 4), and no premature death in any of the treated heterozygous and homozygous animals.

We speculate that two major points would have to be addressed for a complete phenotypic rescue of VWM. First, a better biodistribution of the genome editor would have to be achieved, as neither i.c.v., RO, nor TempV delivery of AAV-PHP.eB particles enabled efficient transduction of the cerebellum (Figure S4C).[31] Additionally, the serotype PHP.eB has been reported to efficiently target neurons and astrocytes but less efficiently transduce oligodendrocytes.[29] Alternative routes of administration, as well as AAV capsids with an enhanced tropism for both astrocytes and oligodendrocytes, such as the recently developed Olig001 serotype,[47] should therefore be evaluated. Second, higher accuracy in gene correction would be required, as moderate levels of nonsynonymous bystander edits (on average, 4.8%) were likely triggering motor function decline in treated homozygous and heterozygous *Eif2b5*^{R191H} mice in our study. In the future, gene editing tools with increased precision, such as BEs with narrower editing windows or prime editors,[48] should be tested.

In conclusion, our study underlines the therapeutic potential of base editing to correct genetic diseases in the brain but also highlights

enrichment of astrocytes (astros) or oligodendrocytes (oligos), and the remaining cells after sorting of astros and oligos (post-sort; n = 3 or 6 mice per group). The purity of all cell populations was confirmed by flow cytometry (Figure S8). All mice were females except for one male mouse in the saline-treated heterozygous group. (D) Population size of astrocytes, oligodendrocytes, microglia/macrophages, and neurons quantified by flow cytometry (n = 3–6 female mice per group). Only female mice were used. Poorly perfused brains were excluded from the analysis. (E) Volcano plots showing differentially expressed proteins in saline- (n = 3 female mice) or ABEmax-treated *Eif2b5*^{R191H} animals (n = 6 female mice; cutoff: log_2 fold change of at least ±1.5 and adjusted p value < 0.05). Both groups were compared to heterozygous *Eif2b5*^{R191H} saline-treated controls (n = 3 female mice). Treatment groups are indicated at the top of each plot. The number of unchanged, upregulated, and downregulated proteins is indicated. The top five up- and downregulated proteins are labeled. Only female mice were used for mass spectrometry. (F) Heatmaps and hierarchical clustering of normalized protein abundances of oligodendrocyte- (top) or astrocyte-specific (bottom) proteins in saline- (n = 3 female mice) and ABEmax-treated *Eif2b5* animals (n = 6 female mice). Heatmaps of log_2 fold changes (saline-treated heterozygous compared to saline-treated homozygous [HE-HO] or ABEmax-treated homozygous [HE-ABEmax]) are indicated on the right side of each plot. Proteins that were significantly up- or downregulated in one condition are framed in black. ABEmax-treated animals were compared to saline-treated heterozygous (HE; healthy) or saline-treated homozygous controls (HO; diseased). Data are displayed as means ± SD of at least three animals per group and were analyzed using an unpaired two-tailed Student's t test (B; *p < 0.05; **p < 0.005; ***p < 0.005; ****p < 0.001) or a one-way ANOVA with Dunn's multiple comparisons test (D; NS, p > 0.05; *p < 0.05). WM, white matter; FC, fold change; up, upregulated; down, downregulated; astros, astrocytes; oligos, oligodendrocytes; ACSA-2, astrocyte cell surface antigen 2; O4, oligodendrocyte marker O4; sal, saline-treated. See also Figures S6–S11.

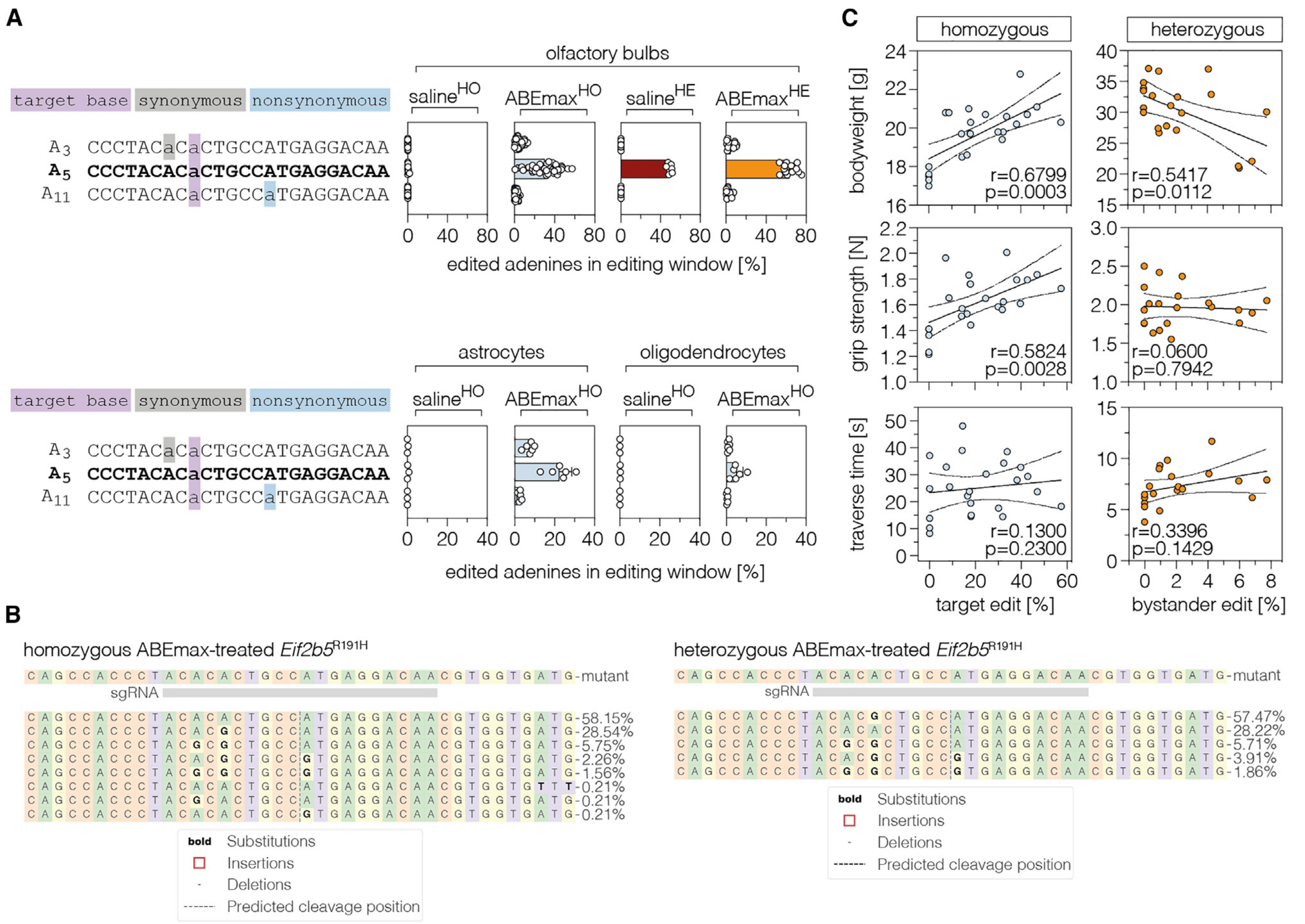

Figure 6. Nonsynonymous bystander edits might contribute to a deterioration of VWM phenotypes in *Eif2b5*^{R191H} mice

(A) Bystander edits at the *Eif2b5* locus in olfactory bulbs (top; n = 7 or 40 mice per group) or purified astrocytes and oligodendrocytes (bottom; n = 3 or 6 mice per group) of saline- or ABEmax-treated mice. The treatments and genotypes are indicated above each plot. On-target editing (purple), synonymous bystanders (gray), and nonsynonymous bystanders (blue) are highlighted in the indicated colors. The desired editing outcome, with precise on-target editing only, is shown in bold. Control mice were injected with saline. The numbering of adenines (A_x) is based on their position within the combined protospacer sequence as shown in Figure 1D. (B) Representative CRISPResso output files showing editing outcomes in heterozygous and homozygous ABEmax-treated *Eif2b5*^{R191H} animals. Editing outcomes with a frequency below 0.20% are not displayed. (C) Pearson correlations of base-editing outcomes and endpoint bodyweight (top), forelimb grip strength (middle), and traverse time across a narrow beam (bottom). Pearson correlation coefficients, 95% confidence intervals, and p values are indicated in the respective plot (n = 24 female mice). Data are displayed as means ± SD of at least three mice per group (A). Each data point represents one animal. See also Figures S12–S16.

current limitations that need to be addressed to move toward clinical application.

MATERIALS AND METHODS

Generation of plasmids

sgRNA plasmids were generated by ligating annealed and phosphorylated oligos into a BsmBI-digested lentiGuide-Puro (Addgene #52963) using T4 DNA ligase (NEB). To generate the PiggyBac reporter plasmid for the *Eif2b4* and *Eif2b5* locus, inserts with homology overhangs for cloning were ordered from Integrated DNA Technologies (IDT) and cloned into a XbaI- and EcoRI-digested pPB-Zeocin backbone using HiFi DNA Assembly Master Mix (NEB). To prepare plasmids for AAV production, inserts with homology overhangs were either ordered as gBlocks (IDT) or generated by PCR. Inserts were cloned into AgeI- and NotI-digested AAV backbones (Addgene #137177 and #137178) using HiFi DNA Assembly Master Mix (NEB). All PCRs were performed using Q5 High-Fidelity DNA Polymerase (NEB). The identity of all plasmids was confirmed by Sanger sequencing. Primers used for cloning of all plasmids are listed in the supplemental methods (Tables S4 and S5). Cbh_v5 AAV-ABE N-terminal (Addgene #137177) and Cbh_v5 AAV-ABE C-terminal (Addgene #137178) were gifts from D. Liu. lentiGuide-Puro was a gift from F. Zhang (Addgene #52963).

Cell culture transfection and genomic DNA preparation

HEK293T (American Type Culture Collection [ATCC] CRL-3216) cells and Hepa1–6 (ATCC CRL-1830) were maintained in Dulbecco's modified Eagle's medium (DMEM) plus GlutaMAX (Thermo Fisher

Scientific) supplemented with 10% (v/v) fetal bovine serum (FBS) and 1% penicillin/streptomycin (Thermo Fisher Scientific) at 37°C and 5% CO_2. Cells were passaged every 3 to 4 days and kept at a confluency below 90%. Cells were seeded in 96-well cell culture plates (Greiner) and transfected at 70% confluency using 0.5 μL of Lipofectamine 2000 (Thermo Fisher Scientific). If not stated otherwise, 300 ng of BE and 100 ng of sgRNA were used for transfections. Cells were incubated for 3 days after transfection and genomic DNA was isolated at 60°C using a lysis buffer as previously described.[49]

Genomic DNA from mouse tissues was isolated using phenol/chloroform extraction (brain regions at all time points and organs at endpoints of *Eif2b4*^{P244L} mice, olfactory bulbs and organs at endpoints of *Eif2b5*^{R191H} mice), using the AllPrep DNA/RNA/protein mini kit (Qiagen, brain regions of *Eif2b*^{R191H} *5* mice at 4 weeks post injection), using the 96 AllPrep DNA/RNA kit (Qiagen, brain regions of *Eif2b5*^{R191H} mice at 8 weeks post injection) or using sequential DNA/RNA isolation based on TRIzol reagent and chloroform (Thermo Fisher Scientific, brain regions of *Eif2b5*^{R191H} mice at endpoints). Briefly, 1 mL of TRIzol was added to 0.2 mL of tissue lysates and samples were subsequently mixed by inverting the tubes. Subsequently, 0.2 mL of chloroform was added and samples were shaken vigorously. After a 3-min incubation at room temperature, samples were centrifuged for 20 min at 12,000 × *g* and 4°C. The aqueous phase was collected and transferred into a fresh Eppendorf tube for later RNA extraction (see section "RNA isolation and RT-qPCR"). Extraction buffer (4 M guanidine thiocyanate, 50 mM sodium citrate, 1 M Tris) was added to the interphase-organic phase mixture (500 μL of buffer per 1 mL of TRIzol) and samples were vigorously mixed for 15 s. Samples were incubated for 10 min at room temperature and centrifuged for 15 min at 12,000 × *g* and 4°C to separate the phases. The upper, aqueous phase was transferred to a clean tube and isopropanol (0.4 mL per 1 mL of TRIzol) was added to precipitate DNA. Samples were mixed, incubated for 5 min at room temperature, and centrifuged for 5 min at 12,000 × *g* and 4°C. DNA pellets were washed with 1 mL of 75% ethanol and final pellets were resuspended in 8 mM NaOH. For long-term storage at −20°C, the pH was adjusted to 7 with HEPES (Sigma-Aldrich).

Generation of VWM reporter cell lines

To generate a dual *Eif2b4* and *Eif2b5* reporter cell line with the PiggyBac transposon,[25] HEK293T cells were seeded into a 48-well cell culture plate (Greiner) and transfected at 70% confluency with 225 ng of the PiggyBac transposon and 25 ng of the transposase using Lipofectamine 2000 (Thermo Fisher Scientific) according to the manufacturer's instructions. Three days after transfection, cells were enriched for 10 days using Zeocin selection (150 μg/mL).

AAV production

Single-stranded AAV2-based vector with capsid PHP.eB was produced by the Viral Vector Facility of the Neuroscience Center Zurich. Briefly, AAV vectors were ultracentrifuged and diafiltered. Physical titers (vector genomes per milliliter [vg/mL]) were determined using a Qubit 3.0 fluorometer (Thermo Fisher Scientific) as previously published.[50] The identity of the packaged genomes of each AAV vector was confirmed by Sanger sequencing.

Animal studies

Animal experiments were performed in accordance with protocols approved by the Kantonales Veterinäramt Zürich and the Animal Approval Committee of the Vrije Universiteit Amsterdam and were in compliance with all relevant ethical regulations. C57BL/6J and *Eif2b4*^{P244L} mice were housed in a pathogen-free animal facility at the Institute of Pharmacology and Toxicology of the University of Zurich. Mice were kept in a temperature- and humidity-controlled room on a 12-h light-dark cycle. Mice were fed a standard laboratory chow (Kliba Nafag no. 3437 with 18.5% crude protein). *Eif2b5*^{R191H} mice were weaned at P21 and kept at a 12-h light-dark cycle with food and water provided *ad libitum*. Unless indicated otherwise, animals were sacrificed at the indicated different ages as planned for the study or when they lost either ≥15% of their bodyweight in a few days or ≥20% compared to the highest measured weight. For analysis of transduction efficiencies, newborn mice (P1) received 4.0×10^{10} vg per animal via i.c.v. injection, 3.0×10^{11} vg per animal via RO injection, and 3.0×10^{11} vg per animal via TempV injection. For ABEmax/ABE8e treatments, *Eif2b4*^{P244L} newborn mice (P1) received 6.0×10^{10} vg (pCMV-ABE8e) or 4.8×10^{10} vg (pCbh-ABE8e) per animal via i.c.v. injection unless stated otherwise. *Eif2b5*^{R191H} newborn mice (P1) received 4.8×10^{10} vg (pCMV-ABEmax), 8.0×10^{10} vg (pCbh-ABE8e), or 9.6×10^{10} vg (pCbh-ABE8e) per animal via i.c.v. injection unless stated otherwise. Starting from birth, bodyweight of *Eif2b5*^{R191H} mice was monitored weekly until experimental endpoints (4, 8, or 33 weeks). Endpoints were selected based on VWM severity and life expectancy of the *Eif2b5*^{R191H} model. Grip strength and balance beam test were measured 8 months post injection. Composite ataxia scores were recorded from 4 months post injection until experimental endpoints (33 weeks).

Generation of *Eif2b4*^{P244L} knockin mouse model

Two sgRNAs with high on-target specificity and low off-target activity were designed using algorithms from CRISPOR (https://crispor.tefor.net/), IDT, and Benchling (https://www.benchling.com/; Table S1) and ordered as synthetic sgRNAs (IDT). To test the activity of both synthetic sgRNAs *in vitro*, we first amplified the region around the *Eif2b4* target site via PCR using oligos 19017.P1 and 19017.P4 (supplemental methods; Table S6). Next, we complexed the synthetic sgRNAs with the *Sp*Cas9-HF protein (IDT), incubated the RNP complex with the PCR fragment for 2 h at 37°C, and confirmed cleavage by visualizing cutting activity on an agarose gel (Figure S2A). The repair template (~400 bases in length with 200 bases of homology arms on each side of the mutation) was designed to include silent mutations (c.732T>G and c.735C>T) in the PAM and seed region of the sgRNA to abolish re-cutting of the targeted allele after recombination (Figure S2B) and was delivered as a single-stranded DNA oligo (IDT).

C57BL/6J-derived oocytes were co-injected with RNP complex (sgRNA_m01) and the single-stranded DNA repair template. Two rounds of pronuclear injections were performed (Figure S2C). In trial

1, a total of 226 oocytes were injected and 218 embryos were transferred into seven foster mothers, of which five became pregnant. One small litter was received, but none of the pups survived. In trial two, a total of 213 oocytes were injected and 122 embryos were transferred into six foster mothers, of which four became pregnant. One pup was born and the *Eif2b4* mutation site was analyzed by PCR. Using oligos 19017.P1 and 19017.P4, we first confirmed correct integration at the target site. Next, we amplified the region around the targeted exon using oligos 19017.P5 and 19017.P6 and confirmed the presence of a homozygous p.244Pro>Leu point mutation by Sanger sequencing (Figure S2D). The founder mouse was subsequently bred with C57BL/6J females to expand the colony.

Neonatal injections

The i.c.v. injections were performed as previously described.[37] P1 pups were cryoanesthetized on a paper towel on ice for 7 min and transferred to an ice-cold clay mold. AAV particles or saline were injected into both hemispheres at 1 μL/min, 2 μL per lateral ventricle, using a 10-μL Nanofil syringe with a 34G beveled Nanofil needle controlled by a Micro4 microsyringe pump in a stereotaxic frame (World Precision Instruments). Injection coordinates were X = ±0.8, Y = ±1.5, Z = from −1.7 to −1.5 mm from lambda. The needle remained in place in the injection site for 1 min before being slowly removed over a course of 30 s. The pups were placed on a thermoregulated heat mat at 37°C for 10 min to warm up before being placed back in the cage with the mother. Administration of constructs was not randomized across litters.

RO and TempV injections were performed as previously described.[51,52] P1 pups were cryoanesthetized on a paper towel on ice for 7 min. For RO and TempV injection, 30 μL of AAV particles at the maximum possible dose were slowly injected and the needle was kept in place at the injection site for 10 s before slowly being removed over the course of 3 s. The pups were placed on a thermoregulated heat mat at 37°C for 10 min to warm up before being placed back in the home cage with the mother.

Behavior assays

Neurological deterioration was assessed weekly by a composite scoring system for cerebellar ataxia as previously described.[36] Mice were scored on a scale of 0–3 for four different aspects: hindlimb clasping, gait, kyphosis, and ledge walking. In contrast to Guyenet et al.,[36] pelvic tilt was included as an additional parameter and scored for presence (1) or absence (0), resulting in a final combined score between 0 and 13. A gradual increase in score indicates an increase in neurological deterioration and, thus, deterioration of the VWM phenotype.

Motor skills of female mice were first assessed on a 1-m-long, 1.2-cm-wide balance beam, after training on a 2.6-cm-wide beam.[11] The last out of three runs was used for data analysis. Next, we assessed grip strength using a grip strength meter (Columbus Instruments). Briefly, the mouse was placed on the raster of the grip strength meter and slowly pulled back by its tail. Grip strength data is depicted as an average of five repetitions.

Trans-cardiac perfusion, organ/brain isolation, and dissection of brain regions

For C57BL/6J and *Eif2b4*^{P244L} mice, sodium pentobarbital (Kantonsapotheke Zürich) was injected via intraperitoneal injection at a dose of 100 mg/kg. Complete anesthesia was confirmed by the absence of a toe pinch reflex. Mice were placed on the perfusion stage inside a collection pan and the peritoneal cavity was exposed. The diaphragm was cut through laterally and the rib cage was cut parallel to the lungs, creating a chest flap. The flap was clamped in place using a hemostat (Fine Science Tools) and a 25G needle (Sterican), attached to silicon tubing and a peristaltic pump, was inserted into the left ventricle. The right atrium was cut for drainage. Unless stated otherwise, animals were perfused with ice-cold PBS (Thermo Fisher Scientific) at a rate of 10 mL/min. For *Eif2b5*^{R191H} mice, avertin (2,2,2-tribromoethanol in tertiary amyl alcohol, Sigma-Aldrich) was injected via intraperitoneal injection at a dose of ±24 μL/g bodyweight. Once no pedal withdrawal reflex was observed, animals were transcardially perfused with ice-cold PBS (Thermo Fisher Scientific). Once the perfusion was complete, mice were decapitated and the skull was removed with scissors and tweezers without inflicting damage to the underlying tissue. The brain was removed using a spatula. Brain regions (olfactory bulb, cortex, corpus callosum, hippocampus, striatum, thalamus, hypothalamus, midbrain, hindbrain, and cerebellum) were either dissected on ice directly after removal from the skull[53] or dissected from snap-frozen hemispheres.[54]

For DNA, RNA, and protein isolations, whole brains (protein) or brain regions (DNA and RNA) were either used directly or snap-frozen for later processing (see sections "cell culture transfection and genomic DNA preparation," "RNA isolation and RT-qPCR," and "protein isolation and mass spectrometry"). For single-cell isolation, whole brains were processed as described in section "single-cell isolation by MACS." Organs (other than brain) from both models were isolated at endpoints and either directly processed or snap-frozen for later DNA isolation (see section "cell culture transfection and genomic DNA preparation").

For histology of C57BL/6J and *Eif2b4*^{P244L} mice, animals were perfused with ice-cold fixative (4% paraformaldehyde [PFA], Sigma-Aldrich) and brains were post-fixed in 4% PFA for 4 h, followed by overnight incubation in 30% sucrose (w/v in PBS, Thermo Fisher Scientific). For histology of *Eif2b*^{R191H} mice, PBS-perfused brains were post-fixed in 4% PFA (Electron Microscopy Sciences) for 24 h. The cerebellum and brainstem were subsequently dissected and incubated in 70% ethanol for at least 7 h, while the rest of the brain was incubated in 30% sucrose (w/v in PBS, Sigma-Aldrich) at 4°C and allowed to sink to the bottom of the solution (1–2 days). Data were analyzed by researchers blinded to treatment and genotype.

RNA isolation and RT-qPCR

For *Eif2b4*^{P244L} mice, RNA was isolated from snap-frozen brain tissues using the RNeasy Lipid Tissue Mini Kit (Qiagen) according to the manufacturer's instructions. For *Eif2b5*^{R191H} mice, RNA was isolated from snap-frozen brain tissues using TRIzol and chloroform (Thermo Fisher Scientific). After adding TRIzol and chloroform to

tissue lysates, the aqueous phase was collected and added to linear acryl amide (1/250 of the TRIzol volume). Samples were mixed by swerving and chilled isopropanol (Sigma-Aldrich, half of the TRIzol volume) was added. Samples were mixed, incubated for 10 min at room temperature, and centrifuged for 20 min at 20,000 × *g* and 4°C. Pellets were washed with 0.25 mL of ice-cold 70% ethanol and centrifuged for 5 min at 12,000 × *g* and 4°C. After removing all of the ethanol, RNA pellets were air-dried for 1 min at room temperature. Following addition of 20 μL of H_2O to the pellets, samples were frozen overnight. Next, samples were thawed on ice and sodium acetate (1:10 diluted in H_2O) and 96% ethanol (2.5× concentrated) was added. After 30-min incubation at −20°C, samples were centrifuged for 30 min at 12,000 × *g* and 4°C. Next, samples were washed twice with 70% ethanol. Last, samples were resuspended in 20 μL of RNase-free H_2O and incubated overnight at −20°C. RNA was subsequently reverse transcribed to cDNA using random primers and the GoScript reverse transcriptase kit (Promega). RT-qPCR was performed using FIREPoly qPCR Master Mix (Solis BioDyne) and analyzed using a Lightcycler 480 system (Roche). Fold changes were calculated using the ΔΔCt method. Primers used for RT-qPCR are listed in the supplemental methods (Table S7).

Protein isolation and mass spectrometry

Protein was isolated from brain tissue using N-PER Neuronal Protein Extraction Reagent (Thermo Fisher Scientific), supplemented with a protease inhibitor cocktail (Roche) according to the manufacturer's instructions. One-hundred milligrams of brain tissue was homogenized in 1 mL of N-PER reagent using Dounce homogenization (Sigma-Aldrich) and 15 strokes on ice. The homogenates were incubated on ice for 10 min, followed by centrifugation for 10 min at 4°C and 10,000 × *g*. Supernatants were collected and protein concentrations of all samples were determined using the Pierce Bicinchoninic Acid (BCA) Protein Assay Kit (Thermo Fisher Scientific).

For identification and quantification by label-free mass spectrometry quantification (LFQ), proteins were first reduced and alkylated using Tris(2-carboxyethyl)-phosphine and 2-chloroacetamide (30 min at 30°C and 700 rpm). Samples were subsequently diluted with pure ethanol (final concentration of 60% v/v) and carboxylated magnetic beads were added to the samples to bind proteins (30 min at room temperature). Next, beads were washed three times with ethanol (80% v/v) and added to trypsin (in 50 mM tetraethylammonium bromide) for overnight enzymatic digestion at 37°C. Finally, remaining peptides were extracted from beads with H_2O. Digested samples were dissolved in aqueous 3% acetonitrile with 0.1% formic acid and the peptide concentration was estimated with the Lunatic UV/Vis absorbance spectrometer. Peptides were separated on an M-class UPLC (ultra-performance liquid chromatography) and analyzed on a Iontrap mass spectrometer (Thermo). Protein identification and quantification were performed using the MaxQuant software[55] and Andromeda search engine.[56] Proteins identified as contaminants were excluded from analysis. Proteins with a minimum of two peptides, consisting of more than seven amino acids, and a false discovery rate (FDR) of 1% at the peptide and protein level were considered for further analyses. Peptides were searched against the UniProt proteome database. Protein identification required at least one unique peptide. Data were log 2-transformed and differential protein expression analysis was performed in R using the limma package.[57] Statistical significance was defined as $p < 0.05$ with Benjamini-Hochberg's test for multiple comparison adjustment. The data were further filtered to identify proteins with at least ±1.5 fold change. LFQ intensities were z scored and hierarchical clustering was performed using the ComplexHeatmap R package.[58] Gene Ontology analysis was performed using the Web-based Gene Set Analysis Toolkit.[59] Bioinformatic analyses and visualizations were performed in R.

Single-cell isolation by MACS

PBS-perfused brains were cut into small pieces and dissociated using the Adult Brain Dissociation Kit and gentleMACS Octo Dissociator with heaters (Miltenyi Biotec) according to the manufacturer's instructions. Cell debris and myelin were subsequently removed using a debris removal solution (Miltenyi Biotec). The single-cell suspension was used for the isolation of astrocytes and oligodendrocytes using the anti-ACSA-2 MicroBead kit (Miltenyi Biotec) and anti-O4 MicroBeads (Miltenyi Biotec) according to the manufacturer's instructions. The identity of all fractions was confirmed by flow cytometry.

Quantification of cell populations by flow cytometry

For analysis of astrocytes, oligodendrocytes, neurons, and microglia/macrophage populations, PBS-perfused brains from 8-month-old female animals were used. Poorly perfused brains were excluded from the analysis. After isolation, brains were stored in ice-cold 1× Hank's balanced salt solution (HBSS) (without calcium, without magnesium; Thermo Fisher Scientific). One hemisphere was dissociated as previously described[60] with minor adaptations. Briefly, the Neural Dissociation Kit (Miltenyi Biotec) was used to enzymatically digest the tissue in a two-step enzyme incubation. Dissociated tissue was passed through a 70-μm cell strainer (Corning) and resuspended in HBSS. A myelin gradient buffer (10× Percoll PLUS [Sigma-Aldrich] in HBSS) was added to this suspension at a final concentration of 30%. Samples were gently mixed before centrifugation (without brake or acceleration; 35 min at 4°C). Myelin and supernatant were carefully removed and the cell pellet was resuspended in FACS buffer (5% FBS, PBS [both from Thermo Fisher Scientific]) and 1% bovine serum albumin (BSA, Sigma-Aldrich).

Antibody stainings for flow cytometry were performed as previously described.[60] Each sample was first diluted to an equal number of cells. Remaining cells were pooled and used as single-stain and fluorescence minus one (FMO) controls. Cells for the positive control in the viability assay were heated to 60°C for 2 min. Cells were blocked in Fc CD16/CD32 block (final concentration, 5 μg/mL; BD Pharmingen) for 10 min at 4°C. Next, cells were incubated in an antibody mix in FACS buffer at 4°C. After 15 min, cells were washed in 1 mL of PBS and centrifuged at 500 × *g* for 5 min. The supernatant was removed and the cells were resuspended in streptavidin (final concentration, 1 μg/mL, Brilliant Violet 421-conjugated; BioLegend) in FACS buffer

for another 10-min incubation at 4°C. Streptavidin was diluted in 50 μL of Brilliant Stain Buffer (BD Horizon) and 50 μL of PBS. Cells were washed in 1 mL of PBS and centrifuged once more. After removal of the supernatant, cells were resuspended in 300 μL of FACS buffer and 5 μL of 7-AAD viability dye (BioLegend Europe) was added to the samples as per manufacturer's instructions. UltraComp eBeads Compensation Beads (Thermo Fisher Scientific) were used for single-stain controls for CD11b and CD45 and were processed the same way except they were not incubated with Fc block or 7-AAD. Samples were analyzed and sorted on a BD Aria Fusion cell sorter immediately after staining.

To determine the appropriate FSC/SSC gates for downstream gating to sort neural populations, samples were first visually analyzed with an Attune Cytpix flow cytometer model number 2IAFC00080820). For gating and sorting, BD FACSDiva Software (version 8.0.1) associated with the BD FACSAria Fusion sorter was used. FlowJo software (version 10.8.1) was used to analyze the Attune Cytpix flow cytometry data and to create a representative example of the gating strategy. Representative dot plots of the gating strategy for astrocytes ($CD45^-$, $ACSA\text{-}2^+$), neurons ($CD45^-$, $ACSA\text{-}2^-$, $O4^-$, $CD90.2^+$), oligodendrocytes ($CD45^-$, $O4^+$), and microglia/macrophages ($CD45^+$, $CD11b^+$) are shown in Figure S7. Flow cytometry antibodies are listed in Table S2.

Amplification for deep sequencing

Efi2b4- or *Eif2b5*-specific oligos were used to generate targeted amplicons for deep sequencing. Input genomic DNA was first amplified in a 10-μL reaction for 30 cycles using NEBNext High-Fidelity 2×PCR Master Mix (NEB). Amplicons were purified using AMPure XP beads (Beckman Coulter) and subsequently amplified for eight cycles using oligos with sequencing adapters. Approximately equal amounts of PCR products were pooled, gel purified, and quantified using a Qubit 3.0 fluorometer and the dsDNA HS Assay Kit (Thermo Fisher Scientific). Paired-end sequencing of purified libraries was performed on an Illumina Miseq. Oligos for deep sequencing are listed in the supplemental methods (Table S8).

Next generation sequencing data analysis

Sequencing reads were first demultiplexed using the Miseq Reporter (Illumina). Next, amplicon sequences were aligned to their reference sequences using CRISPResso2.[61] Base-editing efficiencies on different adenines within the spacer were calculated as percentage of (number of reads containing only the desired edit)/(number of total aligned reads). Indel rates were calculated as percentage of (number of indel-containing reads)/(total aligned reads). Reference nucleotide sequences are listed in Table S3.

Guide-dependent off-target prediction and analysis by CHANGE-seq

For CHANGE-seq, the protospacer of each sgRNA was first tested for functionality: a 517- or 503-bp-long fragment was PCR amplified from genomic DNA, isolated from the tail of a homozygous *Eif2b4*^{P244L} or *Eif2b5*^{R191H} mouse, using GoTaq G2 Hot Start Green Master Mix (Promega). Oligos are listed in the supplemental methods (Table S9). The library was prepared as previously described.[43] Purified *Sp*G nuclease was used for CHANGE-seq experiments.[62] Data were processed using v1.1 of the CHANGE-seq analysis pipeline (https://github.com/tsailabSJ/changeseq) with parameters: "window_size: 3; mapq_threshold: 50; start_threshold: 1; gap_threshold: 3; mismatch_threshold: 15; merged_analysis: True; variant_analysis: True." The top five off-target sites for the protospacer of each sgRNA were selected for targeted amplicon deep sequencing and covered by at least 20,000 reads per site. The mismatch threshold parameter was reduced to 6.

Immunohistochemistry

PFA-fixed brain tissues of C57BL/6J and *Eif2b4*^{P244L} mice were frozen on dry ice and cut into 40-μm-thick sections using a microtome. Sections were blocked in PBS supplemented with 2% normal donkey serum (catalog no. ab7475, Abcam) and 0.3% Triton X-100 (Sigma-Aldrich) for 1 h. Brain sections were incubated with primary antibodies overnight at 4°C (rabbit anti-NeuN, 1:1,000, Abcam 177487; chicken anti-GFAP, 1:1,500, Abcam ab95231). Donkey anti-rabbit-488 (1:1,000), and donkey anti-chicken-647 (1:500; all from Jackson ImmunoResearch) were used as secondary antibodies and sections were counterstained with 4′,6-diamidino-2-phenylindole (DAPI, Sigma-Aldrich). Mounting was performed using Prolong Gold Antifade Mountant (Thermo Fisher Scientific). Confocal images were taken with a Zeiss LSM 800 or a Zeiss AxioScan.Z1 slide scanner and analyzed with Fiji[63] or cell profiler.[64] Antibodies are listed in Table S2.

PFA-fixed cerebella of *Eif2b5*^{R191H} mice were embedded in paraffin and cut into 6-μm-thick sections using a microtome. Sections were subsequently deparaffinized and rehydrated. PFA-fixed hemispheres without cerebella were embedded in Tissue-Tek O.C.T. (Sakura Finetek), frozen on dry ice in 2-methylbutane, and cut into 12-μm-thick sections using a Cryostar NX50 Cryostat (Thermo Fisher Scientific). Sections were collected on SuperFrost Plus adhesive microscope slides (Thermo Fisher Scientific) and washed three times for 5 min in 1× PBS (VWR). Sections received heat-mediated antigen retrieval treatment in citrate buffer (0.01 M, pH 6; 0.1 M sodium citrate [Sigma-Aldrich], 0.1 M citric acid [Sigma-Aldrich] in MilliQ H_2O) at 121°C for 15 min. Sections were permeabilized in 0.1% Triton X-100 (w/v in PBS, Sigma-Aldrich) for 10 min, followed by 1 h of blocking in 3% BSA (w/v in PBS, Sigma-Aldrich) at room temperature. The sections were incubated overnight at 4°C with primary antibodies (mouse anti-nestin, 1:500, BD Transduction Laboratories, 611658; rabbit anti-GFAP, 1:1,000; Dako Z0334 and/or rabbit anti-S100β, 1:1,000, ProteinTech 15146-1-AP), diluted in 3% BSA solution. The next day, sections were washed once in PBS and incubated with secondary antibodies (goat anti-mouse-488, 1:1,000, Invitrogen A31620; goat anti-rabbit, 1:1,000; Invitrogen A31632) for 2 h at room temperature, followed by counterstaining with DAPI (1:2,000; Sigma-Aldrich) and embedding in Fluoromount G (Thermo Fisher Scientific). Images were taken with a Leica DM5000B microscope (Leica Microsystems). Nestin-GFAP double-positive astrocytes and DAPI-positive nuclei in the corpus callosum were counted in two standardized fields per

animal (1× rostrum and 1× splenium) and the percentage of nestin-GFAP double-positive cells was determined over the total number of DAPI-positive nuclei. Bergmann glia translocation was determined in one to three regions of the cerebellar cortex per animal.

Histology

PFA-fixed brains, livers, and tumors were embedded in Tissue-Tek O.C.T. (Sakura Finetek) medium and cut into 7-μm-thick sections using a cryostat. Sections were hematoxylin and eosin stained and examined for histopathological changes using a Zeiss AxioScan.Z1 slide scanner. Images were analyzed with Fiji.[63]

Statistical analysis

All statistical analyses were performed using GraphPad Prism 9.5.1 for macOS. Unless stated otherwise, datasets are represented as biological replicates and are depicted as means ± standard deviation (SD). Statistical analyses are always indicated in the corresponding figure legends. Likewise, sample sizes and the statistical tests performed are described in the respective figure legends. The data were tested for normality using the Shapiro-Wilk test unless stated otherwise. For all analyses, $p < 0.05$ was considered statistically significant.

DATA AND CODE AVAILABILITY

All data associated with this study are present in the paper. Illumina sequencing data are available at the Sequence Read Archive (SRA) under BioProject accession number PRJNA1050614.

SUPPLEMENTAL INFORMATION

Supplemental information can be found online at https://doi.org/10.1016/j.ymthe.2024.03.009.

ACKNOWLEDGMENTS

We thank the Functional Genomics Center Zurich (FGCZ) for technical support and access to instruments at the University of Zurich. Furthermore, we thank Bernd Roschitzki from the FGCZ for advice with respect to protein sample preparation and analyses. Susanne Kreutzer and Zacharias Kontarakis (Genome Engineering and Measurement Lab, ETH Zürich) are acknowledged for performing CHANGE-seq analyses. We thank Natasa Savic and the ETH Zurich Phenomics Center (EPIC) for generating the *Eif2b4*^{P244L} knockin mouse model. We thank Leoni Hoogterp and Marjolein Breur (Amsterdam UMC, the Netherlands) for assistance with tissue preparation for immunohistochemistry, Seyed Mohammad Jazaeri Farsani (Amsterdam UMC) for qPCR analysis, and Truus Abbink (Amsterdam UMC) for discussion and advice. Cornelia Schwerdel (University of Zurich) is acknowledged for advice and technical support for immunohistochemistry. We thank the animal caretakers of the VU-VUmc animal facility for mouse breeding and advice. We acknowledge the Microscopy and Cytometry Core Facility at the Amsterdam UMC, Location VUmc for providing assistance in our microscopy and cytometry work. We thank Miltenyi Biotec (Leiden, the Netherlands) for providing equipment for MACS experiments. Sylics (Bilthoven, the Netherlands) is acknowledged for providing the grip strength measuring device. Members of the Schwank and van Til labs are acknowledged for discussions and comments on the manuscript. This work was supported by the Swiss National Science Foundation (SNSF) grant no. 310030_185293 (to G.S.), Novartis Foundation for Medical-Biological Research no. FN20-0000000203 (to D.B.), SNSF Spark fellowship no. 196287 (to D.B.), and the Helmut Horten Foundation F-41603-14-01 (to G.S.). M.S.v.d.K. is a member of the European Reference Network for Rare Neurological Disorders (ERNRND), project ID 739510. Work in the lab of M.A.M.-M. was supported by grants PID2021-127535NB-I00, CEX2020-001088-M, RyC-2017-23041, and UPO1380590 funded by MCIN/AEI/10.13039/501100011033, by "ERDF A way of making Europe" and "ERDF A way of making Europe," University Pablo de Olavide and Junta de Andalucía, respectively.

AUTHOR CONTRIBUTIONS

D.B., I.M.R., N.P.v.T., and G.S. designed the study. D.B. and S.E. conducted *in vitro* experiments. D.B. and L.K. performed AAV transduction experiments in C57BL/6 mice. D.B. conducted *in vivo* experiments with *Eif2b4*^{P244L} mice. I.M.R., A.S.J.B., A.E.J.H., and C.B. conducted *in vivo* experiments with *Eif2b5*^{R191H} mice. D.B. performed MACS experiments. A.E.J.H. conducted flow cytometry experiments. M.A.M-M. contributed to CHANGE-seq experiments. D.B., I.M.R., N.P.v.T., and G.S. wrote the manuscript with input from all coauthors. All authors reviewed the manuscript.

DECLARATION OF INTERESTS

G.S. is a scientific advisor to Prime Medicine.

REFERENCES

1. Stellitano, L.A., Winstone, A.M., van der Knaap, M.S., and Verity, C.M. (2016). Leukodystrophies and genetic leukoencephalopathies in childhood: a national epidemiological study. Dev. Med. Child Neurol. *58*, 680–689.

2. Van Der Knaap, M.S., Barth, P.G., Gabreëls, F.J., Franzoni, E., Begeer, J.H., Stroink, H., Rotteveel, J.J., and Valk, J. (1997). A new leukoencephalopathy with vanishing white matter. Neurology *48*, 845–855.

3. Schiffmann, R., and Elroy-Stein, O. (2006). Childhood ataxia with CNS hypomyelination/vanishing white matter disease-A common leukodystrophy caused by abnormal control of protein synthesis. Mol. Genet. Metab. *88*, 7–15.

4. van der Knaap, M.S., Pronk, J.C., and Scheper, G.C. (2006). Vanishing white matter disease. Lancet Neurol. *5*, 413–423.

5. Bugiani, M., Boor, I., Powers, J.M., Scheper, G.C., and Van Der Knaap, M.S. (2010). Leukoencephalopathy with vanishing white matter: A review. J. Neuropathol. Exp. Neurol. *69*, 987–996.

6. van der Knaap, M.S., Bonkowsky, J.L., Vanderver, A., Schiffmann, R., Krägeloh-Mann, I., Bertini, E., Bernard, G., Fatemi, S.A., Wolf, N.I., Saunier-Vivar, E., et al. (2022). Therapy Trial Design in Vanishing White Matter. Neurol. Genet. *8*, e657.

7. Hamilton, E.M.C., van der Lei, H.D.W., Vermeulen, G., Gerver, J.A.M., Lourenço, C.M., Naidu, S., Mierzewska, H., Gemke, R.J.B.J., de Vet, H.C.W., Uitdehaag, B.M.J., et al. (2018). Natural History of Vanishing White Matter. Ann. Neurol. *84*, 274–288.

8. Bugiani, M., Vuong, C., Breur, M., and van der Knaap, M.S (2018). Vanishing white matter: a leukodystrophy due to astrocytic dysfunction. Brain Pathol. *28*, 408–421.

9. Bugiani, M., Postma, N., Polder, E., Dieleman, N., Scheffer, P.G., Sim, F.J., van der Knaap, M.S., and Boor, I. (2013). Hyaluronan accumulation and arrested oligodendrocyte progenitor maturation in vanishing white matter disease. Brain *136*, 209–222.

10. Bugiani, M., Boor, I., van Kollenburg, B., Postma, N., Polder, E., van Berkel, C., van Kesteren, R.E., Windrem, M.S., Hol, E.M., Scheper, G.C., et al. (2011). Defective glial maturation in vanishing white matter disease. J. Neuropathol. Exp. Neurol. *70*, 69–82.

11. Dooves, S., Bugiani, M., Postma, N.L., Polder, E., Land, N., Horan, S.T., van Deijk, A.L.F., van de Kreeke, A., Jacobs, G., Vuong, C., et al. (2016). Astrocytes are central in the pathomechanisms of vanishing white matter. J. Clin. Invest. *126*, 1512–1524.

12. van Der Knaap, M.S., Leegwater, P.A.J., Könst, A.A.M., Visser, A., Naidu, S., Oudejans, C.B.M., Schutgens, R.B.H., and Pronk, J.C. (2002). Mutations in each of the five subunits of translation initiation factor eIF2B can cause leukoencephalopathy with vanishing white matter. Ann. Neurol. *51*, 264–270.

13. Leegwater, P.A., Vermeulen, G., Könst, A.A., Naidu, S., Mulders, J., Visser, A., Kersbergen, P., Mobach, D., Fonds, D., van Berkel, C.G., et al. (2001). Subunits of the translation initiation factor elF2B are mutant in leukoencephalopathy with vanishing white matter. Nat. Genet. *29*, 383–388.

14. Wong, Y.L., LeBon, L., Basso, A.M., Kohlhaas, K.L., Nikkel, A.L., Robb, H.M., Donnelly-Roberts, D.L., Prakash, J., Swensen, A.M., Rubinstein, N.D., et al. (2019). eIF2B activator prevents neurological defects caused by a chronic integrated stress response. Elife *8*, e42940.

15. Abbink, T.E.M., Wisse, L.E., Jaku, E., Thiecke, M.J., Voltolini-González, D., Fritsen, H., Bobeldijk, S., Ter Braak, T.J., Polder, E., Postma, N.L., et al. (2019). Vanishing white matter: deregulated integrated stress response as therapy target. Ann. Clin. Transl. Neurol. *6*, 1407–1422.

16. Dietrich, J., Lacagnina, M., Gass, D., Richfield, E., Mayer-Pröschel, M., Noble, M., Torres, C., and Pröschel, C. (2005). EIF2B5 mutations compromise GFAP+ astrocyte generation in vanishing white matter leukodystrophy. Nat. Med. *11*, 277–283.

17. Gaudelli, N.M., Komor, A.C., Rees, H.A., Packer, M.S., Badran, A.H., Bryson, D.I., and Liu, D.R. (2017). Programmable base editing of T to G C in genomic DNA without DNA cleavage. Nature *551*, 464–471.

18. Komor, A.C., Kim, Y.B., Packer, M.S., Zuris, J.A., and Liu, D.R. (2016). Programmable editing of a target base in genomic DNA without double-stranded DNA cleavage. Nature *533*, 420–424.

19. Rees, H.A., and Liu, D.R. (2018). Base editing: precision chemistry on the genome and transcriptome of living cells. Nat. Rev. Genet. *19*, 770–788.

20. Anzalone, A.V., Koblan, L.W., and Liu, D.R. (2020). Genome editing with CRISPR–Cas nucleases, base editors, transposases and prime editors. Nat. Biotechnol. *38*, 824–844.

21. van der Lei, H.D.W., van Berkel, C.G.M., van Wieringen, W.N., Brenner, C., Feigenbaum, A., Mercimek-Mahmutoglu, S., Philippart, M., Tatli, B., Wassmer, E., Scheper, G.C., and van der Knaap, M.S. (2010). Genotype-phenotype correlation in vanishing white matter disease. Neurology *75*, 1555–1559.

22. Slynko, I., Nguyen, S., Hamilton, E.M.C., Wisse, L.E., de Esch, I.J.P., de Graaf, C., Bruning, J.B., Proud, C.G., Abbink, T.E.M., and van der Knaap, M.S. (2021). Vanishing white matter: Eukaryotic initiation factor 2B model and the impact of missense mutations. Mol. Genet. Genomic Med. *9*, e1593.

23. Fogli, A., Schiffmann, R., Bertini, E., Ughetto, S., Combes, P., Eymard-Pierre, E., Kaneski, C.R., Pineda, M., Troncoso, M., Uziel, G., et al. (2004). The effect of genotype on the natural history of eIF2B-related leukodystrophies. Neurology *62*, 1509–1517.

24. Scali, O., Di Perri, C., and Federico, A. (2006). The spectrum of mutations for the diagnosis of vanishing white matter disease. Neurol. Sci. *27*, 271–277.

25. Woodard, L.E., and Wilson, M.H. (2015). PiggyBac-ing models and new therapeutic strategies. Trends Biotechnol. *33*, 525–533.

26. Richter, M.F., Zhao, K.T., Eton, E., Lapinaite, A., Newby, G.A., Thuronyi, B.W., Wilson, C., Koblan, L.W., Zeng, J., Bauer, D.E., et al. (2020). Phage-assisted evolution of an adenine base editor with improved Cas domain compatibility and activity. Nat. Biotechnol. *38*, 883–891.

27. Witkamp, D., Oudejans, E., Hu-A-Ng, G.V., Hoogterp, L., Krzywańska, A.M., Žnidaršič, M., Marinus, K., de Veij Mestdagh, C.F., Bartelink, I., Bugiani, M., et al. (2022). Guanabenz ameliorates disease in vanishing white matter mice in contrast to sephin1. Ann. Clin. Transl. Neurol. *9*, 1147–1162.

28. Passini, M.A., Watson, D.J., and Wolfe, J.H. (2004). Gene delivery to the mouse brain with adeno-associated virus. Methods Mol. Biol. *246*, 225–236.

29. Chan, K.Y., Jang, M.J., Yoo, B.B., Greenbaum, A., Ravi, N., Wu, W.L., Sánchez-Guardado, L., Lois, C., Mazmanian, S.K., Deverman, B.E., and Gradinaru, V. (2017). Engineered AAVs for efficient noninvasive gene delivery to the central and peripheral nervous systems. Nat. Neurosci. *20*, 1172–1179.

30. Gray, S.J., Foti, S.B., Schwartz, J.W., Bachaboina, L., Taylor-Blake, B., Coleman, J., Ehlers, M.D., Zylka, M.J., McCown, T.J., and Samulski, R.J. (2011). Optimizing promoters for recombinant adeno-associated virus-mediated gene expression in the peripheral and central nervous system using self-complementary vectors. Hum. Gene Ther. *22*, 1143–1153.

31. Mathiesen, S.N., Lock, J.L., Schoderboeck, L., Abraham, W.C., and Hughes, S.M. (2020). CNS Transduction Benefits of AAV-PHP.eB over AAV9 Are Dependent on Administration Route and Mouse Strain. Mol. Ther. Methods Clin. Dev. *19*, 447–458.

32. Grieger, J.C., and Samulski, R.J. (2005). Packaging Capacity of Adeno-Associated Virus Serotypes: Impact of Larger Genomes on Infectivity and Postentry Steps. J. Virol. *79*, 9933–9944.

33. Villiger, L., Grisch-Chan, H.M., Lindsay, H., Ringnalda, F., Pogliano, C.B., Allegri, G., Fingerhut, R., Häberle, J., Matos, J., Robinson, M.D., et al. (2018). Treatment of a metabolic liver disease by *in vivo* genome base editing in adult mice. Nat. Med. *24*, 1519–1525.

34. Levy, J.M., Yeh, W.H., Pendse, N., Davis, J.R., Hennessey, E., Butcher, R., Koblan, L.W., Comander, J., Liu, Q., and Liu, D.R. (2020). Cytosine and adenine base editing of the brain, liver, retina, heart and skeletal muscle of mice via adeno-associated viruses. Nat. Biomed. Eng. *4*, 97–110.

35. Koblan, L.W., Erdos, M.R., Wilson, C., Cabral, W.A., Levy, J.M., Xiong, Z.M., Tavarez, U.L., Davison, L.M., Gete, Y.G., Mao, X., et al. (2021). *In vivo* base editing rescues Hutchinson–Gilford progeria syndrome in mice. Nature *589*, 608–614.

36. Guyenet, S.J., Furrer, S.A., Damian, V.M., Baughan, T.D., la Spada, A.R., and Garden, G.A. (2010). A simple composite phenotype scoring system for evaluating mouse models of cerebellar ataxia. J. Vis. Exp. *39*, 1787.

37. Hillen, A.E.J., Hruzova, M., Rothgangl, T., Breur, M., Bugiani, M., van der Knaap, M.S., Schwank, G., and Heine, V.M. (2022). *In vivo* targeting of a variant causing vanishing white matter using CRISPR/Cas9. Mol. Ther. Methods Clin. Dev. *25*, 17–25.

38. Man, J.H.K., van Gelder, C.A.G.H., Breur, M., Molenaar, D., Abbink, T., Altelaar, M., Bugiani, M., and van der Knaap, M.S. (2023). Regional vulnerability of brain white matter in vanishing white matter. Acta Neuropathol. Commun. *11*, 103.

39. van der Knaap, M.S., and Bugiani, M. (2017). Leukodystrophies: a proposed classification system based on pathological changes and pathogenetic mechanisms. Acta Neuropathol. *134*, 351–382.

40. Dooves, S., Bugiani, M., Wisse, L.E., Abbink, T.E.M., van der Knaap, M.S., and Heine, V.M. (2018). Bergmann glia translocation: a new disease marker for vanishing white matter identifies therapeutic effects of Guanabenz treatment. Neuropathol. Appl. Neurobiol. *44*, 391–403.

41. Wu, Y., Pan, Y., Du, L., Wang, J., Gu, Q., Gao, Z., Li, J., Leng, X., Qin, J., Wu, X., and Jiang, Y. (2009). Identification of novel EIF2B mutations in Chinese patients with vanishing white matter disease. J. Hum. Genet. *54*, 74–77.

42. Zhang, H., Dai, L., Chen, N., Zang, L., Leng, X., Du, L., Wang, J., Jiang, Y., Zhang, F., Wu, X., and Wu, Y. (2015). Fifteen Novel EIF2B1-5 Mutations Identified in Chinese Children with Leukoencephalopathy with Vanishing White Matter and a Long Term Follow-Up. PLoS One *10*, e0118001.

43. Lazzarotto, C.R., Malinin, N.L., Li, Y., Zhang, R., Yang, Y., Lee, G., Cowley, E., He, Y., Lan, X., Jividen, K., et al. (2020). CHANGE-seq reveals genetic and epigenetic effects on CRISPR–Cas9 genome-wide activity. Nat. Biotechnol. *38*, 1317–1327.

44. Sanders, M.A., Ampasala, D., and Basson, M.D. (2009). DOCK5 and DOCK1 regulate Caco-2 intestinal epithelial cell spreading and migration on collagen IV. J. Biol. Chem. *284*, 27–35.

45. Liaci, A.M., Steigenberger, B., Telles de Souza, P.C., Tamara, S., Gröllers-Mulderij, M., Ogrissek, P., Marrink, S.J., Scheltema, R.A., and Förster, F. (2021). Structure of the human signal peptidase complex reveals the determinants for signal peptide cleavage. Mol. Cell *81*, 3934–3948.e11.

46. Sjöstedt, E., Zhong, W., Fagerberg, L., Karlsson, M., Mitsios, N., Adori, C., Oksvold, P., Edfors, F., Limiszewska, A., Hikmet, F., et al. (2020). An atlas of the protein-coding genes in the human, pig, and mouse brain. Science *367*, eaay5947.

47. Francis, J.S., Markov, V., Wojtas, I.D., Gray, S., McCown, T., Samulski, R.J., Figueroa, M., and Leone, P. (2021). Preclinical biodistribution, tropism, and efficacy of oligotropic AAV/Olig001 in a mouse model of congenital white matter disease. Mol. Ther. Methods Clin. Dev. *20*, 520–534.

48. Anzalone, A.V., Randolph, P.B., Davis, J.R., Sousa, A.A., Koblan, L.W., Levy, J.M., Chen, P.J., Wilson, C., Newby, G.A., Raguram, A., and Liu, D.R. (2019). Search-and-replace genome editing without double-strand breaks or donor DNA. Nature *576*, 149–157.

49. Böck, D., Rothgangl, T., Villiger, L., Schmidheini, L., Matsushita, M., Mathis, N., Ioannidi, E., Rimann, N., Grisch-Chan, H.M., Kreutzer, S., et al. (2022). *In vivo* prime editing of a metabolic liver disease in mice. Sci. Transl. Med. *14*, eabl9238.

50. Düring, D.N., Dittrich, F., Rocha, M.D., Tachibana, R.O., Mori, C., Okanoya, K., Boehringer, R., Ehret, B., Grewe, B.F., Gerber, S., et al. (2020). Fast Retrograde Access to Projection Neuron Circuits Underlying Vocal Learning in Songbirds. Cell Rep. *33*, 108364.

51. Lampe, S.E.G., Kaspar, B.K., and Foust, K.D. (2014). Intravenous injections in neonatal mice. J. Vis. Exp. 93:e52037.

52. Yardeni, T., Eckhaus, M., Morris, H.D., Huizing, M., and Hoogstraten-Miller, S. (2011). Retro-orbital injections in mice. Lab Anim. *40*, 155–160.

53. Spijker, S. (2011). Neuroproteomics: Dissection of Rodent Brain Regions. Neuromethods *15*, 66–74.

54. Spijker, S., Faliagkas, L., and Rao-Ruiz, P. (2019). Dissection of rodent brain regions: Guided free-hand slicing and dissection of frozen tissue. Neuromethods *146*, 7–19.

55. Cox, J., and Mann, M. (2008). MaxQuant enables high peptide identification rates, individualized p.p.b.-range mass accuracies and proteome-wide protein quantification. Nat. Biotechnol. *26*, 1367–1372.

56. Cox, J., Neuhauser, N., Michalski, A., Scheltema, R.A., Olsen, J.V., and Mann, M. (2011). Andromeda: A peptide search engine integrated into the MaxQuant environment. J. Proteome Res. *10*, 1794–1805.

57. Ritchie, M.E., Phipson, B., Wu, D., Hu, Y., Law, C.W., Shi, W., and Smyth, G.K. (2015). Limma powers differential expression analyses for RNA-sequencing and microarray studies. Nucleic Acids Res. *43*, e47.

58. Gu, Z., Eils, R., and Schlesner, M. (2016). Complex heatmaps reveal patterns and correlations in multidimensional genomic data. Bioinformatics *32*, 2847–2849.

59. Liao, Y., Wang, J., Jaehnig, E.J., Shi, Z., and Zhang, B. (2019). WebGestalt 2019: gene set analysis toolkit with revamped UIs and APIs. Nucleic Acids Res. *47*, W199–W205.

60. Estevez, F.J.M., Mathews, T.D., Biffi, A., and Peviani, M. (2019). Simultaneous flow cytometric characterization of multiple cell types retrieved from mouse brain/spinal cord through different homogenization methods. J. Vis. Exp. 153:e60335. https://doi.org/10.3791/60335.

61. Clement, K., Rees, H., Canver, M.C., Gehrke, J.M., Farouni, R., Hsu, J.Y., Cole, M.A., Liu, D.R., Joung, J.K., Bauer, D.E., and Pinello, L. (2019). CRISPResso2 provides accurate and rapid genome editing sequence analysis. Nat. Biotechnol. *37*, 224–226.

62. Vicencio, J., Sánchez-Bolaños, C., Moreno-Sánchez, I., Brena, D., Vejnar, C.E., Kukhtar, D., Ruiz-López, M., Cots-Ponjoan, M., Rubio, A., Melero, N.R., et al. (2022). Genome editing in animals with minimal PAM CRISPR-Cas9 enzymes. Nat. Commun. *13*, 2601.

63. Schindelin, J., Arganda-Carreras, I., Frise, E., Kaynig, V., Longair, M., Pietzsch, T., Preibisch, S., Rueden, C., Saalfeld, S., Schmid, B., et al. (2012). Fiji: An open-source platform for biological-image analysis. Nat. Methods *9*, 676–682.

64. Stirling, D.R., Swain-Bowden, M.J., Lucas, A.M., Carpenter, A.E., Cimini, B.A., and Goodman, A. (2021). CellProfiler 4: improvements in speed, utility and usability. BMC Bioinformatics *9*, 676–682.

YMTHE, Volume *32*

Supplemental Information

In vivo base editing of a pathogenic *Eif2b5* variant improves vanishing white matter phenotypes in mice

Desirée Böck, Ilma M. Revers, Anastasia S.J. Bomhof, Anne E.J. Hillen, Claire Boeijink, Lucas Kissling, Sabina Egli, Miguel A. Moreno-Mateos, Marjo S. van der Knaap, Niek P. van Til, and Gerald Schwank

Supplemental figures and legends

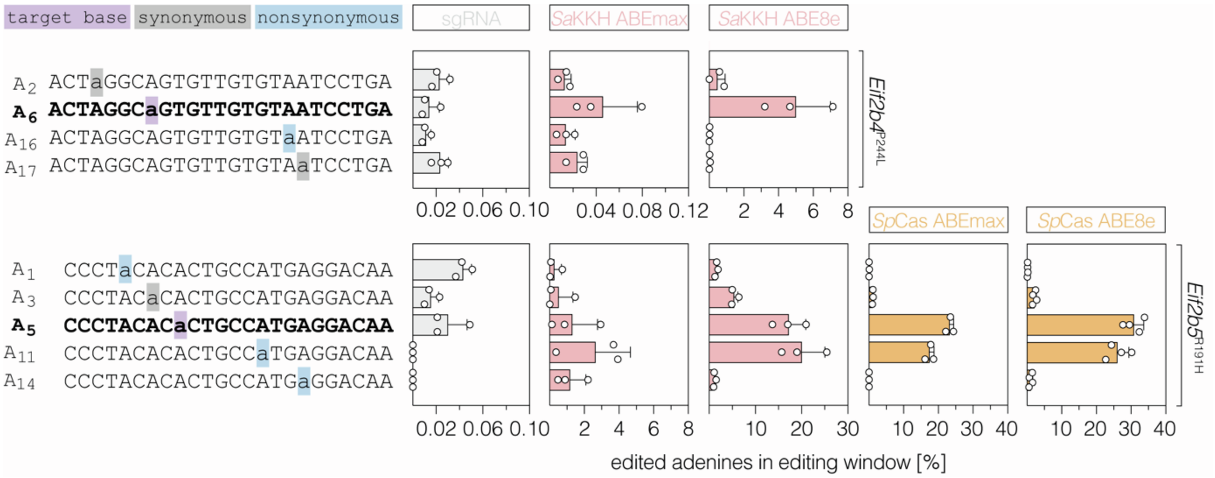

Figure S1 | On-target and bystander editing at pathogenic VWM variants in HEK cell lines. Editing outcomes at the indicated adenines at the *Eif2b4*^{P244L} (top) and *Eif2b5*^{R191H} locus (bottom) for *Sa*KKH- (sgRNA1.1 for *Eif2b4*^{P244L}; sgRNA2.1 for *Eif2b5*^{R191H}) and *Sp*Cas-ABE variants (sgRNA2.2 for *Eif2b5*^{R191H}) in HEK cell lines, where the corresponding VWM mouse variant was integrated into the genome using the PiggyBac transposon system. On-target editing (purple) as well as synonymous (gray) and nonsynonymous bystanders (blue) are highlighted in the indicated colors. The desired editing outcome with only precise on-target editing is shown in bold. The numbering of adenines (A_x) is based on their position within the combined protospacer sequence of all used ABE variants (Fig. 1A). Control samples were transfected with sgRNA only. The used ABE variants are color-coded and indicated above the respective plot. Data are displayed as means ± SD of 3 or 4 independent experiments. Related to figure 1.

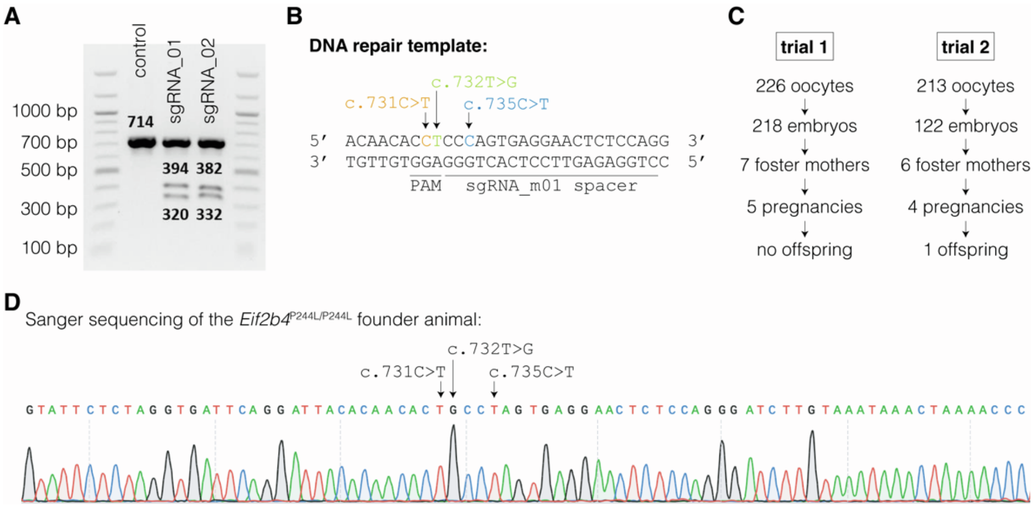

Figure S2 | Generation of a knock-in *Eif2b4*$^{\mathrm{P244L}}$ VWM mouse model. (**A**) *In vitro* validation of the activity of two sgRNAs (01 and 02) on *Eif2b4* template DNA. (**B**) Representation of the target site within the DNA repair template. Silent mutations (c.732T>G and c.735C>T) were included together with the target mutation (c.731C>T) in the repair template to avoid re-targeting of the site by the RNP complex after recombination. The PAM and spacer sequence are indicated. (**C**) Depiction of the experimental steps performed and animal numbers used for generating the *Eif2b4*$^{\mathrm{P244L}}$ mouse model. (**D**) Homozygous integration of the repair template at the intended *Eif2b4* locus in the founder animal. Related to figure 2.

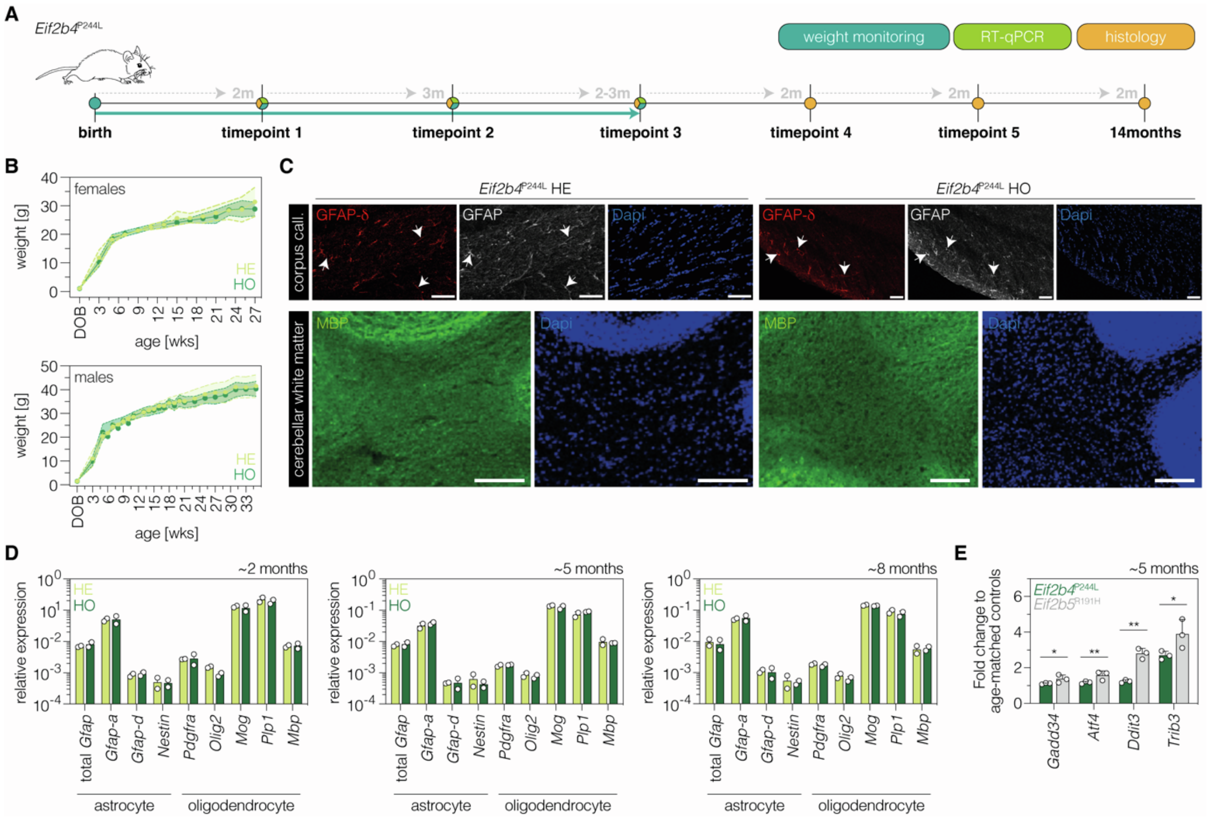

Figure S3 | Phenotypic characterization of the *Eif2b4*^{P244L} knock-in mouse model. (**A**) Schematic representation of the experimental timeline and setup. The timepoints at which weight monitoring (green), RT-qPCR analysis (light green), and histology (orange) were performed are indicated by color along the experiment timeline. (**B**) Weekly weight progression in heterozygous (HE) and homozygous (HO) *Eif2b4*^{P244L} mice (separated by gender). (**C**) Representative images showing GFAP and GFAP-δ expression in white matter astrocytes (white arrowheads) in the corpus callosum and lack of myelin vacuolization in cerebellar white matter. The *Eif2b4* genotype is indicated at the top. (**D**) Forebrain transcript levels of astrocyte (*Gfap*, *Gfap-a*, *Gfap-d*, and *Nestin*) and oligodendrocyte markers (*Pdgfra*, *Olig2*, *Mog*, *Plp1*, and *Mbp*). (**E**) Comparison of ISR transcript levels in homozygous (HO) *Eif2b4*^{P244L} and *Eif2b5*^{R191H} mice at 8 months of age. Age-matched healthy controls were used for quantification of fold changes in the corresponding model. Transcript levels were normalized to *Akt*. Data are displayed as means ± range of 2 animals per group (A and D), means ± SD of 3 animals per group (B and E), and were analyzed using an unpaired two-tailed Student's t-test (E; *$P<0.05$; **$P<0.005$). Unless otherwise indicated, statistical differences were not significant. DOB, date of birth; wks, weeks; m, month(s), g, gram; wt, wild type; cc, corpus callosum; HE, heterozygous; HO, homozygous; *Gfap*/GFAP, glial fibrillary acidic protein; *Gfap-d*/GFAP-δ, delta isoform of glial fibrillary acidic protein; *Gfap-a*, alpha isoform of glial fibrillary acidic protein; *Pdgfra*, platelet-derived growth factor receptor alpha; *Olig2*, oligodendrocyte transcription factor 2; *Mog*, myelin oligodendrocyte glycoprotein; *Plp1*, proteolipid protein 1; *Mbp*, myelin basic protein; *Nestin*, neuroepithelial stem cell protein. *Trib3*, Tribbles Pseudokinase 3; *Atf4*, activating transcription factor 4; *Ddit3*, DNA damage inducible transcript 3; Gadd34, growth arrest and DNA damage-inducible protein 34. Scale bars, 100µm. Related to figure 2.

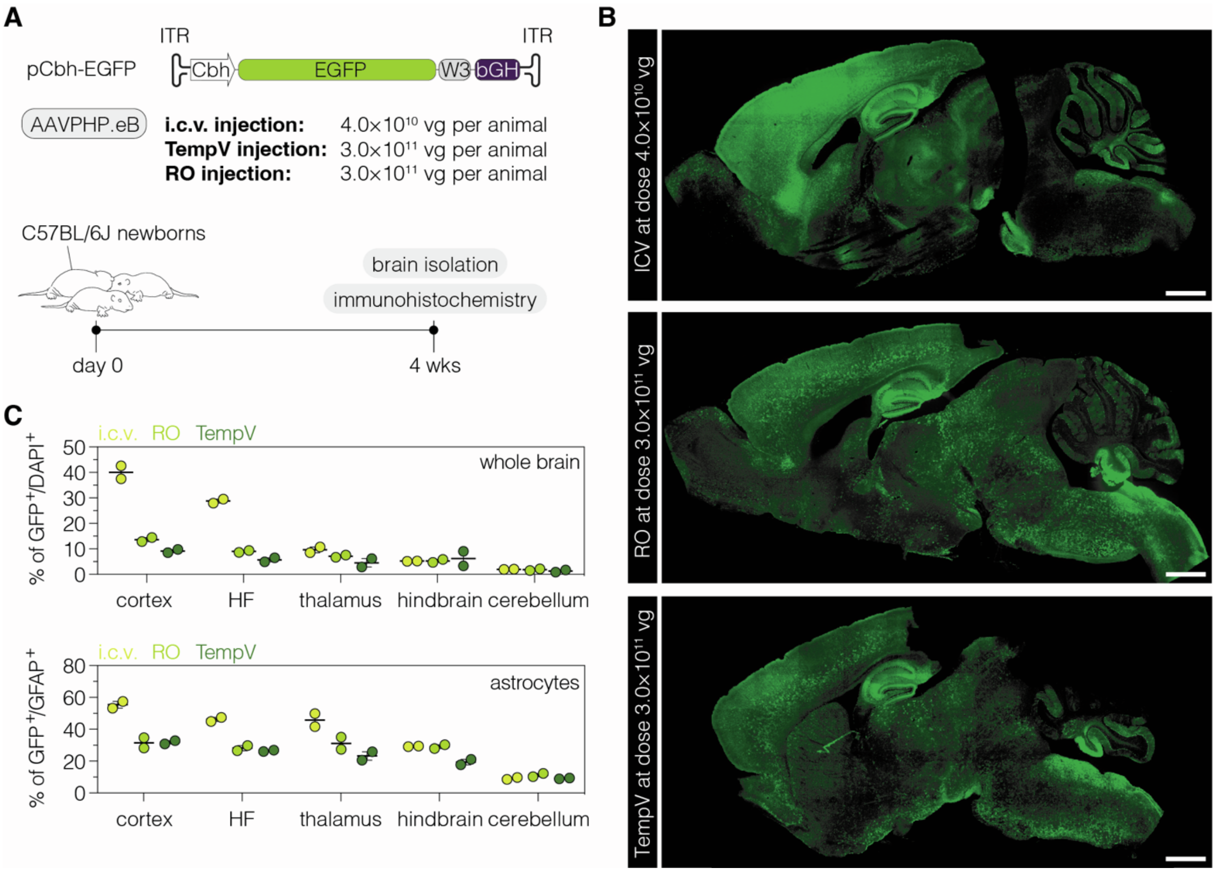

Figure S4 | AAV-PHP.eB transduction efficiencies in the newborn brain upon systemic or intracranial administration. (**A**) Schematic representation of the experimental setup and timeline for analysis of transduction efficiencies in the brain. Maximum AAV doses were injected for each application route. (**B**) Representative whole-brain overview images of EGFP fluorescence after 30 days of expression. AAV particles, expressing EGFP under the Cbh promoter, were delivered to newborn mice via the indicated route and at the described dose. (**C**) Quantifications of total transduction efficiency (top, displayed as percentage of $GFP^{+}/DAPI^{+}$), and astrocyte transduction efficiency (bottom, displayed as percentage of $GFP^{+}/GFAP^{+}$) in different brain regions. Data are displayed as means ± range of 2 mice per group. Wks, weeks; i.c.v., intracerebroventricular; RO, retro-bulbar; TempV, temporal vein; vg, vector genomes; EGFP, enhanced green fluorescent protein; HF, hippocampal formation. Scale bars, 1000µm. Related to figure 2.

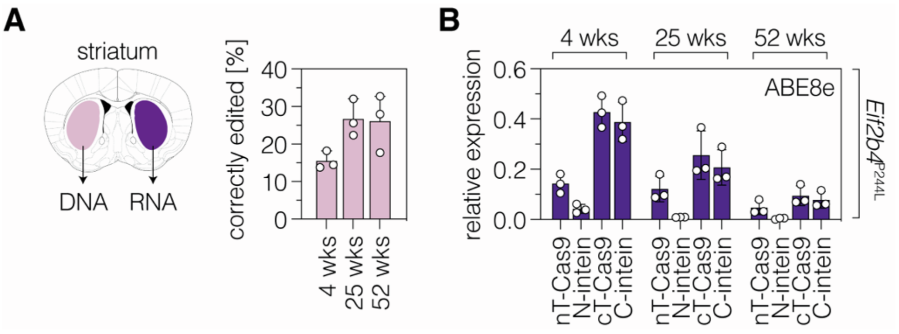

Figure S5 | Time course of editing rates and ABE8e expression in mouse striata over time. (**A,B**) Editing rates (A) and ABE8e transcript levels (B) of n- and c-terminal AAV preparations in striata of *Eif2b4*^{P244L} mice (n=3). Transcripts were normalized to *Gapdh*. Corresponding editing rates of the same mice are shown for reference. Tissue regions used for DNA and RNA isolations are shown on the brain atlas (left).[65] Data are displayed as means ± SD of 3 mice per group. nT, N-terminal AAV construct; cT, C-terminal AAV construct; wks, weeks. Related to figure 3.

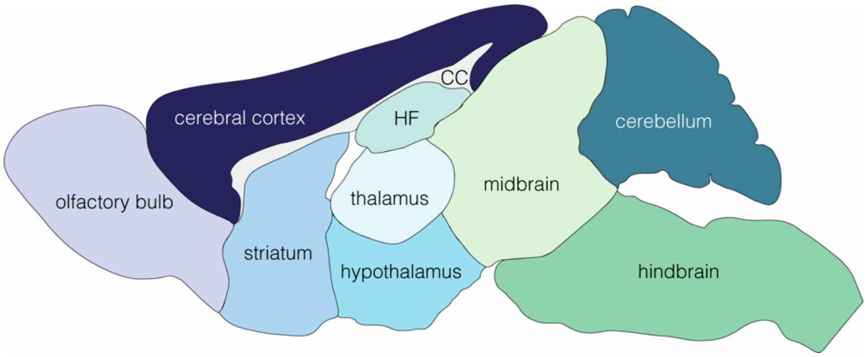

Figure S6 | Dissection of the mouse brain into 10 distinct regions. Schematic representation marking the dissection borders for isolation of the olfactory bulb, cerebral cortex, corpus callosum (CC), hippocampal formation (HF), thalamus, striatum, hypothalamus, midbrain, hindbrain, and cerebellum. Regions are depicted approximately to scale. Related to figure 5.

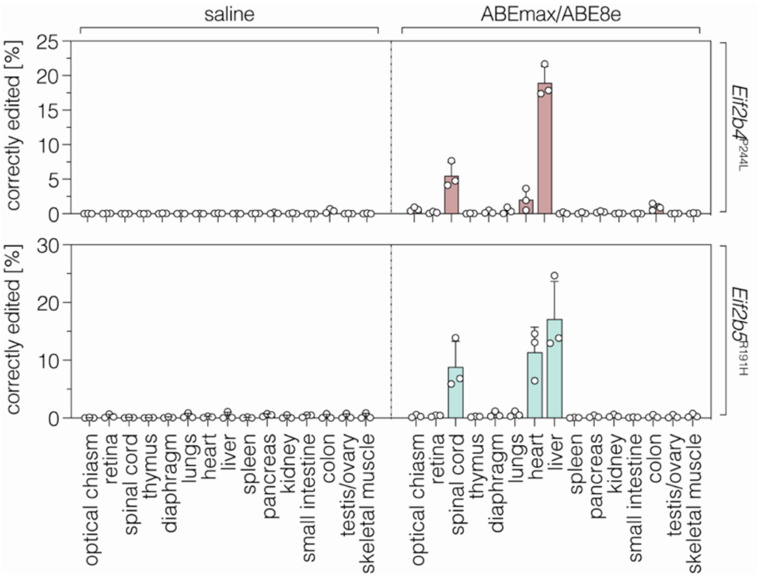

Figure S7 | *In vivo* base editing at the *Eif2b4* and *Eif2b5* locus outside the brain. On-target editing rates at the *Eif2b4* (top) and *Eif2b5* (bottom) locus in saline- (left) and ABEmax/ABE8e-treated mice (right). Data are displayed as means ± SD of 3 animals per group and genotype. Related to figure 5.

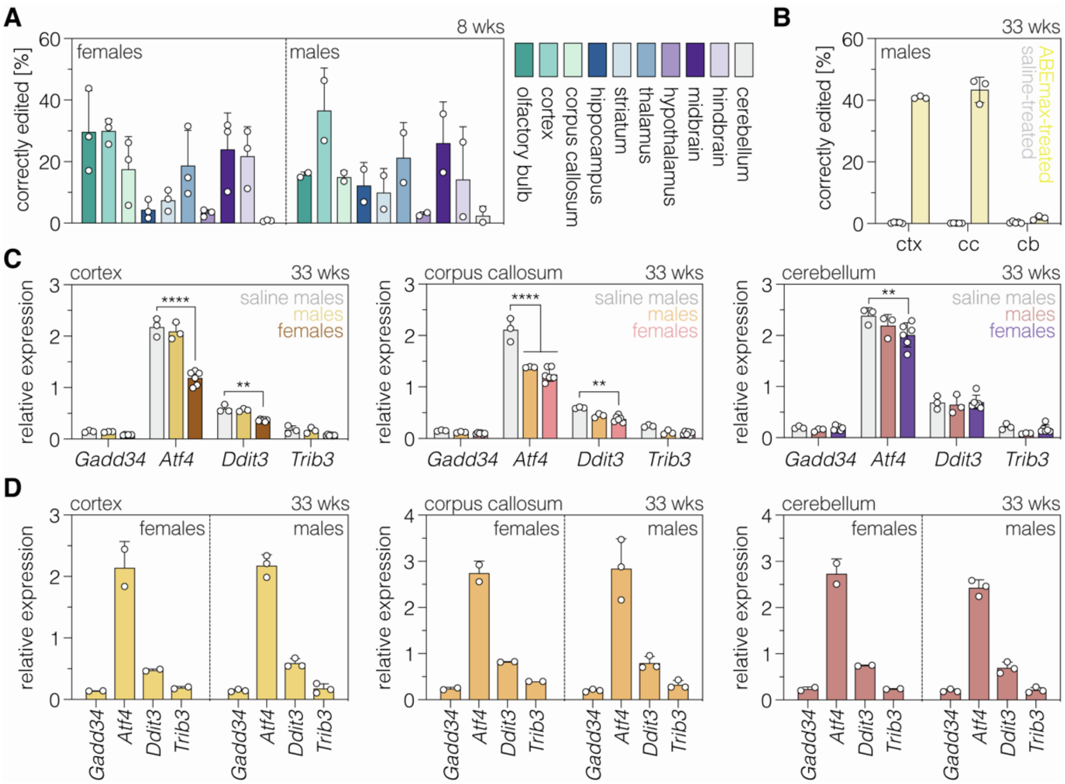

Figure S8 | Base editing and transcript levels of ISR regulators across different brain regions in female and male *Eif2b5*^{R191H} mice. (**A**) Editing rates at the *Eif2b5* locus in different brain regions at 8 weeks post-treatment in female (n=3) and male mice (n=2). (**B,C**) Endpoint editing rates (B) and corresponding transcript levels of ISR regulators (C) in the cortex (ctx), corpus callosum (cc), and cerebellum (cb) of saline- and ABEmax-treated male *Eif2b5*^{R191H} mice (n=3 mice per group). Transcript levels of ABEmax-treated female *Eif2b5*^{R191H} mice are shown for comparison (n=6 mice per group) in (C). (**D**) Transcript levels of ISR regulators in saline-treated female (n=2) and male *Eif2b5*^{R191H} mice (n=3). All transcripts were normalized to *Akt* (C and D). Data are displayed as means ± SD of 2-6 animals per group and were analyzed using a two-way ANOVA with Sidak's multiple comparisons test (C; ***P<0.005*; *****P<0.0001*). Unless otherwise indicated, statistical differences were not significant. ctx, cortex; cc, corpus callosum; cb, cerebellum. Related to figure 5.

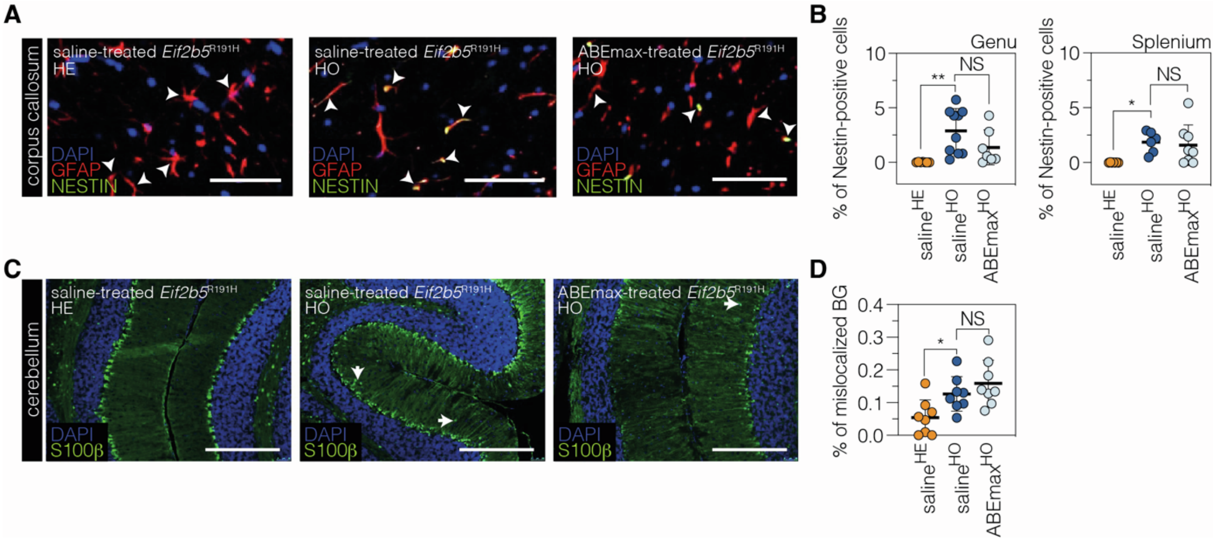

Figure S9 | Histological analysis of VWM neuropathologies in saline- and ABEmax-treated female *Eif2b5*$^{\mathbf{R191H}}$ mice. (**A,B**) Representative fluorescence micrographs (A) and the corresponding quantifications (B) of immature astrocytes (NESTIN/GFAP double positive) in the anterior (Genu) and posterior (Splenium) part of the corpus callosum in saline-treated heterozygous (n=7; HE), saline-treated homozygous (n=10; HO), and ABEmax-treated homozygous *Eif2b5*^{R191H} female mice (n=7; HO). Astrocytes are indicated by a white arrowhead. (**C,D**) Representative fluorescence micrographs (C) and the corresponding quantifications (D) of mislocalized Bergmann glia (S100β-positive) in the cerebellum of saline-treated heterozygous (n=8; HE), saline-treated homozygous (n=8; HE), and ABEmax-treated homozygous *Eif2b5*^{R191H} mice (n=8; HO). Mislocalized Bergmann glia are indicated by a white arrow. Scale bars, 50µm (A,C). Data are displayed as means ± SD of 8 mice per group and were analyzed using a one-way ANOVA with Dunnett's multiple comparisons test (NS, *P*>0.05; **P<0.05*; ***P<0.005*). Each datapoint represents one animal. NS, not significant; HE, heterozygous; HO, homozygous. Related to figure 5.

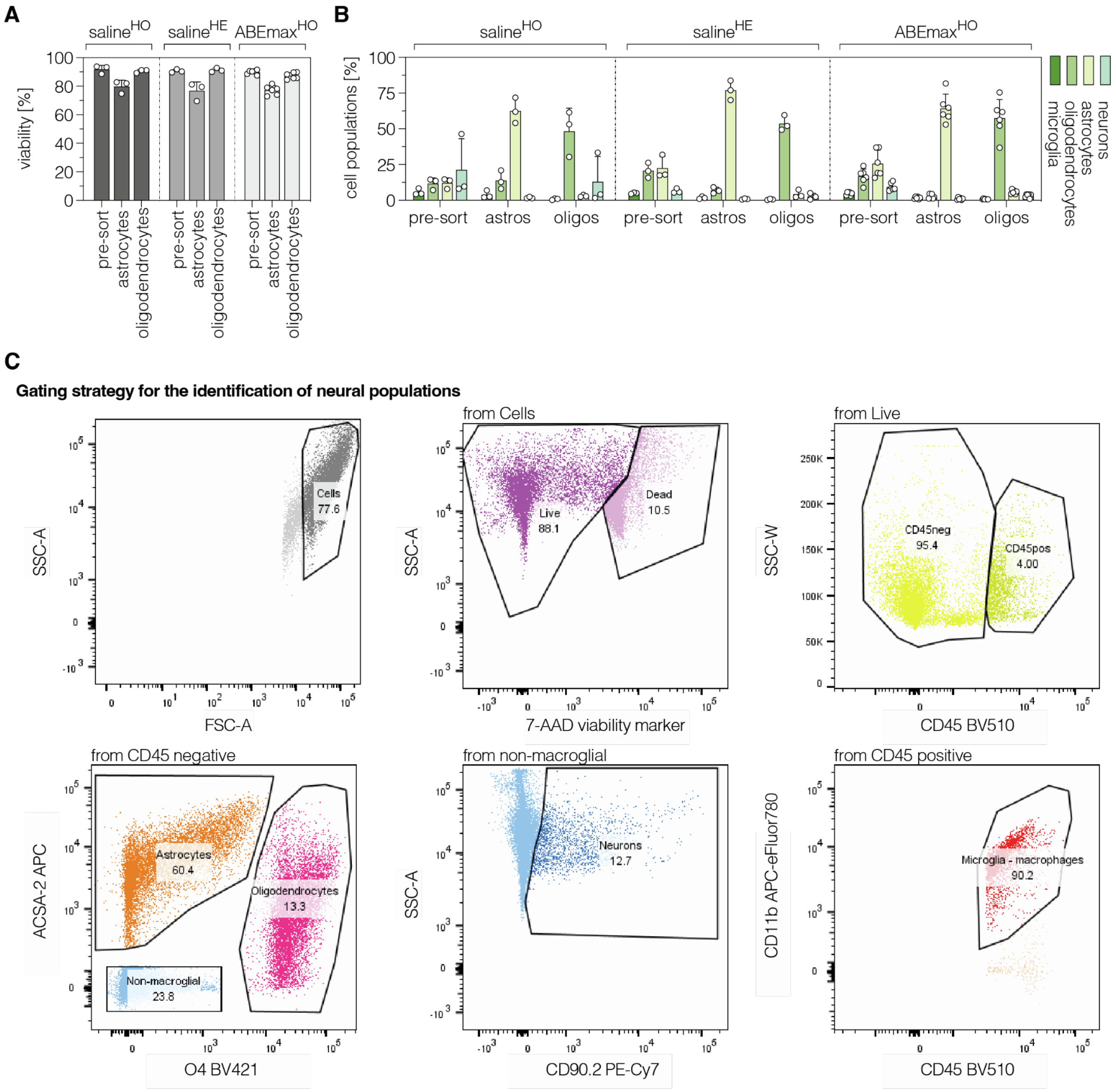

Figure S10 | Flow cytometry validation of *Eif2b5*^{R191H} brain cell populations. (**A**) Viability of cells before magnetic sorting (pre-sort), purified astrocytes, and purified oligodendrocytes isolated from *Eif2b5*^{R191H} mice. (**B**) Quantification of neuronal, astrocytic, oligodendrocytic, and microglia/macrophage populations before (pre-sort) and after magnetic sorting ("astros" and "oligos") by flow cytometry. (**C**) Gating strategy used for the quantification and identification of neural populations (neurons, astrocytes, oligodendrocytes, and microglia/macrophages). All mice were females except for one male in the saline-treated heterozygous control group (A,B). HO, homozygous; HE, heterozygous; astros, astrocytes; oligos, oligodendrocytes. Data are displayed as means ± SD of 3 or 6 mice per group. Related to figure 5.

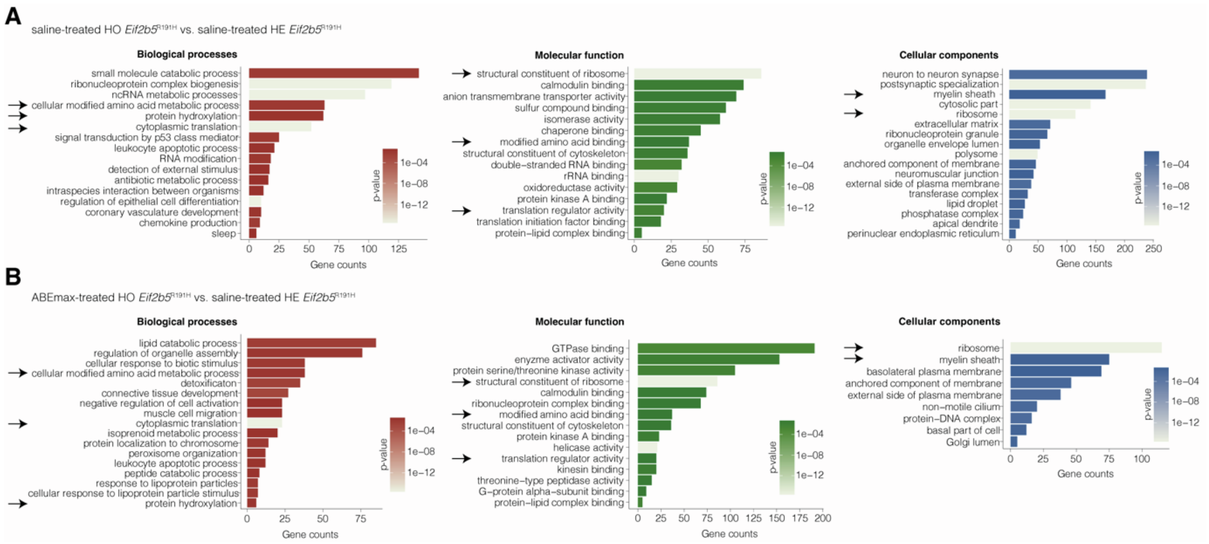

Figure S11 | Gene ontology analyses of saline- and ABEmax-treated female *Eif2b5*^{R191H} mice. (**A**,**B**) Gene ontology analyses of differentially expressed proteins in saline- (A; n=3) or ABEmax-treated female homozygous (HO) *Eif2b5*^{R191H} (B) mice (n=6). Both groups were compared to healthy heterozygous (HE) *Eif2b5*^{R191H} controls (n=3 female mice). Enriched terms are ranked by gene count and are color-coded with respect to their *p*-value. Terms associated with ribosome, translation, and myelination, which were found in both conditions, are indicated with a black arrow. HO, homozygous; HE, heterozygous. Related to figure 5.

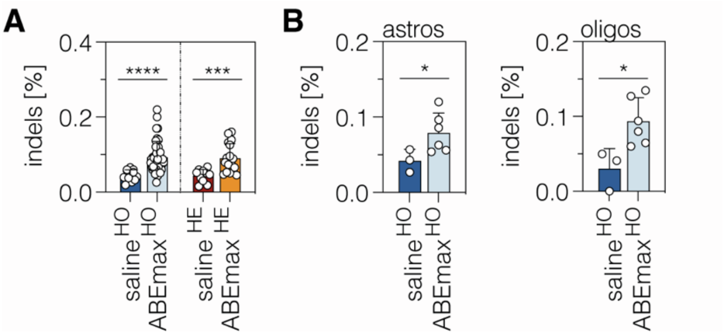

Figure S12 | Indel formation in saline- and ABEmax-treated *Eif2b5*^{R191H} mice. (**A,B**) Indel formation at the *Eif2b5* target site in saline- and ABEmax-treated animals (A; olfactory bulbs from female and male mice at experimental endpoints) or MACS-purified astrocytes and oligodendrocytes (B; only female mice at experimental endpoints). Indel quantification window spans from the nicking site to the deamination site. Data are displayed as means ± SD of at least 3 animals per group and were analyzed using an unpaired two-tailed Student's t-test (A,B; **P*<0.05; ****P*<0.0005; *****P*<0.0001). HE, heterozygous; HO, homozygous; astros, astrocytes; oligos, oligodendrocytes. Related to figure 6.

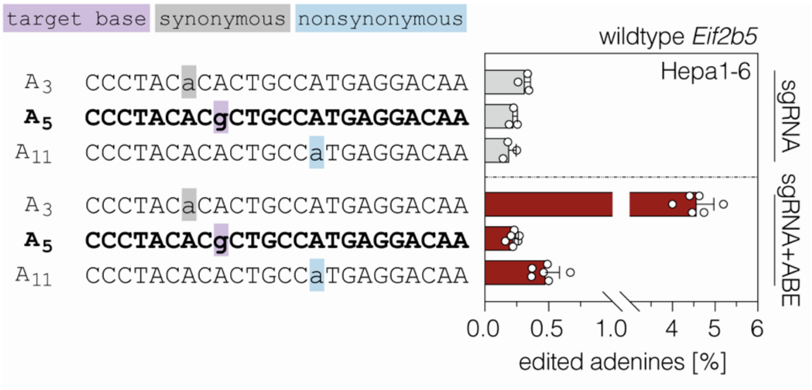

Figure S13 | *In vitro* bystander editing at the murine *Eif2b5* wildtype locus. Bystander editing at the wildtype locus (1 mismatch at position A_5) in murine Hepa1-6 cells. Data are displayed as means ± SD of at least 3 independent experiments. Related to figure 6.

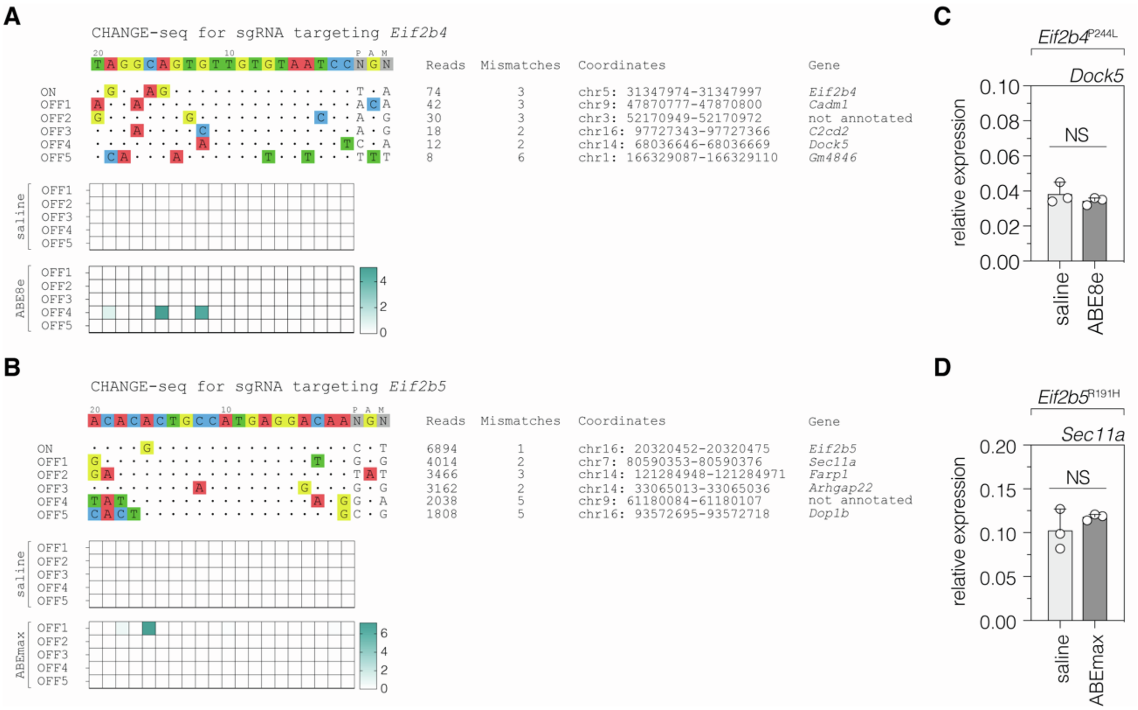

Figure S14 | *In vivo* base editing does not induce off-target editing in coding regions. (**A,B**) The top 5 off-target sites for the sgRNA protospacers targeting either the *Eif2b4* (A) or *Eif2b5* (B) locus were experimentally identified by CHANGE-seq.[43] Only the top 5 off-target sites are shown. Deep sequencing results of the top 5 off-target sites are shown for the *Eif2b4* (A) or *Eif2b5* sgRNA (B) in saline- and ABEmax/ABE8e-treated mice (>15'000 reads per site, n=3 mice per group). Data are displayed as means. (**C,D**) Transcript levels of off-target 4 (*Dock5*) in saline- and ABE8e-treated *Eif2b4*^{P244L} mice (C) and off-target 1 (*Sec11a*) in saline- and ABEmax-treated *Eif2b5*^{R191H} mice (D). Transcript levels were normalized to *Akt*. Data are displayed as means ± SD of 3 animals per group (A-D) and were analyzed using an unpaired two-tailed Student's t-test (C,D). Related to figure 6.

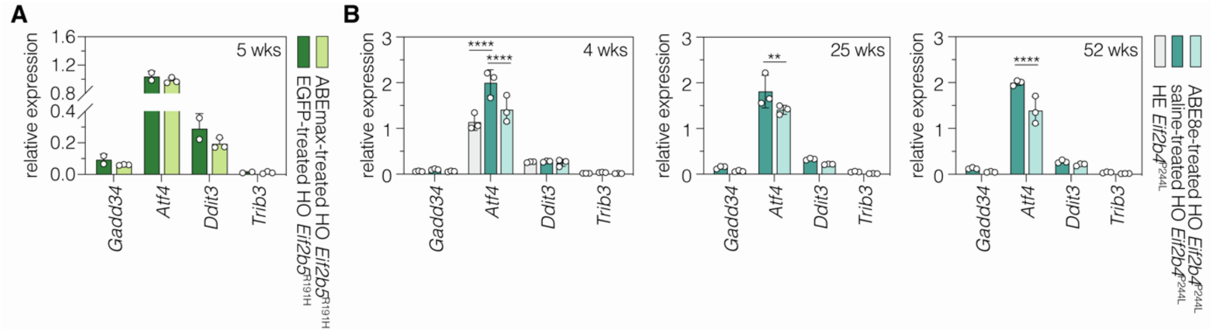

Figure S15 | AAV delivery and transgene expression do not upregulate the ISR in *Eif2b4*^{P244L} and *Eif2b5*^{R191H} mice. (**A**) Transcript levels of ISR regulators in homozygous *Eif2b5*^{R191H} animals at 5 weeks after delivery of AAV-PHP.eB particles, expressing either EGFP or ABEmax under the Cbh promoter, at a dose of 8×10^{10} vg per animal via ICV injection. (**B**) Transcript levels of ISR regulators in homozygous *Eif2b4*^{P244L} animals at 4, 25, and 52 weeks after delivery of AAV-PHP.eB particles, expressing ABE8e under the Cbh promoter, at a dose of 4.8×10^{10} vg per animal via ICV injection. Transcript levels of healthy heterozygous *Eif2b4*^{P244L} (4 weeks) and saline-treated homozygous *Eif2b4*^{P244L} (4, 25, and 52 weeks) mice are shown for comparison. All transcripts were normalized to *Akt*. Data are displayed as means ± range (A) or means ± SD (B) of 2 or 3 animals per group. HO, homozygous. Related to figure 6.

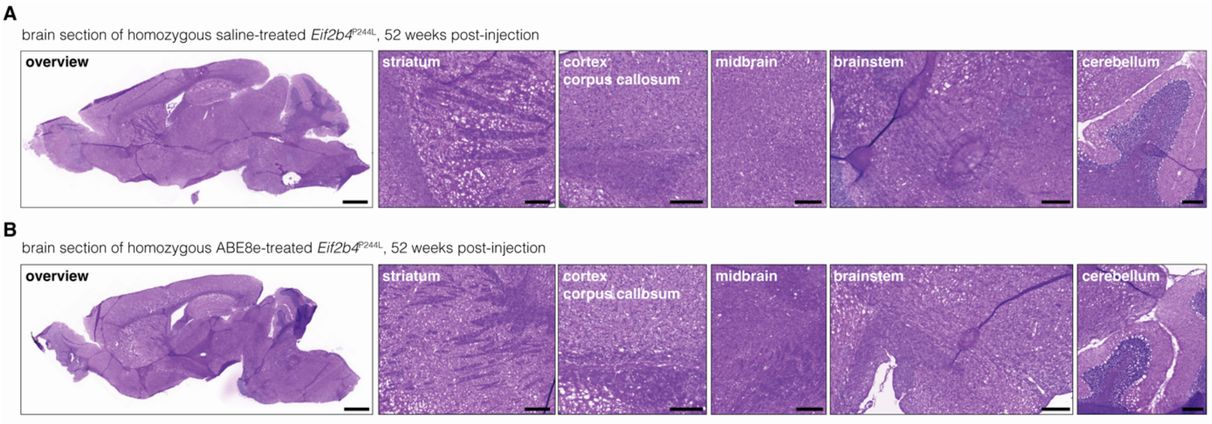

Figure S16 | *In vivo* base editing does not induce tissue damage in brains of homozygous *Eif2b4*$^{\mathbf{P244L}}$ mice. (**A,B**) Representative whole brain overview images and close-ups of distinct regions of hematoxylin- and eosin-stained brain sections of saline- (A) and ABE8e-treated homozygous mice (B). Scale bars, 1000µm (overview); 100µm (magnified view). Related to figure 6.

Supplemental tables

Table S1 | On- and off-target efficiency predictions for sgRNAs designed using the indicated algorithms. A score above 50 is considered good. IDT, integrated DNA technologies; Bench, Benchling; ON-T, on-target efficiency; OFF-T, off-target efficiency.

sgRNA_ID	sequence (5′ to 3′)	$ON\text{-}T^{IDT}$	$ON\text{-}T^{Bench}$	$OFF\text{-}t^{CRISPOR}$	$OFF\text{-}t^{IDT}$	$OFF\text{-}t^{Bench}$
sgRNA_01	CTGGAGAGTTCCTCACTGGG	53/100	67/100	75/100	18/100	36/100
sgRNA_02	CTCCCAGTGAGGAACTTCTCC	69/100	54/100	77/100	29/100	39/100

Table S2 | List of antibodies used in this study.

antibody	clone/ID	host	dilution	application
ACSA-2/APC	IH3-18A3	rat	0.75μg/mL	flow cytometry
CD11b/APC-eFluor™ 780	M1/70	rat	2μg/mL	flow cytometry
CD45/BV 510™	30-F11	rat	2μg/mL	flow cytometry
CD90.2/PE(Cy7)	30-H12	rat	2μg/mL	flow cytometry
O4/Biotin	O4	mouse	2.2μg/mL	flow cytometry
NEUN	EPR12763	rabbit	1:1’000	histology
GFAP	ab95231	chicken	1:1’500	histology
NESTIN	25/NESTIN	mouse	1:500	histology
GFAP	Z0334	rabbit	1:1’000	histology
S100β	15146-1-AP	rabbit	1:1’000	histology
anti-rabbit A488	JIR-711-545-152	donkey	1:1’000	histology
anti-chicken Cy5	JIR-703-175-155	donkey	1:500	histology
anti-rabbit A594	A31632	goat	1:1’000	histology
anti-mouse A488	A31620	goat	1:1’000	histology

Table S3 | Reference nucleotide sequences of HTS amplicons.

amplicon_ID	amplicon sequence (5′ to 3′)
EIF2B4-PB	GGATCCACTAGTAACGGCCGCCAGTGTGCTGGAATTCGAAGCAATGACTCTCACAATCT<br>GAACACTTACTTCCCTAGTGAAGCTTACCCCTGTCCCATAGTAGAGATCTTCCTTAAGG<br>GGTGTTTTGTATTCTCTAGGTGATTCAGGATTACACAACACtgCCtAGTGAGGAACTCT<br>CCAGGGATCTTGTAAATAAACTAAAACCCTACATCAGGTGAGCACAGCC
EIF2B5-PB	GATCACAGAGTTGGGCAGACTAACTGTGCCTCTGGTTCTTAATAGGTTAAGAAGGAAGC<br>TAGAAAAAAATGTCTCTGTGATGACAATGGTCTTCAAAGAGTCGTCACCCAGCCACCCT<br>ACACACTGCCATGAGGACAACGTGGTGATGGCTGTGGACAGCGCCACCAACAGGGTTCT<br>TCACTTCCAGAAGACCCAAGG
EIF2B4-mouse	GTGAAGCTTACCCCTGTCCCATAGTAGAGATCTTCCTTAAGGGGTGTTTTGTATTCTCT<br>AGGTGATTCAGGATTACACAACACtgCCtAGTGAGGAACTCTCCAGGGATCTTGTAAAT<br>AAACTAAAACCCTACATCAGGTGAGCACAGCC
EIF2B5-mouse	GGACAAGACTTAGGGACCAGCAGAGTCCTAAGACTAACTGTGCCTCTGGTTCTTAATAG<br>GTTAAGAAGGAAGCTAGAAAAAAATGTCTCTGTGATGACAATGGTCTTCAAAGAGTCGT<br>CACCCAGCCACCCTACACACTGCCATGAGGACAACGTGGTGATGGCTGTGGACAGCGCC<br>ACCAACAGGGTTCTTCACTTCCAGAAGACCCAAGGCCTCCGGCGCTTTTCCTTTCCATT

Supplemental Methods

Table S4 | Oligos used for cloning of sgRNA plasmids.

oligo name	sgRNA_ID	sequence (5′ to 3′)
sgRNA01_P244L-NGN_fwd	sgRNA-01	CACCgTaGGcaGTGTTGTGTAATCC
sgRNA01_P244L-NGN_rev		AAACGGATTACACAACACtgCCtAc
sgRNA02_P244L-NNGRRT_fwd	sgRNA-02	CACCgACTaGGcaGTGTTGTGTAATC
sgRNA02_P244L-NNGRRT_rev		AAACGATTACACAACACtgCCtAGTc
sgRNA03_R191H_NNNRRT_fwd	sgRNA-03	CACCGTACACACTGCCATGAGGACAA
sgRNA03_R191H_NNNRRT_rev		AAACTTGTCCTCATGGCAGTGTGTAC
sgRNA04_R191H_NGG_fwd	sgRNA-04	CACCGACACTGCCATGAGGACAACG
sgRNA04_R191H_NGG_rev		AAACCGTTGTCCTCATGGCAGTGTC
sgRNA05_R191H_NGN_fwd	sgRNA-05	CACCGACACACTGCCATGAGGACAA
sgRNA05_R191H_NGN_rev		AAACTTGTCCTCATGGCAGTGTGTC

Table S5 | Oligos used for cloning of AAV plasmids.

oligo name	sequence (5′ to 3′)
pCMV-N_fwd	CGGCCTCTAGATCAGGGTACcgacattgattattgactagttattaatag
pCMV-N_rev	ggtggcggccagctctgcttatatagacc
Tad8e_fwd	aagcagagctggccgccaccatgaaacg
Tad8e_rev	cctccggagttgatggagctctgggccttcttctgagc
pCMV-C_fwd	ACTAGGGGTTCCTGCGGCCTCTAGATCAGGGTACCgacattgattattg
pCMV-C_rev	CTGTCCGTTTCATGGTGGCgctagcagctctgcttatatagacctc
pCbhTad8e_fwd	tcactttttttcaggttggaccggtgccaccatgaaacggacag
pCbhTad8e_rev	ggtgtccttgctcagctgcagtttggcatcctcggccag
pCbh-cSpG_fwd	agaatggctttatcgccagcaattgcttcgactccgtggaaatctccgg
pCbh-cSpG_rev	tcctcttcttcttgggctcgaattcgctgccgtcggcggttct

Table S6 | Oligos used for validation of sgRNAs and *Eif2b4*^{P244L} knock-in.

oligo name	sequence (5′ to 3′)
19017.P1_fwd	CATCCCATCCTCTGTGATCC
19017.P4_rev	TGCTCCCCTAAGCCTGACTA
19017.P5_fwd	GATGGTCCTCTCTGGGACAA
19017.P6_rev	GTCAGGAAGCTGCAACAACA

Table S7 | Oligos used for RT-qPCR.

oligo name	sequence (5′ to 3′)
Gfap-RTqPCR_fwd	ACATCGAGATCGCCACCTACA
Gfap-RTqPCR_rev	CCACGATGTTCCTCTTGAGGTG
Gfapα-RTqPCR_rev	CCTTCACATCACCACGTCCTTG
Gfapδ-RTqPCR_rev	CCATTTTCAATCTGGTGAGCCTG
Nestin-RTqPCR_fwd	CTACAGAGTCAGATCGCTCAG
Nestin-RTqPCR_rev	AGCAGAGTCCTGTATGTAGC
Mbp-RTqPCR_fwd	ATTCACCGAGGAGAGGCTGGAA
Mbp-RTqPCR_rev	TGTGTGCTTGGAGTCTGTCACC
Plp1-RTqPCR_fwd	CCTAGCAAGACCTCTGCCAGTA
Plp1-RTqPCR_rev	GGACAGAAGGTTGGAGCCACAA
Mog-RTqPCR_fwd	GATGAAGGAGGCTACACCTGCT
Mog-RTqPCR_rev	CGTAGGCACAAGTGCGATGAGA
Olig2-RTqPCR_fwd	ATGCACGACCTCAACATCGCCA
Olig2-RTqPCR_rev	ACCAGTCGCTTCATCTCCTCCA
Pdgfrα-RTqPCR_fwd	CTGGAGAAGTGAGAAACAAAGG
Pdgfrα-RTqPCR_rev	TGGACAGAAATGGTGACTC
Gapdh-RTqPCR_fwd	CATCACTGCCACCCAGAAGACTG
Gapdh-RTqPCR_rev	ATGCCAGTGAGCTTCCCGTTCAG
Cas9-N-RTqPCR_fwd	aaacagcagattcgcctgga
Cas9-N-RTqPCR_rev	tcatccgctcgatgaagctc
Nintein-RTqPCR_fwd	ttcatgacagtggatggcca
Nintein-RTqPCR_rev	ttcttgggctcaaactcact
Cas9-C-RTqPCR_fwd	atgggcgggatatgtacgtg
Cas9-C-RTqPCR_rev	ggtcagcaccttgttgtcga
Cintein-RTqPCR_fwd	agtcaccaaagaagaagcgga
Cintein-RTqPCR_rev	gaagttgtgatcccgctcca
Hprt-RTqPCR_fwd	GTTGGGCTTACCTCACTGCT
Hprt-RTqPCR_rev	TAATCACGACGCTGGGACTG
Akt-RTqPCR_fwd	AAGAAGGAGGTCATCGTCGC
Akt-RTqPCR_rev	GGTCGTGGGTCTGGAATGAG
Gadd34-RTqPCR_fwd	GCTCAGATTGTTCAAAGCCC
Gadd34-RTqPCR_rev	CTTTCTCAGCGAAGTGTACC
Atf4-RTqPCR_fwd	CTAGGTCTCTTAGATGACTATCTG
Atf4-RTqPCR_rev	CTCCAACATCCAATCTGTCC
Ddit3/Chop-RTqPCR_fwd	CTGGTATGAGGATCTGCAGG
Ddit3/Chop-RTqPCR_rev	TTGATTCTTCCTCTTCGTTTCC
Trib3-RTqPCR_fwd	TGTCTTCAGCAACTGTGAGAGGACGAAG
Trib3-RTqPCR_rev	GTAGGATGGCCGGGAGCTGAGTATC

Table S8 | Oligos used for deep sequencing.

oligo name	oligo sequence (5′ to 3′)
HTS-EIF2B4-PB_fwd	CTTTCCCTACACGACGCTCTTCCGATCTNNNNNNNGGATCCACTAGTAACGGCCG
HTS-EIF2B4-PB_rev	GGAGTTCAGACGTGTGCTCTTCCGATCTNNNNNNNTCCCTGGAGAGTTCCTCACT
HTS-EIF2B5-PB_fwd	CTTTCCCTACACGACGCTCTTCCGATCTNNNNNNNGATCACAGAGTTGGGCAGAC
HTS-EIF2B5-PB_rev	GGAGTTCAGACGTGTGCTCTTCCGATCTNNNNNNNCCTTGGGTCTTCTGGAAGTG
HTS-EIF2B4m_fwd	CTTTCCCTACACGACGCTCTTCCGATCTNNNNNNNGTGAAGCTTACCCCTGTCCC
HTS-EIF2B4m_rev	GGAGTTCAGACGTGTGCTCTTCCGATCTNNNNNNNGGCTGTGCTCACCTGATGTA
HTS-EIF2B5m_fwd	CTTTCCCTACACGACGCTCTTCCGATCTNNNNNNNGGACAAGACTTAGGGACCAG
HTS-EIF2B5m_rev	GGAGTTCAGACGTGTGCTCTTCCGATCTNNNNNNNAATGGAAAGGAAAAGCGCC

Table S9 | Oligos used for CHANGE-seq.

oligo name	oligo sequence (5′ to 3′)
amplCHANGE-2b4_fwd	CCATTCCCTAAGACTGCCCC
amplCHANGE-2b4_rev	GGGAGGTCAGAAGATGCTGC
amplCHANGE-2b5_fwd	GAGGAACACAGGTCAGGCTG
amplCHANGE-2b5_rev	ATTACAGACCCCCGAGAGCT
HTS-2b4_OFF1_fwd	CTTTCCCTACACGACGCTCTTCCGATCTTGTCACTGCGGTTTCTCG
HTS-2b4_OFF1_rev	GGAGTTCAGACGTGTGCTCTTCCGATCTCCATACACTGCCTACCGCC
HTS-2b4_OFF2_fwd	CTTTCCCTACACGACGCTCTTCCGATCTTCGACCAGTTAGAGACCAGCCT
HTS-2b4_OFF2_rev	GGAGTTCAGACGTGTGCTCTTCCGATCTGGCCCATGTGCCAGAAAATAAGA
HTS-2b4_OFF3_fwd	CTTTCCCTACACGACGCTCTTCCGATCTACAGTTGTTCTCATTCATGTGGGA
HTS-2b4_OFF3_rev	GGAGTTCAGACGTGTGCTCTTCCGATCTAAAGTTGGGCATGTTGGAATACA
HTS-2b4_OFF4_fwd	CTTTCCCTACACGACGCTCTTCCGATCTTTTTCCAAGTTTCAATTAACTGCCT
HTS-2b4_OFF4_rev	GGAGTTCAGACGTGTGCTCTTCCGATCTACCTTACCTGTTTGTTGGGGAG
HTS-2b4_OFF5_fwd	CTTTCCCTACACGACGCTCTTCCGATCTCCCATGTAGTATCCAAATGTCACC
HTS-2b4_OFF5_rev	GGAGTTCAGACGTGTGCTCTTCCGATCTAGCATACTGATTACTTCTTGATGGT
HTS-2b5_OFF1_fwd	CTTTCCCTACACGACGCTCTTCCGATCTAGAGAGAGAGACACCATGACCA
HTS-2b5_OFF1_rev	GGAGTTCAGACGTGTGCTCTTCCGATCTATCCCAAGCTATACCCAGTGG
HTS-2b5_OFF2_fwd	CTTTCCCTACACGACGCTCTTCCGATCTGCCACACTTCTGACCTGACA
HTS-2b5_OFF2_rev	GGAGTTCAGACGTGTGCTCTTCCGATCTCTCAGGCTGCAGGTGACTAC
HTS-2b5_OFF3_fwd	CTTTCCCTACACGACGCTCTTCCGATCTTCTCCGAGTTGCCCTAGAGT
HTS-2b5_OFF3_rev	GGAGTTCAGACGTGTGCTCTTCCGATCTAGACAAGGCTTACTTCGGGG
HTS-2b5_OFF4_fwd	CTTTCCCTACACGACGCTCTTCCGATCTCCATAGATGGGGAGAACCCAA
HTS-2b5_OFF4_rev	GGAGTTCAGACGTGTGCTCTTCCGATCTAACACACTCTCCACTCTTGACTAA
HTS-2b5_OFF5_fwd	CTTTCCCTACACGACGCTCTTCCGATCTGTCTACCGTGGAGCATGAGG
HTS-2b5_OFF5_rev	GGAGTTCAGACGTGTGCTCTTCCGATCTCACTCTGCTCTGCTTCAGCC

Supplemental References

65. Paxinos, G, Franklin, KBJ (2001). The Mouse Brain in Stereotaxic Coordinates, 2nd edition. *Acad Press*.
